# Long Span DNA Paired-End-Tag (DNA-PET) Sequencing Strategy for the Interrogation of Genomic Structural Mutations and Fusion-Point-Guided Reconstruction of Amplicons

**DOI:** 10.1371/journal.pone.0046152

**Published:** 2012-09-28

**Authors:** Fei Yao, Pramila N. Ariyaratne, Axel M. Hillmer, Wah Heng Lee, Guoliang Li, Audrey S. M. Teo, Xing Yi Woo, Zhenshui Zhang, Jieqi P. Chen, Wan Ting Poh, Kelson F. B. Zawack, Chee Seng Chan, See Ting Leong, Say Chuan Neo, Poh Sum D. Choi, Song Gao, Niranjan Nagarajan, Hervé Thoreau, Atif Shahab, Xiaoan Ruan, Valère Cacheux-Rataboul, Chia-Lin Wei, Guillaume Bourque, Wing-Kin Sung, Edison T. Liu, Yijun Ruan

**Affiliations:** 1 Genome Institute of Singapore, Agency for Science, Technology and Research, Singapore, Singapore; 2 Department of Epidemiology and Public Health, Yong Loo Lin School of Medicine, National University of Singapore, Singapore, Singapore; 3 Graduate School for Integrative Sciences and Engineering, Centre for Life Sciences, National University of Singapore, Singapore, Singapore; 4 Department of Biochemistry, Yong Loo Lin School of Medicine, National University of Singapore, Singapore, Singapore; Leuven University, Belgium

## Abstract

Structural variations (SVs) contribute significantly to the variability of the human genome and extensive genomic rearrangements are a hallmark of cancer. While genomic DNA paired-end-tag (DNA-PET) sequencing is an attractive approach to identify genomic SVs, the current application of PET sequencing with short insert size DNA can be insufficient for the comprehensive mapping of SVs in low complexity and repeat-rich genomic regions. We employed a recently developed procedure to generate PET sequencing data using large DNA inserts of 10–20 kb and compared their characteristics with short insert (1 kb) libraries for their ability to identify SVs. Our results suggest that although short insert libraries bear an advantage in identifying small deletions, they do not provide significantly better breakpoint resolution. In contrast, large inserts are superior to short inserts in providing higher physical genome coverage for the same sequencing cost and achieve greater sensitivity, in practice, for the identification of several classes of SVs, such as copy number neutral and complex events. Furthermore, our results confirm that large insert libraries allow for the identification of SVs within repetitive sequences, which cannot be spanned by short inserts. This provides a key advantage in studying rearrangements in cancer, and we show how it can be used in a fusion-point-guided-concatenation algorithm to study focally amplified regions in cancer.

## Introduction

Human genome alterations are variable and range from whole chromosome gains or losses, to sub-chromosomal changes of deletions, tandem duplications, inversions, insertions and translocations to the base pair level alterations including small insertions/deletions (indels) and single nucleotide variations (SNVs). Studies on SNVs have helped us to understand their functional roles in normal traits and diseases [1]. However, our knowledge of the functional role of large scale structural variations (SVs) is still limited. Traditional cytogenetic methods can detect only copy number changes at megabase to sub-megabase levels [2], [3]. Although SAGE-based digital karyotyping [4], [5], and whole-genome tiling arrays [6] may detect structural changes at kilobase to sub-kilobase levels, copy number neutral SVs such as inversions, ‘cut and paste insertions’ and balanced translocations cannot be uncovered by these methods. Conventional paired-end sequencing of large genomic DNA inserts in bacterial artificial chromosome (BAC) and fosmid clones can detect hundreds of genome variations at a resolution of 40 kb or 100 kb [7], [8]. However, the high cost, complicated process and low throughput of such efforts is prohibitive.

Next-generation sequencing of paired-end-tags from genomic DNA fragments (DNA-PET) is an ideal method for studying human genome SVs, in which only the short 5′ and 3′ tags of specific size range DNA fragments are sequenced in a massive and highly parallel manner. The DNA-PET sequences obtained are then mapped to the reference genome to identify potential genomic breakage/fusion points between the test and the reference genomes using discordant tag mapping patterns as readout. This concept was initially introduced by Snyder and his colleagues [9] in an effort to study human genome variation in lymphoblastoid cell lines derived from an African and a European individual using Roche 454 pyrosequencing to generate PET sequences from 3 kb fragments. This was followed by studies on cancer genome rearrangements using paired-end sequencing of short DNA fragments (a few hundred base pairs) [10], [11], [12], [13], [14], [15], [16], [17], [18], or by combination of short and a few kilobase pair fragments [19], [20].

Though these studies have provided significant insights about human normal and cancer genome SVs, and the evolution of the cancer genome associated with the disease progression, there are important aspects of PET sequencing that still remain unexplored. One of the unanswered questions is the choice of insert size of a sequencing library that is best suited to identify structural variations in human genomes. In theory, a small insert size (sub-kilobase) has the advantage of tight size selection of DNA fragments and greater sensitivity for small intra-chromosome rearrangements. In contrast, larger insert sizes (kilobase to tens of kilobases) have the advantage of higher physical coverage of the genome with the possible drawback of less precise localization of the breakpoint regions.

To address this question, we used three well established cancer cell lines including the breast cancer cell line MCF-7, the colon cancer cell line HCT116 and the chronic myelogenous leukemia (CML) cell line K562 as test genomes and constructed DNA-PET sequencing libraries with variable insert sizes (1 kb, 10 kb and 20 kb) to identify different categories of genomic SVs. The comparison of SVs identified by different insert sizes with the same sequencing effort suggested that larger insert sizes can uncover more and larger SVs in practice. As expected, small insert sizes have an advantage in detecting small deletions (<5 kb). Breakpoint resolution examination by genomic PCR and Sanger sequencing showed that large and small insert size libraries have comparable precision in locating breakpoints. Large insert sizes also enabled better identification of SVs within repetitive sequences and based on a fusion-point-guided-concatenation algorithm we used the large insert libraries to study the amplicon surrounding the *BCR-ABL1* fusion gene in K562, as well as other amplicons in K562, MCF7 and HCT116.

## Results

### DNA-PET library construction and sequencing

**Figure 1:**
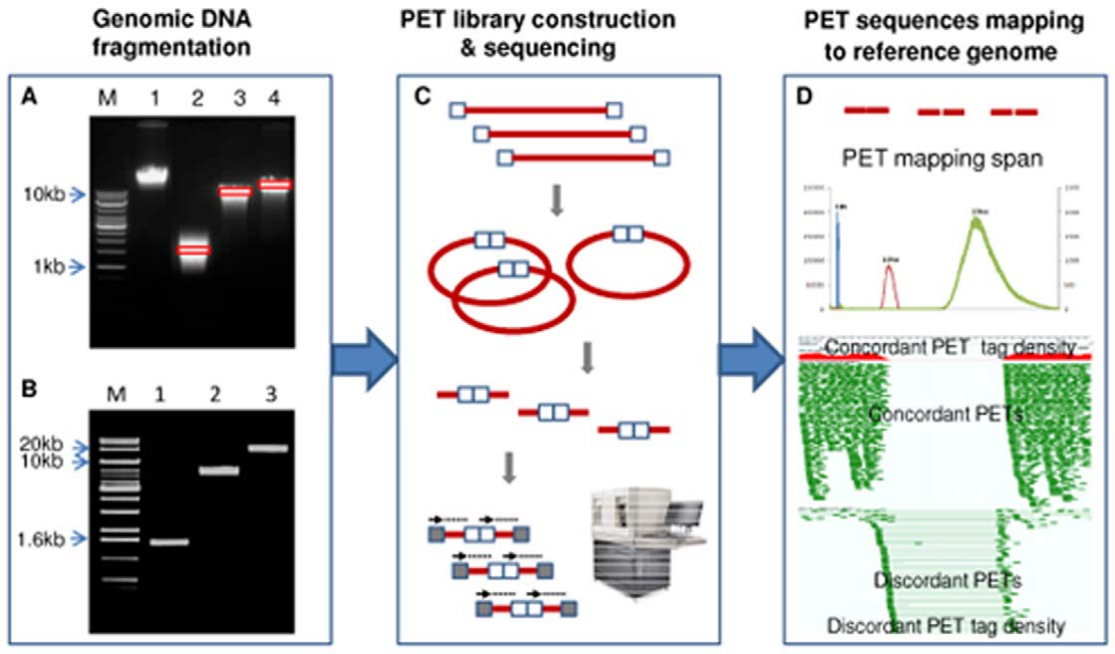
DNA-PET library construction, sequencing and mapping. (A) The genomic DNA was randomly sheared to different size range. (B) The very narrow region DNA fragments were obtained after size selection. (C) The purified DNA fragments were circularized, *EcoP15I* digested, sequencing adaptor ligated, and finally sequenced by SOLiD sequencer. (D) PET mapping span distribution of 1 kb (blue), 10 kb (red) and 20 kb (green) libraries. Based on the mapping pattern, PETs can be distinguished as concordant PETs and discordant PETs.

By using three cancer cell lines, we generated seven genomic DNA-PET datasets, two each for MCF-7 and HCT116 (1 kb and 10 kb) and three for K562 (1 kb, 10 kb and 20 kb, Table 1; more details in Table S1). DNA-PET sequencing libraries were constructed as described in Methods and Protocol S1. Critical steps are the fragmentation of the genomic DNA by hydroshearing, fragment size selection and DNA extraction after electrophoresis on large low percentage agarose gels, and circularization by biotinylated internal adapters in highly diluted reactions which is followed by massive parallel sequencing and mapping (Figure 1).

**Table 1:** Statistics of PET sequencing.

Library	Total NR1) PET	Coverage2)	cPET3)	% of cPET4)	dPET5)	% of dPET6)	dPET Singletons	dPET clusters (≥3)	# of dPET included7)
MCF-7 1 kb	18,335,127	8.2	17,598,541	96.0	736,586	4.0	728,445	1,024	6,811
MCF-7 10 kb	18,432,387	69.3	16,292,711	88.4	2,139,676	11.6	2,119,094	1,029	19,314
HCT116 1 kb	20,613,096	9.4	18,919,833	91.8	1,693,263	8.2	1,688,547	572	3,866
HCT116 10 kb	20,610,717	58.5	17,938,263	87.0	2,672,454	13.0	2,661,375	756	8,893
K562 1 kb	41,996,278	15.4	39,818,591	94.8	2,177,687	5.2	2,157,688	1,717	14,569
K562 10 kb	34,759,699	91.9	31,240,240	89.9	3,519,459	10.1	3,495,217	1,383	24,242
K562 20 kb	38,393,242	277.7	35,246,258	91.8	3,146,984	8.2	3,103,814	2,191	32,282

1) non-redundant.

2) physical coverage.

3) concordant PET.

4) proportion of cPET to total NR PET.

5) discordant PET.

6) proportion of dPET to total NR PET.

7) number of dPET involved in the dPET clusters with cluster count ≥3.

To get comparable non-redundant PET numbers among different insert size libraries of the same genome, we randomly selected a subset of original MCF-7 and HCT116 10 kb libraries[21] resulting in approximately 20 million non-redundant (NR) PET sequences for all MCF-7 and HCT116 libraries. Given the comparable non-redundant and uniquely-mapped PET sequences, the MCF-7 10 kb library resulted in approximately 69-fold physical (fragment) coverage of the human genome versus 8-fold of 1 kb library. If we would have sequenced the MCF-7 10 kb library only by one Solid slide as we did for the 1 kb library, down sampling would not have been necessary and sequencing costs and depth (nucleotides sequenced) would have been the same for 10 kb and 1 kb libraries. Of the PET sequences in the 10 kb library, 88% (16,292,711 of 18,432,387) were mapped concordantly to the reference genome (hg18, NCBI Build 36) as concordant PETs (cPETs) and this proportion of PETs reflected the agreement of the genome architecture between MCF-7 and the reference genome. In contrast, 11.6% (2,139,676) of the uniquely-mapped PETs were mapped as discordant PETs (dPETs) and some of these dPETs were indicative of potentially rearranged genomic regions crossing the breakage/fusion junction points where the MCF-7 genome is different from the reference genome. In the MCF-7 1 kb dataset, 96% (17,598,541 of 18,335,127) NR PET were concordantly mapped to the reference genome and 4% (736,586) were discordant. The lower proportion of dPETs in the 1 kb compared to the 10 kb library is due to the sharper size selection and lower number of short span PET constructs which comprise the largest proportion of dPETs for 10 kb libraries [21].

### Large structural variations identified by different insert size libraries

**Figure 2:**
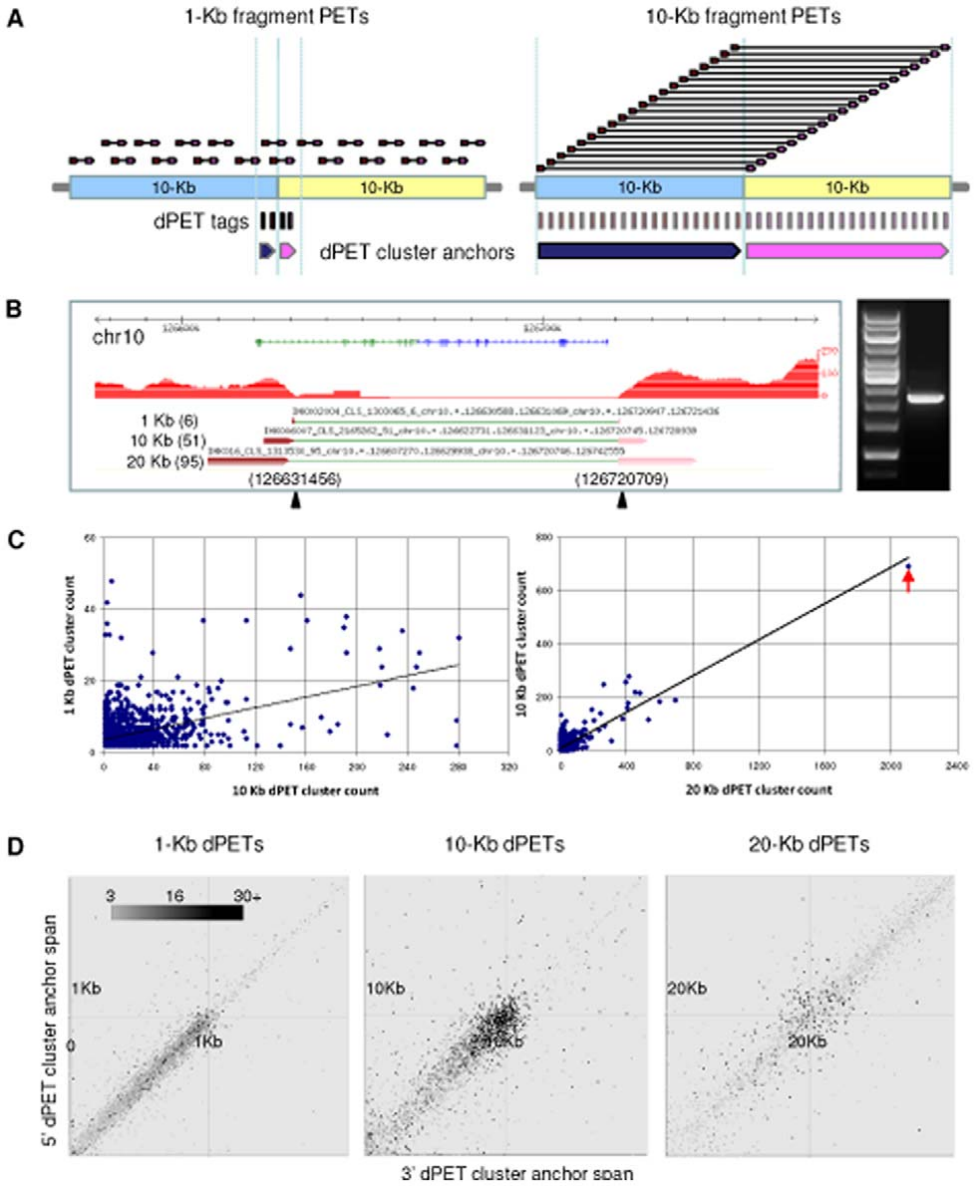
dPET cluster characteristics. (A) PET mapping overlap scheme based on the same number of PETs. 10 kb library dPETs have a higher chance than 1 kb library dPETs to cross the same breakpoints. (B) One common deletion identified in K562 by 1 kb, 10 kb and 20 kb libraries. Red track represents the coverage of the cPETs. The dPET cluster count in each library was 6, 51 and 95, respectively. The genomic PCR and Sanger sequencing confirmed the presence of the deletion and located the breakpoint positions to chr10:126,631,456 and chr10:126,720,709 (hg18). The differences between breakpoint position predicted by DNA-PET and PCR were 387 bp and 238 bp for the 1 kb library; 333 bp and 36 bp for the 10 kb library, and 482 bp and 37 bp in 20 kb library. (C) Cluster count correlation of the same set of SVs identified by both 1 kb and 10 kb libraries in the three genomes (left panel), and 10 kb and 20 kb libraries in K562 (right panel). The black line represents the trendline. The rearrangement between chromosomes 9 and 22 creating the CML causing *BCR-ABL1* fusion gene [23] was identified by the largest clusters of 692 dPETs in the 10 kb library and 2,106 dPETs in the 20 kb library (red arrow head). (D) Length distribution of 5′ and 3′ anchor regions (regions in which the tags of dPET clusters are mapping) in 1 kb, 10 kb and 20 kb libraries of K562. The 10 kb library showed a more even length distribution of 5′ and 3′ anchor regions which suggested more balanced mapping characteristics around breakpoints for 10 kb libraries.

**Figure 3:**
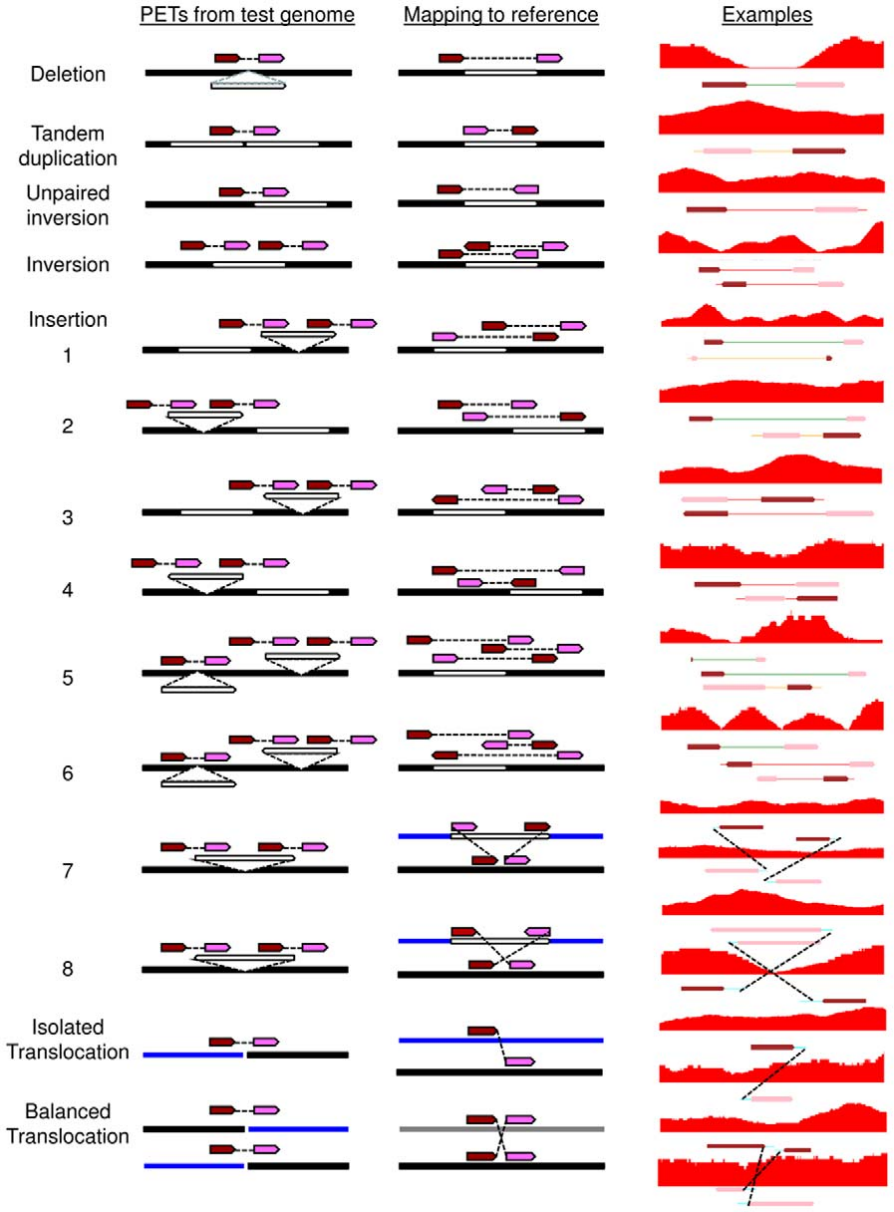
SV identification based on the mapping pattern of dPET clusters. The dark red and pink arrows represent the 5′ and 3′ anchor regions of the dPET cluster, respectively. Black, white and blue horizontal lines represent chromosome segments. The red track represents the coverage of cPETs. The dotted lines indicate the connections between the two dPET clusters. The sub-types of insertions are as follows: (1) Intra-chromosomal direct forward insertion. (2) Intra-chromosomal direct backward insertion. (3) Intra-chromosomal inverted forward insertion. (4) Intra-chromosomal inverted backward insertion. (5) Deletion plus intra-chromosomal direct forward insertion. (6) Deletion plus intra-chromosomal inverted forward insertion. (7) Inter-chromosomal direct insertion. (8) Inter chromosome inverted insertion.

Each of the dPETs could potentially map over a breakage/fusion point. However, it is inevitable that spurious dPET mappings would arise due to chimeric ligation during the construction of DNA-PET libraries or incorrect tag mapping. To reduce such random noise, we used the PET mapping overlap scheme [21] (Figure 2A) to discard all the singleton and cluster size 2 dPETs and considered only the dPET clusters with multiple overlapping PETs (≥3) as true signals for further analysis of genome rearrangements (Table S2, Figure S1). The large insert size and high sequencing depth of the libraries allowed for the identification of different types of SVs, including deletions, tandem duplications, inversions, unpaired inversions, isolated translocations, and balanced translocations. In addition and in contrast to previous studies, eight different sub-types of insertions were characterized in this study. The eight sub-types of insertions included different combinations of intra or inter-chromosomal, direct or inverted, forward or backward insertions (Figure 3, Table S3).

A high count for a dPET cluster (large number of dPETs spanning the same breakage/fusion point) gives high confidence for the rearrangement point and may also reflect the copy number of the breakage/fusion point. The highest dPET cluster count in the MCF-7 10 kb library was 766 and only 91 in the corresponding 1 kb library. We observed similar drops in cluster count for HCT116 (148 in the 10 kb library and 63 in the 1 kb library) and K562 (2,106 for the 20 kb library, 692 for the 10 kb library and 127 for the 1 kb library, respectively; Figure 2B and 2C). The total number of SVs identified in the 1 kb libraries of each genome was comparable to the number of SVs found in the 10 kb libraries; however, the composition of the SV types was different. In the 1 kb libraries, the vast majority of SVs was deletion (79% in MCF-7, 80% in HCT116, and 78% in K562); whereas in the 10 kb libraries, the percentage of deletions was much lower (33% in MCF-7, 59% in HCT116, and 38% in K562). In contrast, the number of inversions and insertions identified in 1 kb libraries was much lower than in 10 kb libraries (Table S4).

### Classification of isolated and complex SVs

Due to the complicated genomic architecture at some amplified loci, large numbers of dPET clusters were connected to form complex rearrangement units. In these units, it might be misleading to assign a particular SV type to a dPET cluster; therefore, we introduced the supercluster concept to distinguish isolated from complex SVs in each library [21]. All the SVs involved in superclusters with count 1–3 were classified as isolated events and the rest were classified as complex. MCF-7 had the most complicated rearrangement unit and higher supercluster counts than HCT116 and K562 (Figure S2). The largest rearrangement unit in the MCF-7 10 kb library involved 212 dPET clusters, of which 210 were located in the amplified regions on chromosomes 1, 3, 17, and 20, indicating their inter-connections. The highest supercluster count of the 10 kb library in K562 and HCT116 was 115 and 41, respectively. The lower maximum supercluster count in HCT116 indicated less rearrangement of this genome compared to MCF-7 and K562.

### Comparison of isolated SVs from 1 kb, 10 kb and 20 kb DNA-PET libraries

**Figure 4:**
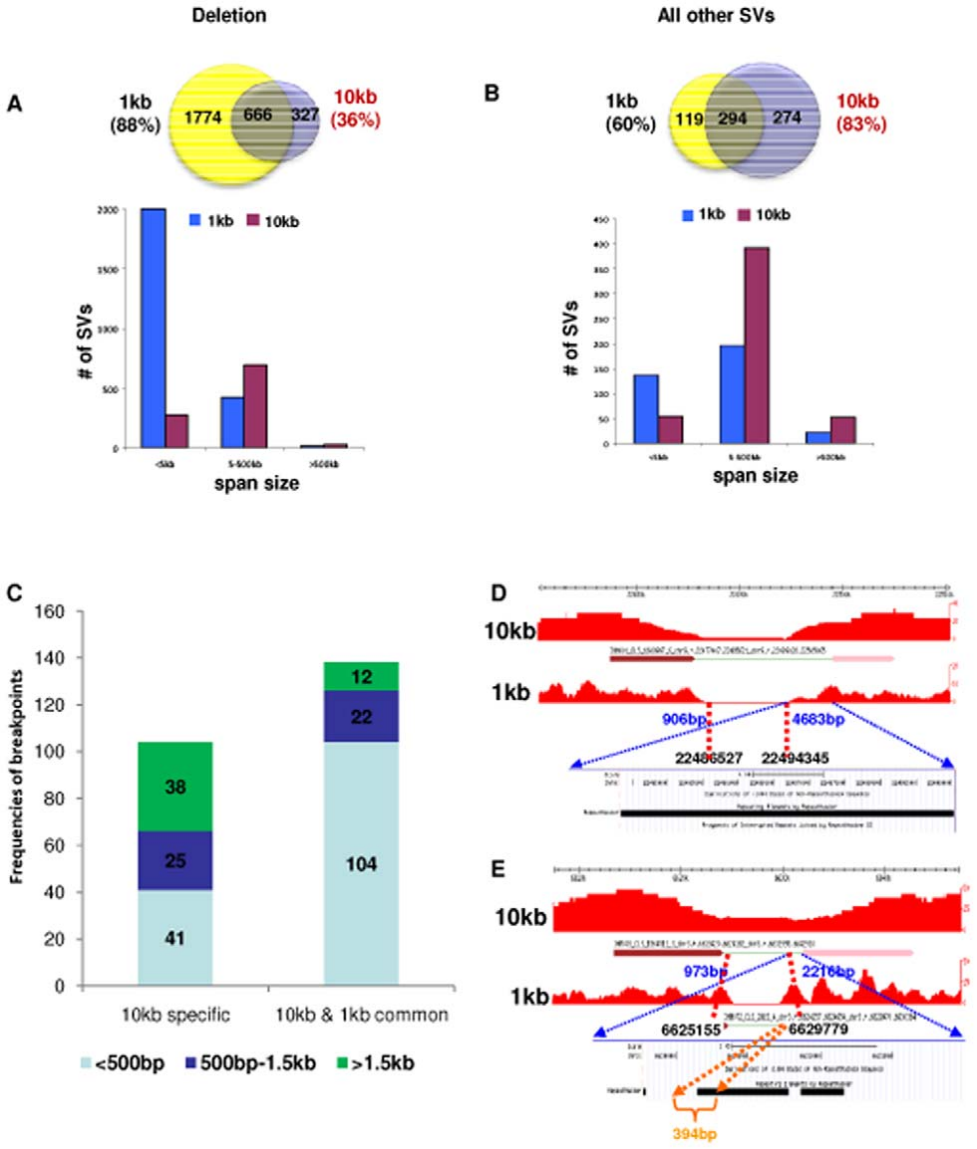
Comparison of SVs identified by 1 kb and 10 kb libraries in the three genomes. (A–B) Number of SVs identified in 1 kb libraries compared to the number of SVs identified in 10 kb libraries. Top, Venn diagrams showing the respective numbers of SVs in each library type and the overlap of SVs. Percentages in parentheses represent SVs identified by 1 kb or 10 kb libraries in all three genomes. Bottom, number of SVs (y-axis) of the indicated SV categories was shown for the specific span sizes (x-axis). (C) Breakpoints confirmed by PCR and Sanger sequencing and their resolution (defined as the distance in bp between the predicted and actual breakpoints). (D) A 10 kb library specific deletion in MCF-7. The genomic PCR and Sanger sequencing confirmed left and right breakpoints are chr9:22,486,527 and chr9:22,494,345, respectively (hg18). The resolution of the left and right sides of the deletion are 906 bp and 4,683 bp, respectively. Repetitive sequence which does not allow unambiguous mapping covers the entire 4,683 bp region. The repetitive sequence could not be spanned by the 1 kb library preventing the identification of this deletion. (E) A deletion in MCF-7 identified by both 10 kb and 1 kb libraries. The left and right breakpoints confirmed by genomic PCR and Sanger sequencing are at chr3:6,625,155 and chr3:6,629,779, respectively (hg18). Based on the 10 kb library predicted breakpoints, the resolution on the left and right sides of the deletion are 973 bp and 2,216 bp, respectively. The tags of the 1 kb library mapped to the gap (in orange) between the repetitive sequences and allowed the identification of this deletion.

To determine the sensitivity of different insert sizes in identifying SVs, we compared SVs detected by different insert size libraries of each genome. We excluded complex SVs from this analysis but included clusters of size 2 for SVs which matched an SV in another size library. For 10 kb and 20 kb library-specific SVs, we increased the cluster count cut off from 3 to 6 to increase the confidence. The results showed that 1 kb libraries could identify more small deletions than 10 kb libraries. Such 1 kb library-specific deletions were usually smaller than 5 kb. The span of most deletions detected by 10 kb libraries ranged between 5 kb and 500 kb (Figure 4A).

Inversions are prone to map to segmental duplications [22]. The ambiguous mapping for sequence tags of segmental duplications results in the exclusion of such tags in most pipelines and the inability to identify breakage/fusion points in these regions. Small insert libraries such as 1 kb libraries are less likely to span such regions of ambiguous mapping and are expected to have lower detection rates for inversions. The lower physical coverage of 1 kb libraries, as compared to 10 kb libraries further limits their ability in identifying inversions. The MCF-7 1 kb library resulted in only 1 inversion with a cluster size ≥3 (Table S4). However, the comparison with the 30 inversions identified by the 10 kb library indicated that 3 inversions matched with at least one low confidence cluster of size 2 in the 1 kb library. Similarly, we identified 33 inversions by the 10 kb library in HCT116 and found evidence by low confidence clusters for only 4 inversions by the respective 1 kb library. Compared to 1 kb libraries of MCF-7 and HCT116, the higher total PET number of the K562 1 kb library resulted in a higher physical coverage and the identification of more inversions. In K562, there were two 1 kb library-specific inversions with a span less than 2 kb. The 10 kb library data of K562 contained one of the two clusters which identify an inversion.

The difference in the detection rate of insertions between 1 kb and 10 kb libraries was comparable to inversions. There was no 1 kb library-specific insertion in MCF-7. In HCT116 and K562, two and one 1 kb library-specific insertions were identified, respectively, and all were smaller than 1 kb. Of these three small insertions, the respective 10 kb libraries identified one of the two breakage/fusion points. In summary, compared to 1 kb libraries, 10 kb libraries had a higher sensitivity in identifying large span SVs. All the SVs which were specific to 1 kb libraries and which were missed by 10 kb libraries had either a low cluster count or a short span (Figure 4B and Figure S3).

The comparison of the ability to identify SVs by the 10 kb and 20 kb libraries of K562 showed a slightly higher detection rate of inversions and unpaired inversions for the 20 kb library and a higher detection rate of deletions for the 10 kb library (Figure S4). The span of the majority of the events from these two libraries was comparable, between 5 kb to 500 kb (Figure S4). One hundred and ninety-four deletions were identified by the 10 kb but not the 20 kb library. The vast majority (188) were short deletions (<20 kb). Fifteen deletions were specific to the 20 kb library and half of them had either both or one anchor region located in a repetitive region which could not be spanned by the 10 kb library (Figure S5). Similarly, tandem duplications which were exclusively identified by the 10 kb library were small in size (<20 kb). The 20 kb library-specific unpaired inversions or inversions which were missed by the 10 kb library showed a low cluster count or had one/both anchors located in repetitive regions.

After the comparison of the SVs from different insert size libraries, we combined the common SVs from different insert size libraries and obtained the genome-specific structural variations (Table S5, S6, S7 and S8). In these three genomes, deletion and tandem duplication were the most abundant SVs whereas the number of inversion and insertion was less than other type of SVs. HCT116 showed the lowest number of complex SVs, suggesting a lower degree of rearrangements compared to MCF-7 and K562.

### Validation of predicted SVs and Resolution of 1 kb and 10 kb libraries

Genomic PCR and consequent Sanger sequencing were used to confirm the breakpoints of 161 randomly selected SVs of the three genomes and a total of 129 SVs (80%) were confirmed (Table S9). Twenty of the 32 SVs which could not be validated had a cluster count <10 indicating low cluster count represented a lower confidence of SV predication. Eight of the non-validated SVs (cluster counts 11 to 50) had shorter anchor spans which suggested that repetitive sequences around the breakpoints inhibited the PCR amplification or mapping error. The remaining two unpaired inversions (cluster counts 156 and 122) and two isolated translocations (cluster counts 113 and 245) were within complex regions with high supercluster count. The intersection of different breakpoints most likely inhibited the PCR amplification. Further, we attempted to validate the 20 kb library-specific events (7 unpaired inversions and 6 inversions) by PCR. Among the 19 dPET clusters, only 7 gave single bands and the capillary sequencing only confirmed two clusters with cluster counts of 53 and 41, respectively. The low validation rate of 20 kb library-specific SVs which all had low cluster counts indicates that a more stringent cluster count cutoff should be applied to 20 kb libraries. Unspecific PCR amplification by repetitive sequences around the breakpoints likely contributed to the low validation rate.

We calculated the breakpoint resolution of 1 kb and 10 kb libraries and defined the resolution as the genomic distance in bp between the dPET clusters predicted breakpoint coordinate and the breakpoint coordinate determined by genomic PCR and Sanger sequencing. Inversions were excluded from the resolution calculation as they tend to be located in repetitive regions which do not allow the unambiguous positioning of the breakpoints. In total, 244 breakpoints were used to calculate the resolutions (242 in 10 kb libraries and 140 in 1 kb libraries) (Table S10). For both 10 kb and 1 kb library, the highest resolution was 0 bp and the lowest resolution for the 10 kb libraries was 10,799 bp and 1,205 bp for the 1 kb libraries. Importantly, the median resolution was 377 bp for 10 kb libraries and 115 bp for 1 kb libraries. This indicated that the higher coverage of the large insert libraries provides a resolution for the majority of breakpoints which is comparable to small insert libraries.

A large distance between the predicted breakpoints by dPET clusters and the true breakpoint locations is indicative of repetitive sequences which prevent a unique mapping. Of the 244 confirmed breakpoints, 104 were identified only by a 10 kb library, 138 were identified by 1 kb and 10 kb libraries, and 2 were identified only by a 1 kb library. Of all the 10 kb library specific breakpoints, 38/104 (36.5%) had a breakpoint resolution larger than 1.5 kb, whereas only 12/138 (8.7%) of 1 kb and 10 kb library common breakpoints had a resolution larger than 1.5 kb (Figure 4C; P<10^−9^ [Fisher Exact Test]). Manual investigation of 10 kb specific breakpoints with resolutions larger than 1.5 kb showed that 33 of the 38 breakpoints (87%) had repetitive sequences covering the distances between predicted and true breakpoints, especially in the 1.5 kb regions next to the confirmed breakpoints (Figure 4D). Only 4 of the 12 (33%) 1 kb and 10 kb common breakpoints were covered by repetitive sequences. The repetitive sequences were discontinuous and tags of the 1 kb dPET mapped to the gaps between the repetitive sequences allowing for the identification of the respective SVs (Figure 4E). This strongly suggested that larger repetitive sequences around breakpoints prevent mapping of tags of 1 kb libraries and hence the identification of the respective SVs.

### Reconstruction of the *BCR-ABL1* amplicon of K562 by fusion point guided concatenation

**Figure 5:**
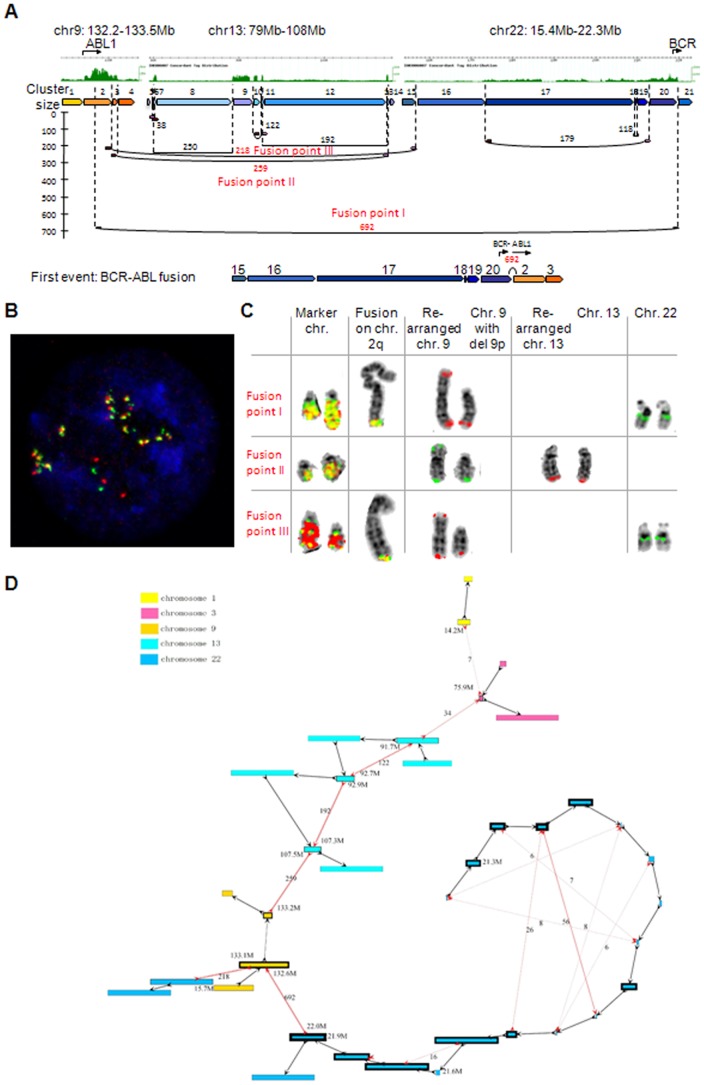
Reconstruction of the *BCR-ABL1* amplicon of K562. (A) Concordant tag distributions representing copy number are shown for amplified genomic regions (top, green track). Genomic segments between predicted breakpoints are indicated by colored arrows and dPET clusters with cluster sizes greater than 35 of predicted somatic rearrangements are represented by horizontal lines flanked by dark red and pink arrows indicating 5′ and 3′ anchor regions (middle). Small to large dPET clusters are arranged from top to bottom. Cluster sizes are indicated. High dPET cluster size of the CML causing *BCR-ABL1* translocation suggests that the rearrangement occurred early and that it has subsequently been amplified. Fusion points I–III correspond to panels C–D. (B) Fluorescence *in situ* hybridization (FISH) of *BCR-ABL1* rearrangement (fusion point I with cluster size 692). Yellow spots represent fusion signals and illustrate the amplification of *BCR-ABL1*. (C) FISH analysis of metaphase chromosomes of three high copy fusion points: I) probes used in B show fusion signals on two marker chromosomes and on chromosome 2q and normal localization on both rearranged chromosomes 9 and normal chromosome 22; the fusion on chromosome 2 has not been identified by DNA-PET most likely due to low sequence complexity at the break point or complex rearrangements, II) probes spanning the fusion point II (cluster size 259) show fusion signals on the same marker chromosomes and normal localization on both normal and rearranged chromosomes 9 and 13, III) probes spanning fusion point III (cluster size 218) show fusion signals on the same marker chromosomes and normal localization on both normal chromosome 22 and rearranged chromosomes 9. (D) Contigs (indicated by boxes) which were covered by PET mapping were concatenated by fusion-point-guided-concatenation method. The length of a contig is represented by the length of the box. Because of the size difference between chromosomes 1, 3, 9, 13, and 22, the length of chromosome 22 is represented by the length of contig/10,000 while the lengths of chromosomes 1, 3, 9, and 13 are represented by the length of contig/100,000. Any value less than 0.1 is rounded to 0.1; any value larger than 6 is rounded to 6. The thickness of borders of each contig represents the coverage (copy number). Red dashed edges represent dPET edges, while black bold edges represent cPET edges. The thickness of dPET edges represents the size of the corresponding dPET cluster. cPET edges have uniform thickness. Arrow heads pointing towards a contig indicate connections with the lower coordinates, arrow heads pointing away from a contig indicate connections with the higher coordinates.

The identification of SVs by paired-end sequencing provides a detailed understanding of local genomic structures. However, cancer genomes frequently show complex rearranged amplifications. To reconstruct these complex rearrangements, a collective analysis of the rearrangement points is required. We therefore employed a fusion-point-guided-concatenation algorithm (see materials and method) to jointly visualize genomic segments surrounding the translocation (chr9/chr22) which creates the CML causing fusion gene *BCR-ABL1* [23] in K562 (Figure 5D). The analysis showed that i) the disease causing rearrangement point had the highest dPET cluster size (692) supporting the concept of amplified rearrangement points as indicators of driver events [21], ii) five different chromosomes (1, 3, 9, 13, and 22) were involved in this amplification, and iii) the core region was located in different genomic contexts indicated by alternative paths at the edges of the amplicon (Figure 5A). To place the amplicon picture in a cytogenetic context, we analyzed the three most amplified rearrangement points (largest dPET clusters) by fluorescence in situ hybridization (FISH) (Figure 5B and C). The FISH analysis confirmed the amplification of the fusion points and showed that the amplicon was distributed over two marker chromosomes. Further, a subpopulation of the BCR-ABL1 amplicon path connecting chromosome 9 at 133.1 Mb with chromosome 22 at 15.7 Mb (dPET cluster count 218) but not the path connecting chromosome 9 at 133.2 Mb with chromosome 13 at 107.5 Mb was located on chromosome 2q.

We also reconstructed the whole genome rearrangement of MCF-7, HCT116 and K562 by this fusion-point-guided-concatenation algorithm using all the dPET clusters with count ≥3 and the detail can be found in Appendix S1, S2 and S3.

## Discussion

The PET sequencing has become a key technique to assess genome rearrangements and SVs in normal and cancer genomes [14], [15], [20], [21], [24], [25], [26]. However, some characteristics are still not well understood regarding study design and the balance of cost versus benefit of different sequencing strategies. One such factor is the choice of the most suitable sequencing library insert size. Using a quantitative study, Bashir *et al.* concluded that larger clones could maximize the clonal coverage and detect as many rearrangement breakpoints as possible while reducing the sequence effort, whereas smaller clones could provide better localization [27]. This conclusion was confirmed by Bentley and McKernan, who observed that when using different insert sizes, most of their predictions were unique to one data set, and the probability of detecting a breakpoint with a combined library was higher than using only one type of library [24], [28]. However, 200 bp to 3 kb insert sizes are not large enough since only lengths of >7 kb are expected to span common transposon insertions such as L1s (the canonical L1 element is 6 kb long) [29] and thereby can identify insertion events in a single read. Using three cancer cell lines, MCF-7, HCT116, and K562 as test genomes, we sequenced different insert size libraries (1 kb, 10 kb, and 20 kb) to identify SVs. The comparison of different insert size libraries demonstrated that the PET sequencing strategy with large insert sizes (10 kb) is an attractive whole-genome sequencing approach to identify SVs in human genomes. With the same sequencing effort, the 10 kb libraries could identify more and larger SVs than 1 kb libraries. This higher sensitivity of large insert libraries is due to the higher physical coverage compared to small insert libraries. Further, the large inserts allow spanning across repetitive sequences which can flank break points. Repetitive sequences result in ambiguous mapping and PETs with tags mapping to repetitive regions are excluded. The 1 kb libraries were advantageous in identifying deletions with span <5 kb. The larger number of deletions identified by 1 kb libraries is due to the more precise insert size selection and thereby smaller standard deviation of the insert size distribution. This results in a higher sensitivity for the identification of small deletions since deletions are identified by a PET mapping distance which is larger than the general insert size distribution of the respective library. However, 10 kb libraries had a comparable resolution in predicting breakpoint locations to a distance that can be amplified by PCR. This is important to note as large insert libraries are often believed to have a proportionally lower breakpoint resolution. The 20 kb insert size library requires a more stringent cluster count cutoff and might have a slight advantage in discovering inversions and unpaired inversions but displayed a lower sensitivity in identifying small SVs of various categories compared to 10 kb insert size library, whereas the construction of libraries with 20 kb inserts requires more genomic DNA as starting material. The detailed characterizations of SVs by large insert size libraries showed many new sub-types of insertions, which could help in understanding the genesis and effect of insertions in human normal and cancer genomes.

This study is complementary to those that have investigated the effect of read-lengths and library-size on the ability to do *de novo* assembly of the data [30], [31], [32]. In a recent work [32], the authors suggest that multiple library sizes are needed to optimally resolve various classes of repeats. While larger library sizes allow the spanning of more repeat classes the associated complexity of assembly analysis also increases. These considerations make the choice of library-size for *de novo* assembly less clear-cut when compared to reference-guided SV analysis.

With the rapid development of next generation sequencing technologies, whole genome sequencing has become an invaluable tool for obtaining a complete understanding of human genomic variation. In the future, personal genomic information will gain importance to tailor an individual's medical care. Our study provides valuable information on the characteristics of PET sequencing libraries and such information will help to select appropriate and most effective insert sizes for various kinds of sequencing projects.

## Materials and Methods

### Cell culture and genomic DNA extraction

MCF-7 (ATCC# HTB-22™), HCT116 (ATCC# CCL-247™) and K562 (ATCC# CCL-243™) were grown under standard culture conditions and harvested at log phase. The genomic DNA was extracted by Blood & Cell Culture DNA Midi Kits (QIAGEN) according to the manufacturer's instruction.

### Library construction and sequencing

We randomly sheared up to 50 µg of genomic DNA to 1 kb, 10 kb and 20 kb fragments by HydroShear (Genomic Solutions Inc) according to the manufacturer's instruction. The fragmented DNA was methylated using EcoP15I (NEB) and end polished by End-It™ DNA End-Repair kit (Epicentre Biotechnologies). SOLiD EcoP15I CAP adaptor (Applied Biosystems) which contains the EcoP15I restriction site was blunt-end ligated to the two ends of DNA fragments and the ligation products were size-selected on an agarose gel. The small DNA fragments (1 kb) were purified by QIAquick Gel Extraction Kit (QIAGEN) and large DNA fragments (10 kb and 20 kb) were purified by QIAEX II Gel Extraction Kit (QIAGEN). Up to 1 µg of gel selected DNA fragments were circularized with the biotinylated SOLiD Internal Adaptor (Applied Biosystems) at extreme diluted concentration (0.1 ng/µl) and the uncircularized DNA fragments were removed by Plasmid-Safe™ ATP-Dependent DNase (Epicentre Biotechnologies). The remaining circularized DNA fragments were digested by EcoP15I (NEB) to release the 25–27 bp di-tags from genomic DNA fragments. Di-tag constructs were end repaired, bound to streptavidin beads and washed. SOLiD sequencing adaptors (Applied Biosystems) were ligated and di-tag constructs were amplified with SOLiD PCR primers (Applied Biosystems) by a 16-cycle PCR. High-throughput sequencing of the 2×25 bp libraries was performed on SOLiD sequencers according to the manufacturer's recommendations (Applied Biosystems). The details of library construction can be found in Protocol S1.

The sequencing data from this study have been submitted to NCBI Gene Expression Omnibus (GEO) (http://www.ncbi.nlm.nih.gov/geo) under accession no. GSE32674 for 1 kb and 20 kb libraries and GSE26954 for 10 kb libraries.

### Random selection of a subset library of MCF-7 and HCT116 10 kb libraries

The sub-sampling of libraries was done to keep the number of non-redundant PETs across all libraries in each genome roughly consistent. Therefore, firstly we removed all redundant PETs based on the 5′ and 3′ tag mapping location, and a random number between (0–1) was generated for each PET. If the number was less than ‘p’, where p = (Required # of PETs)/(Total # of PETs), the PET was included in the randomly down sampled data set.

### PET sequencing analysis

#### PET extraction, classification, clustering

The paired tags designated as R3 and F3 were mapped individually to the reference sequence (hg18, NCBI build 36) in a color space by the ABI SOLiD pipeline Corona Lite (Life Technologies). If both F3 and R3 tags of one bead have matches to the reference with up to 2 mismatches, the pipeline tries all R3/F3 combinations to see if there is a single pair combination which can be reported as AAA indicating that both tags are on the same chromosome, same strand, read in the same direction, are in the correct order and within correct distance of each other (3 kb for 1 kb library, 20 kb for 10 kb library and 40 kb for 20 kb library, respectively). Based on ABI SOLiD pairing report, we further separated all the PETs into concordant PETs (cPETs) and discordant PETs (dPETs). cPETs were defined as those PETs where both tags mapped to same chromosome, same strand, in the correct 5′ to 3′ ordering and within expected span range. The PETs which were rejected by cPET criteria were classified as dPETs.

To filter out those chimeric dPETs due to ligation error in the library construction process, dPET which span the same fusion point were required to form clusters. The number of the dPETs clustering together around a fusion point was represented by the cluster size or cluster count. The genomic region which was covered by the 5′ tags of a cluster was defined as the 5′ anchor and the genomic region which was covered by the 3′ tags of a cluster was defined as the 3′ anchor.

#### SVs identification

SVs with one rearrangement point could be identified by single dPET clusters, such as deletions if the 5′ mapping anchor region was far away from the 3′ mapping anchor region, tandem duplications if the mapping order was 3′ to 5′ instead of the normal 5′ to 3′, unpaired inversion if the mapping orientation was revered (on different strand), and isolated translocations if the 5′ and 3′ anchors mapped to different chromosomes. Inversions, insertions and balanced translocations were identified by two closely positioned dPET clusters.

#### Superclustering

To separate breakpoints in complex regions from isolated and less complex SVs, a breakpoint based interconnection network was established. The extension from the start and end points of each dPET cluster anchor region by the maximum insert size of the library was created as search windows to determine the neighborhood of a breakpoint. The dPET clusters were grouped as a supercluster when windows of neighboring clusters overlapped with each other. The number of dPET clusters that could be joined together into a supercluster was represented by supercluster size or supercluster count. The details of PET sequencing analysis were described in [21]

### Comparison of libraries with different insert sizes

The comparison of dPET clusters across different insert size libraries was performed based on an overlap of the 5′ and 3′ anchor region extended by the individual library insert size. We started from the 10 kb library in MCF-7 and HCT116 and the 20 kb library in K562. For any given 10 kb or 20 kb isolated dPET cluster (supercluster count ≤3), the 5′ and 3′ anchor regions of the cluster was extended by the maximum length of the library towards the breakpoints to create a search window. If the 5′ and 3′ anchor regions of a dPET cluster from other insert size libraries which belonged to the same SV type fell into the search window, the clusters would be grouped as a common SV. If no other cluster could be found in the search window, the cluster would be categorized as a SV specific to that insert size library.

### Breakpoint confirmation by genomic PCR and Sanger sequencing

We validated a subset of breakpoints detected in these three cell lines using genomic PCR. Primers were designed to span the breakpoint predicted by dPET clusters using repeat–masked human genome assembly (NCBI Build 36). The maximum PCR product was 10 kb. PCR was carried out with JumpStart™ REDAccuTaq LA DNA Ploymerase (Sigma-Aldrich Inc., St.Louis, MO) in a 50 µl reaction volume and with 20 ng of genomic DNA as the template. The following program was used: 1) Initial denaturation at 96°C for 30 sec, 2) 15 cycles of 15 sec at 94°C, 30 sec at 58°C, 10 min at 68°C, 3) 25 cycles of 15 sec at 94°C, 30 sec at 55°C, 10 min at 68°C, 4) 68°C for 20 min. Fragments up to 10 kb in size were visualized by agarose gel electrophoresis. PCR products with single band at the expected size range were purified by QIAquick PCR Purification Kit (QIAGEN), sequenced by conventional Sanger capillary methods and the resulting sequences were aligned to the reference sequence to identify breakpoints. We then compared breakpoint coordinates from Sanger sequencing with the breakpoint coordinates predicted by dPET clusters and determined a median resolution for each library size.

### Fluorescence in situ hybridization (FISH)

FISH was performed as described by Inaki [33]. To assess the three high copy fusion points in K562, the following probes spanning the fusion points have been hybridized on K562 cell line:

Cluster size 692 BAC#

Red   RP11-83J21 (chr9:132,641,829–132,818,294)

Green   RP11-61N10 (chr22:21,728,292–21,917,898)

Cluster size 259 BAC#

Red   RP11-83J21 (chr13:107,393,170–107,577,262)

Green   RP11-10619 (chr9:133,087,269–133,237,519)

Cluster size 218 BAC#

Red   RP11-544A12 (chr9:132,955,072–133,152,093)

Green   RP11-104F9 (chr22:15,547,686–15,730,740)

### Reconstruction of genome structure by fusion point guided concatenation

Segmenting of the reference genome into contigs was done on the basis of breakpoints identified by dPET clusters and by identifying additional breakpoints with no physical cPET coverage. Contigs consecutive on the reference genome were then connected by a reference edge in the presence of connecting cPETs. Correspondingly, contigs linked by dPET clusters were represented by dPET edges where the edges were weighted by the size of the cluster. Locally amplified regions were then identified in the following way: Firstly, the dPET edge with the highest weight was selected and the adjacent contigs to this edge were added to the amplicon graph. Then, for each contig in the graph, its neighbors were also added using both reference and dPET links as long as the neighbors were considered amplified (cPET estimated copy-number greater than 2). An amplicon graph was grown until no more contigs could be added in this fashion. The process was then repeated on the unused dPET edges, till none remained, resulting in a set of local amplicon graphs and only graphs with more than two contigs were considered further.

## Supporting Information

Table S1 **Library statistics.**
(XLS)

Table S2 **dPET cluster statistics.**
(XLS)

Table S3 **Sub-types of insertions in individual libraries.**
(XLS)

Table S4 **SVs identified in individual libraries.**
(XLS)

Table S5 **SVs identified in each genome.**
(XLS)

Table S6 **MCF-7 structural variations.**
(XLS)

Table S7 **HCT116 structural variations.**
(XLS)

Table S8 **K562 structural variations.**
(XLS)

Table S9 **Genomic PCR and Sanger sequencing validation statistics.**
(XLS)

Table S10 **Breakpoint resolution of 1 kb and 10 kb libraries.**
(XLS)

Figure S1 **dPET cluster size distribution.** The number of dPET clusters (y-axis) is shown for the individual cluster sizes (x-axis). Red vertical line represents the cutoff for dPET clusters regarded as reliable breakpoint pairs (count three and higher).
(PDF)

Figure S2 **Supercluster statistics in each library.** (a–g) Distribution of degrees of connectivity represented by superclusters in each library. Numbers of clusters (y-axis) for each supercluster count (number of interconnected clusters, x-axis) is shown. (h) Color code of each kind of SV.
(PDF)

Figure S3 **Comparison of number and span distribution of specific SVs identified by 1 kb and 10 kb libraries in MCF-7, HCT116, and K562.** Venn diagrams showing the respective numbers of SVs in each library type and the overlap of SVs. Number of SVs (*y-axis*) of the indicated SV categories (a–d) were shown for the different span sizes (*x-axis*).
(PDF)

Figure S4 **Comparison of number and span distribution of specific SVs identified by 10 kb and 20 kb libraries in K562.** Venn diagrams showing the respective numbers of SVs in each library type and the overlap of SVs. Number of SVs (*y-axis*) of the indicated SV categories (a–e) were shown for the different span sizes (*x-axis*).
(PDF)

Figure S5 **Example of a 20 kb library specific deletion in K562.** (a) The drop of expected coverage (red track) in 10 kb and 20 kb library indicates the presence of a deletion. (b) The deletion only could be detected by a 20 kb library dPET cluster (cluster size 31) and the 5′ and 3′ anchor span were 16,584 bp and 9,921 bp. The red and pink arrows represented the 5′ and 3′ anchor regions of the dPET cluster. (c) The expected anchor span of 20 kb library is 20 kb and the segmental duplications (orange blocks) located at this deletion created the shorter anchor span in 20 kb library. The PETs of 10 kb library could not cross the segment duplications resulting in the failure to detect this deletion by the 10 kb library.
(PDF)

Protocol S1 **Long Span DNA-PET Library Construction by EcoP15I.**
(DOC)

Appendix S1 **Reconstruction of MCF-7 genome structure by fusion point guided concatenation method.**
(ZIP)

Appendix S2 **Reconstruction of HCT116 genome structure by fusion point guided concatenation method.**
(ZIP)

Appendix S3 **Reconstruction of K562 genome structure by fusion point guided concatenation method.**
(ZIP)

## References

[1] Shastry BS (2007) SNPs in disease gene mapping, medicinal drug development and evolution. J Hum Genet 52: 871–880. 17928948 10.1007/s10038-007-0200-z

[2] Cai WW, Mao JH, Chow CW, Damani S, Balmain A, et al (2002) Genome-wide detection of chromosomal imbalances in tumors using BAC microarrays. Nat Biotechnol 20: 393–396. 11923847 10.1038/nbt0402-393

[3] Pinkel D, Segraves R, Sudar D, Clark S, Poole I, et al (1998) High resolution analysis of DNA copy number variation using comparative genomic hybridization to microarrays. Nat Genet 20: 207–211. 9771718 10.1038/2524

[4] Dunn JJ, McCorkle SR, Praissman LA, Hind G, Van Der Lelie D, et al (2002) Genomic signature tags (GSTs): a system for profiling genomic DNA. Genome Res 12: 1756–1765. 12421763 10.1101/gr.306102 PMC187557

[5] Wang TL, Maierhofer C, Speicher MR, Lengauer C, Vogelstein B, et al (2002) Digital karyotyping. Proc Natl Acad Sci U S A 99: 16156–16161. 12461184 10.1073/pnas.202610899 PMC138581

[6] Kim TH, Barrera LO, Zheng M, Qu C, Singer MA, et al (2005) A high-resolution map of active promoters in the human genome. Nature 436: 876–880. 15988478 10.1038/nature03877 PMC1895599

[7] Tuzun E, Sharp AJ, Bailey JA, Kaul R, Morrison VA, et al (2005) Fine-scale structural variation of the human genome. Nat Genet 37: 727–732. 15895083 10.1038/ng1562

[8] Kidd JM, Cheng Z, Graves T, Fulton B, Wilson RK, et al (2008) Haplotype sorting using human fosmid clone end-sequence pairs. Genome Res 18: 2016–2023. 18836033 10.1101/gr.081786.108 PMC2593576

[9] Korbel JO, Urban AE, Affourtit JP, Godwin B, Grubert F, et al (2007) Paired-end mapping reveals extensive structural variation in the human genome. Science 318: 420–426. 17901297 10.1126/science.1149504 PMC2674581

[10] Campbell PJ, Stephens PJ, Pleasance ED, O'Meara S, Li H, et al (2008) Identification of somatically acquired rearrangements in cancer using genome-wide massively parallel paired-end sequencing. Nat Genet 40: 722–729. 18438408 10.1038/ng.128 PMC2705838

[11] Shah SP, Morin RD, Khattra J, Prentice L, Pugh T, et al (2009) Mutational evolution in a lobular breast tumour profiled at single nucleotide resolution. Nature 461: 809–813. 19812674 10.1038/nature08489

[12] Totoki Y, Tatsuno K, Yamamoto S, Arai Y, Hosoda F, et al (2011) High-resolution characterization of a hepatocellular carcinoma genome. Nat Genet 43: 464–469. 21499249 10.1038/ng.804

[13] Berger MF, Lawrence MS, Demichelis F, Drier Y, Cibulskis K, et al (2011) The genomic complexity of primary human prostate cancer. Nature 470: 214–220. 21307934 10.1038/nature09744 PMC3075885

[14] Lee W, Jiang Z, Liu J, Haverty PM, Guan Y, et al (2010) The mutation spectrum revealed by paired genome sequences from a lung cancer patient. Nature 465: 473–477. 20505728 10.1038/nature09004

[15] Stephens PJ, McBride DJ, Lin ML, Varela I, Pleasance ED, et al (2009) Complex landscapes of somatic rearrangement in human breast cancer genomes. Nature 462: 1005–1010. 20033038 10.1038/nature08645 PMC3398135

[16] Stephens PJ, Greenman CD, Fu B, Yang F, Bignell GR, et al (2011) Massive genomic rearrangement acquired in a single catastrophic event during cancer development. Cell 144: 27–40. 21215367 10.1016/j.cell.2010.11.055 PMC3065307

[17] Campbell PJ, Yachida S, Mudie LJ, Stephens PJ, Pleasance ED, et al (2011) The patterns and dynamics of genomic instability in metastatic pancreatic cancer. Nature 467: 1109–1113. 10.1038/nature09460 PMC3137369 20981101

[18] Chapman MA, Lawrence MS, Keats JJ, Cibulskis K, Sougnez C, et al (2011) Initial genome sequencing and analysis of multiple myeloma. Nature 471: 467–472. 21430775 10.1038/nature09837 PMC3560292

[19] Pleasance ED, Cheetham RK, Stephens PJ, McBride DJ, Humphray SJ, et al (2010) A comprehensive catalogue of somatic mutations from a human cancer genome. Nature 463: 191–196. 20016485 10.1038/nature08658 PMC3145108

[20] Pleasance ED, Stephens PJ, O'Meara S, McBride DJ, Meynert A, et al (2010) A small-cell lung cancer genome with complex signatures of tobacco exposure. Nature 463: 184–190. 20016488 10.1038/nature08629 PMC2880489

[21] Hillmer AM, Yao F, Inaki K, Lee WH, Ariyaratne PN, et al (2011) Comprehensive long-span paired-end-tag mapping reveals characteristic patterns of structural variations in epithelial cancer genomes. Genome Res 21: 665–675. 21467267 10.1101/gr.113555.110 PMC3083083

[22] Stefansson H, Helgason A, Thorleifsson G, Steinthorsdottir V, Masson G, et al (2005) A common inversion under selection in Europeans. Nat Genet 37: 129–137. 15654335 10.1038/ng1508

[23] Groffen J, Stephenson JR, Heisterkamp N, de Klein A, Bartram CR, et al (1984) Philadelphia chromosomal breakpoints are clustered within a limited region, bcr, on chromosome 22. Cell 36: 93–99. 6319012 10.1016/0092-8674(84)90077-1

[24] McKernan KJ, Peckham HE, Costa GL, McLaughlin SF, Fu Y, et al (2009) Sequence and structural variation in a human genome uncovered by short-read, massively parallel ligation sequencing using two-base encoding. Genome Res 19: 1527–1541. 19546169 10.1101/gr.091868.109 PMC2752135

[25] Fujimoto A, Nakagawa H, Hosono N, Nakano K, Abe T, et al (2010) Whole-genome sequencing and comprehensive variant analysis of a Japanese individual using massively parallel sequencing. Nat Genet 42: 931–936. 20972442 10.1038/ng.691

[26] Clark MJ, Homer N, O'Connor BD, Chen Z, Eskin A, et al (2010) U87MG decoded: the genomic sequence of a cytogenetically aberrant human cancer cell line. PLoS Genet 6: e1000832. 20126413 10.1371/journal.pgen.1000832 PMC2813426

[27] Bashir A, Volik S, Collins C, Bafna V, Raphael BJ (2008) Evaluation of paired-end sequencing strategies for detection of genome rearrangements in cancer. PLoS Comput Biol 4: e1000051. 18404202 10.1371/journal.pcbi.1000051 PMC2278375

[28] Bentley DR, Balasubramanian S, Swerdlow HP, Smith GP, Milton J, et al (2008) Accurate whole human genome sequencing using reversible terminator chemistry. Nature 456: 53–59. 18987734 10.1038/nature07517 PMC2581791

[29] Cordaux R, Batzer MA (2009) The impact of retrotransposons on human genome evolution. Nat Rev Genet 10: 691–703. 19763152 10.1038/nrg2640 PMC2884099

[30] Chaisson MJ, Brinza D, Pevzner PA (2009) De novo fragment assembly with short mate-paired reads: Does the read length matter? Genome Res 19: 336–346. 19056694 10.1101/gr.079053.108 PMC2652199

[31] Nagarajan N, Pop M (2009) Parametric complexity of sequence assembly: theory and applications to next generation sequencing. J Comput Biol 16: 897–908. 19580519 10.1089/cmb.2009.0005

[32] Wetzel J, Kingsford C, Pop M (2011) Assessing the benefits of using mate-pairs to resolve repeats in de novo short-read prokaryotic assemblies. BMC Bioinformatics 12: 95. 21486487 10.1186/1471-2105-12-95 PMC3103447

[33] Inaki K, Hillmer AM, Ukil L, Yao F, Woo XY, et al (2011) Transcriptional consequences of genomic structural aberrations in breast cancer. Genome Res 21: 676–687. 21467264 10.1101/gr.113225.110 PMC3083084

