# Supplementary figures and images for: Long Span DNA Paired-End-Tag (DNA-PET) Sequencing Strategy for the Interrogation of Genomic Structural Mutations and Fusion-Point-Guided Reconstruction of Amplicons (part 1 of 2)
Source: PLoS One. 2012 Sep 28;7(9):e46152. doi: 10.1371/journal.pone.0046152 (PMC3461012; doi:10.1371/journal.pone.0046152)

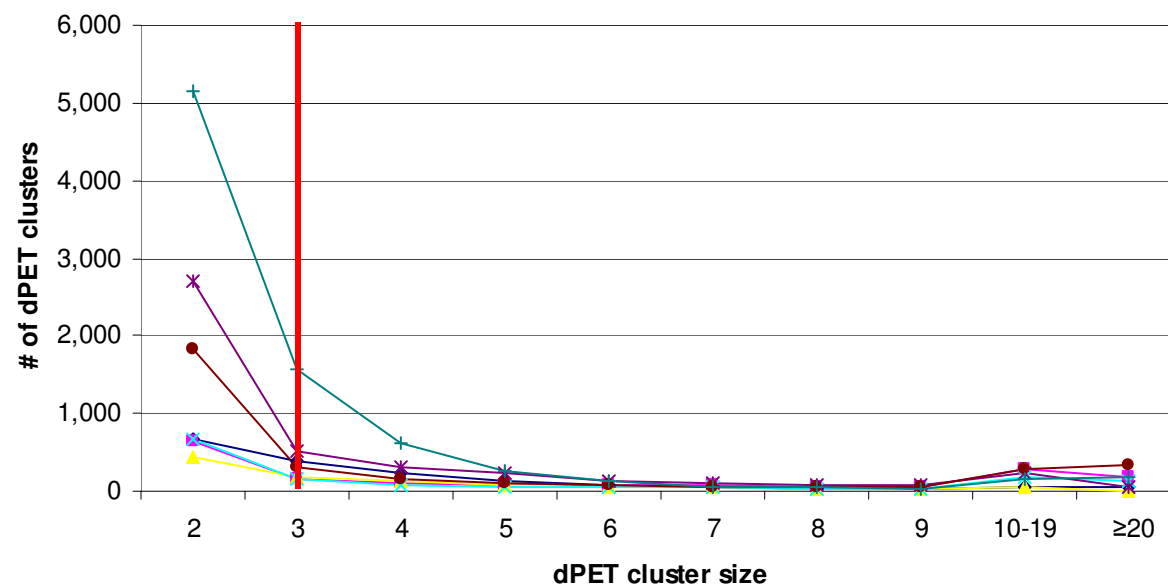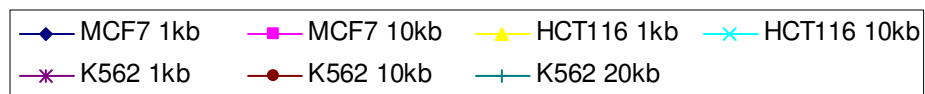

Supplement: Figure S1 — dPET cluster size distribution. The number of dPET clusters (y-axis) is shown for the individual cluster sizes (x-axis). Red vertical line represents the cutoff for dPET clusters regarded as reliable breakpoint pairs (count three and higher). (PDF) [file pone.0046152.s011.pdf]

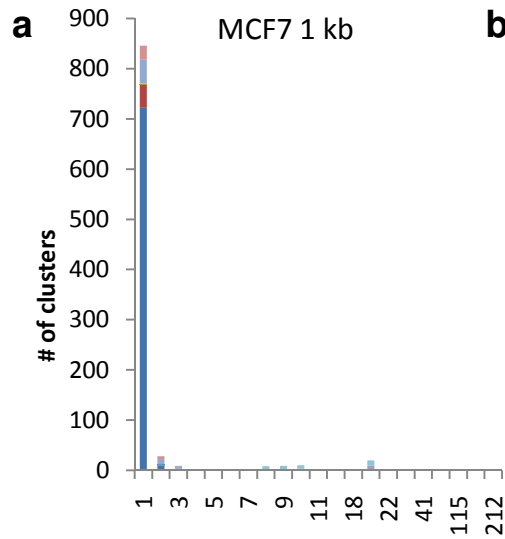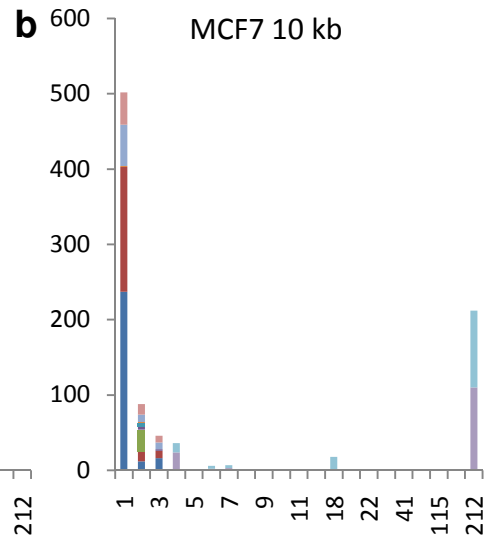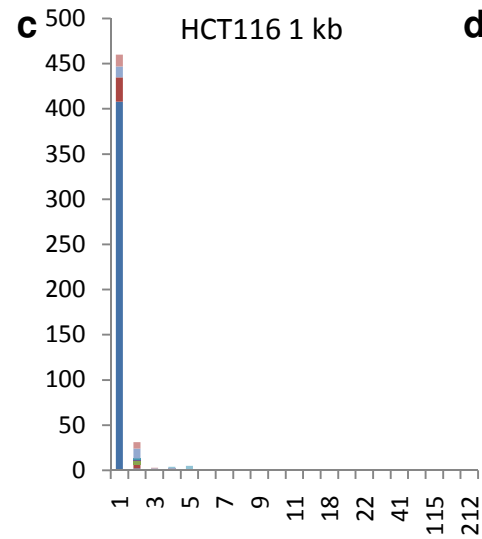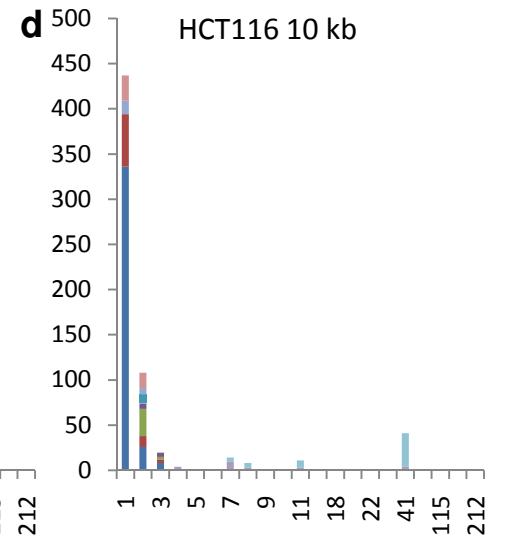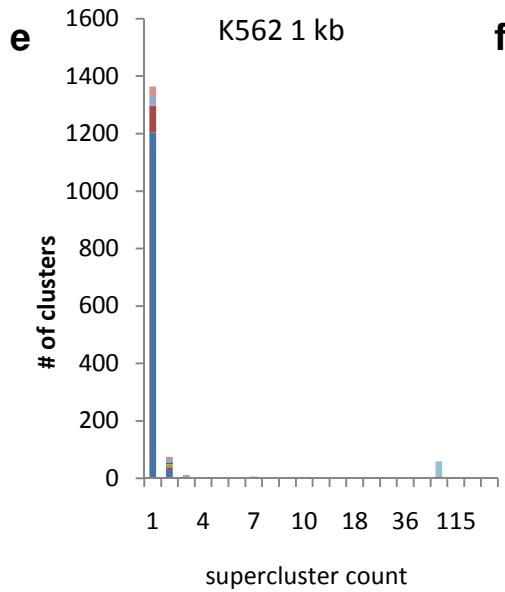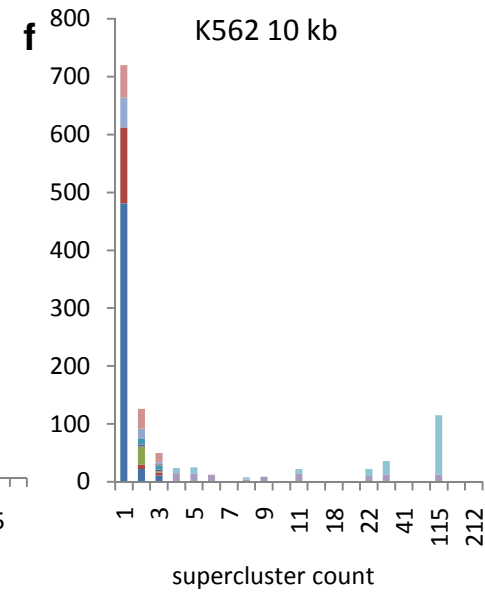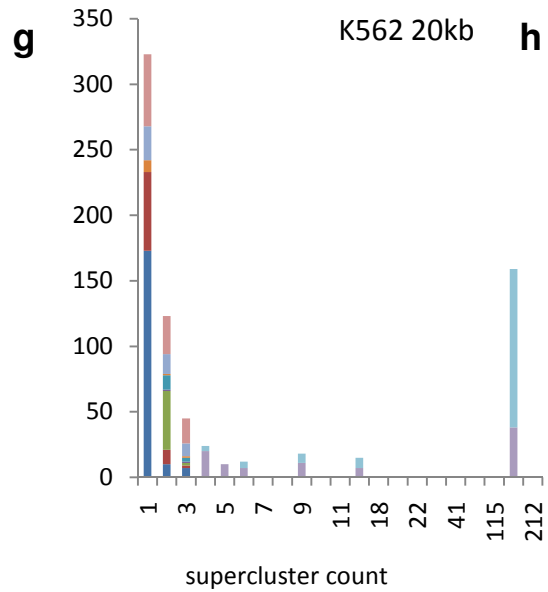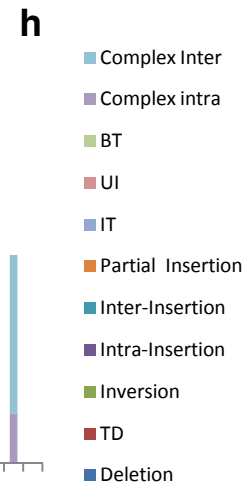

Supplement: Figure S2 — Supercluster statistics in each library. (a–g) Distribution of degrees of connectivity represented by superclusters in each library. Numbers of clusters (y-axis) for each supercluster count (number of interconnected clusters, x-axis) is shown. (h) Color code of each kind of SV. (PDF) [file pone.0046152.s012.pdf]

**a** Tandem duplication

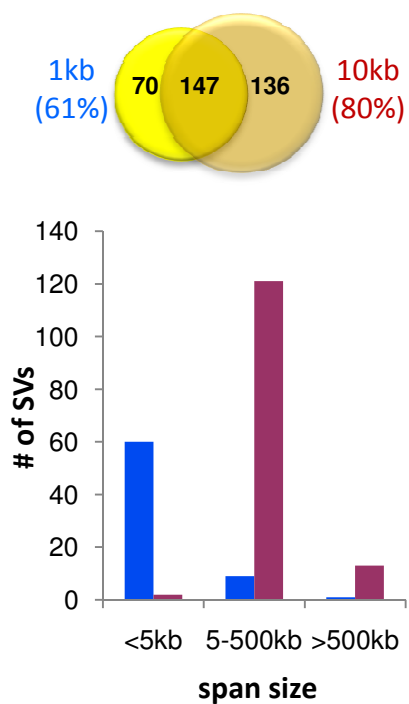

**b** Unpaired inversion

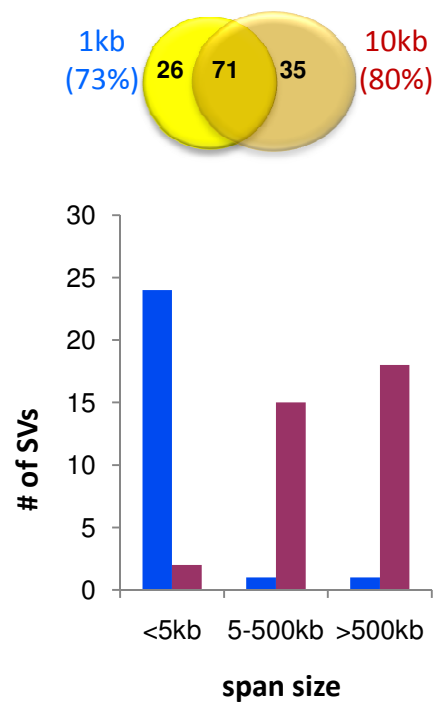

**c** Inversion

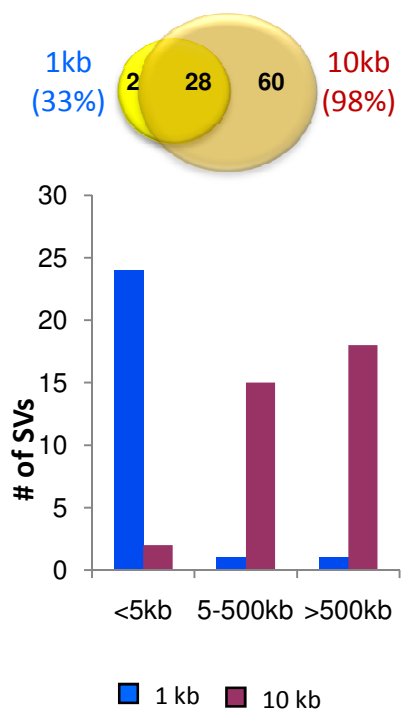

**d** Insertion

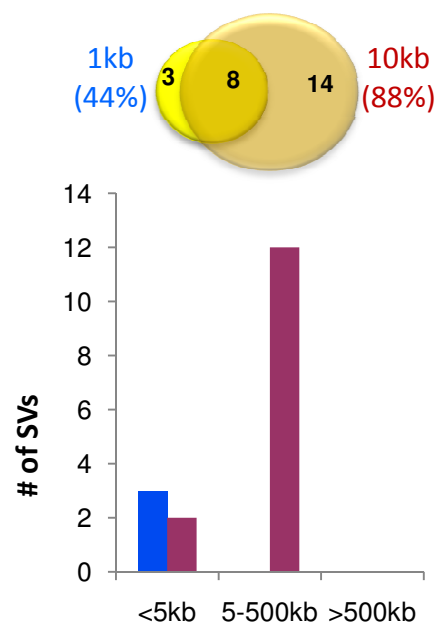

Supplement: Figure S3 — Comparison of number and span distribution of specific SVs identified by 1 kb and 10 kb libraries in MCF-7, HCT116, and K562. Venn diagrams showing the respective numbers of SVs in each library type and the overlap of SVs. Number of SVs (y-axis) of the indicated SV categories (a–d) were shown for the different span sizes (x-axis). (PDF) [file pone.0046152.s013.pdf]

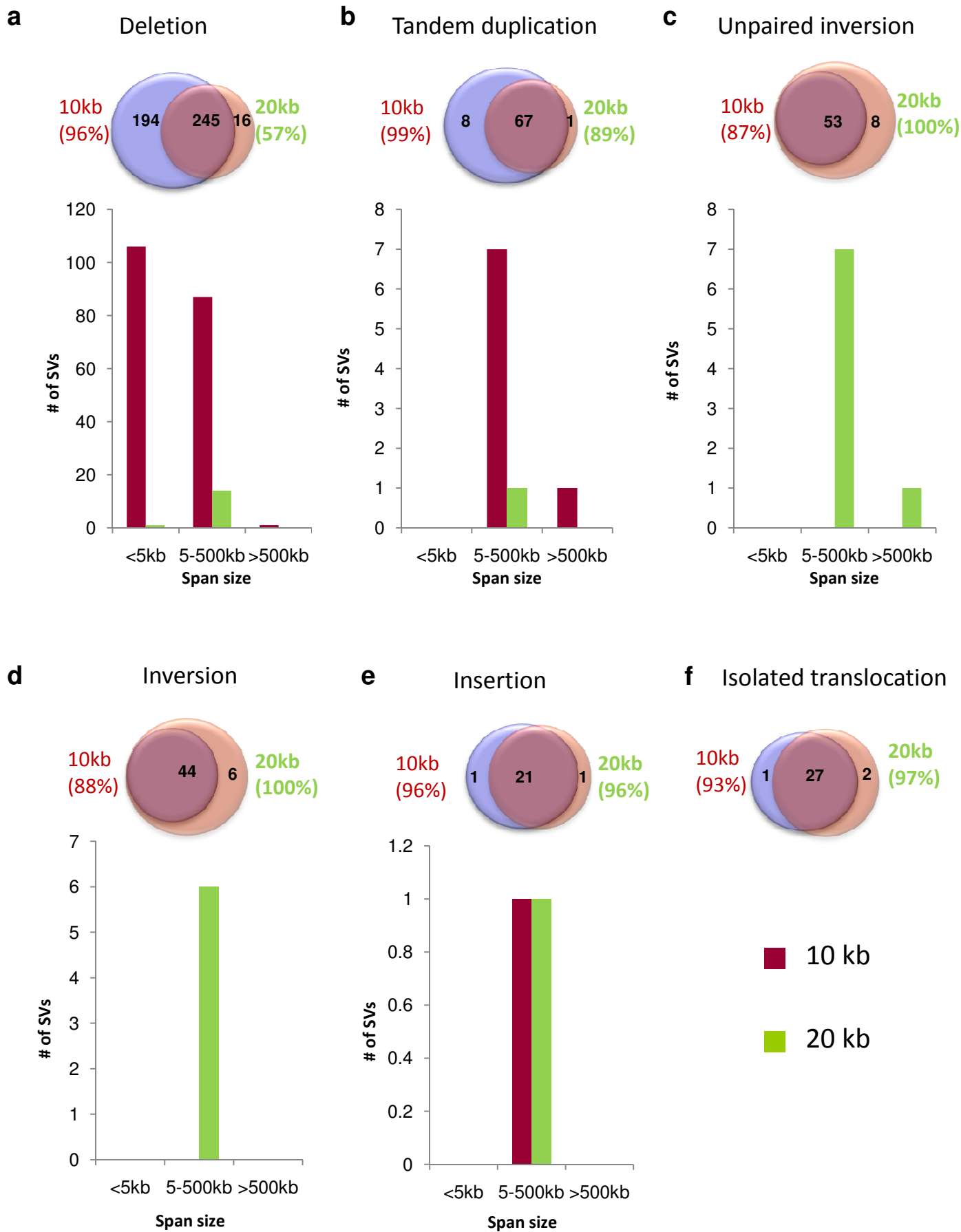

Supplement: Figure S4 — Comparison of number and span distribution of specific SVs identified by 10 kb and 20 kb libraries in K562. Venn diagrams showing the respective numbers of SVs in each library type and the overlap of SVs. Number of SVs (y-axis) of the indicated SV categories (a–e) were shown for the different span sizes (x-axis). (PDF) [file pone.0046152.s014.pdf]

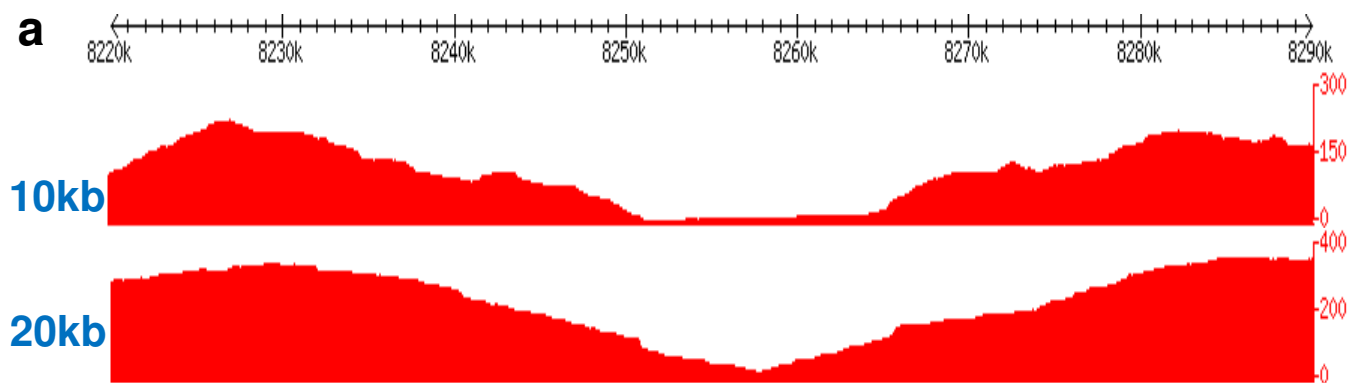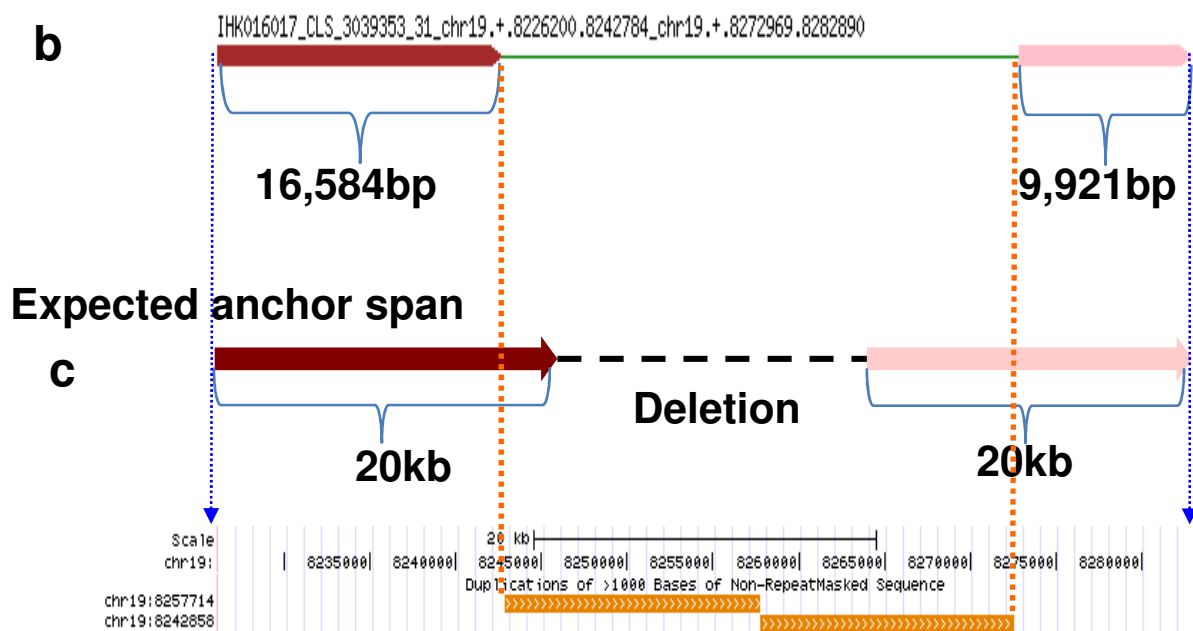

Supplement: Figure S5 — Example of a 20 kb library specific deletion in K562. (a) The drop of expected coverage (red track) in 10 kb and 20 kb library indicates the presence of a deletion. (b) The deletion only could be detected by a 20 kb library dPET cluster (cluster size 31) and the 5′ and 3′ anchor span were 16,584 bp and 9,921 bp. The red and pink arrows represented the 5′ and 3′ anchor regions of the dPET cluster. (c) The expected anchor span of 20 kb library is 20 kb and the segmental duplications (orange blocks) located at this deletion created the shorter anchor span in 20 kb library. The PETs of 10 kb library could not cross the segment duplications resulting in the failure to detect this deletion by the 10 kb library. (PDF) [file pone.0046152.s015.pdf]

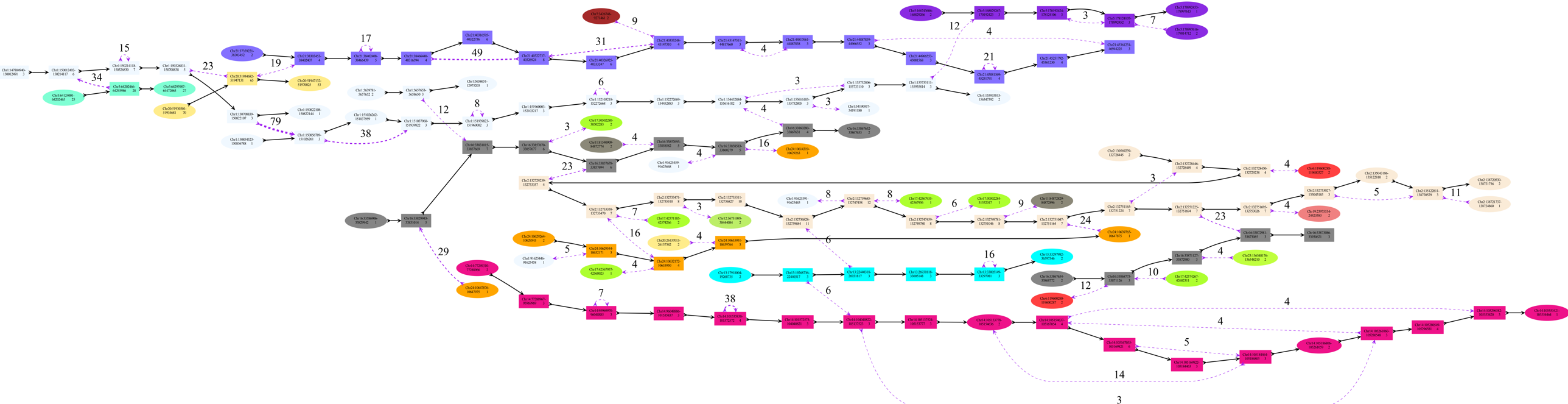

Supplement: Appendix S1 — Reconstruction of MCF-7 genome structure by fusion point guided concatenation method. (ZIP) [file pone.0046152.s017.zip › subgraph9.pdf]

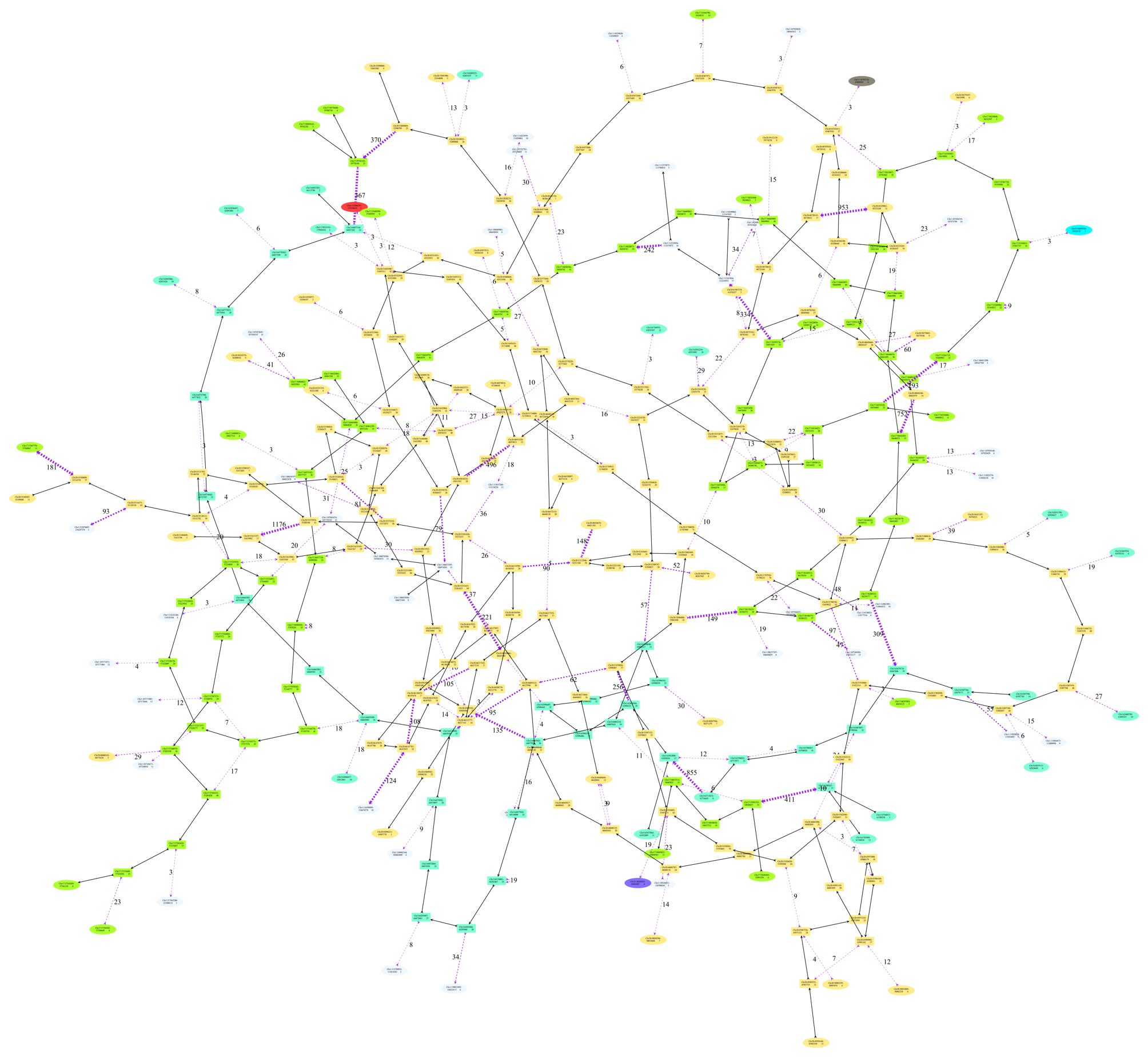

Supplement: Appendix S1 — Reconstruction of MCF-7 genome structure by fusion point guided concatenation method. (ZIP) [file pone.0046152.s017.zip › subgraph1.pdf]

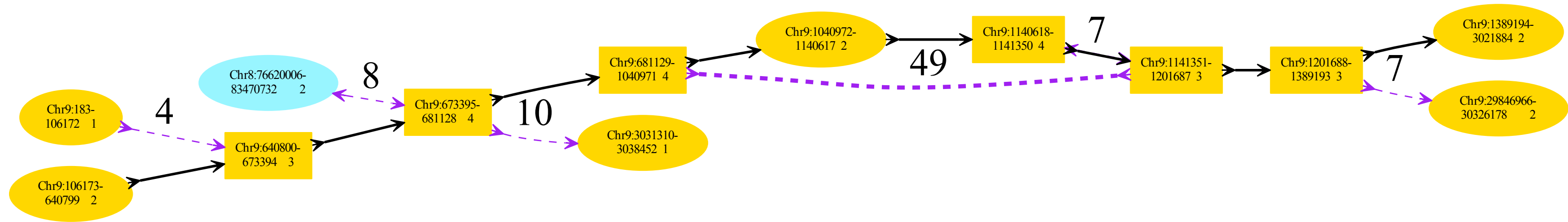

Supplement: Appendix S1 — Reconstruction of MCF-7 genome structure by fusion point guided concatenation method. (ZIP) [file pone.0046152.s017.zip › subgraph10.pdf]

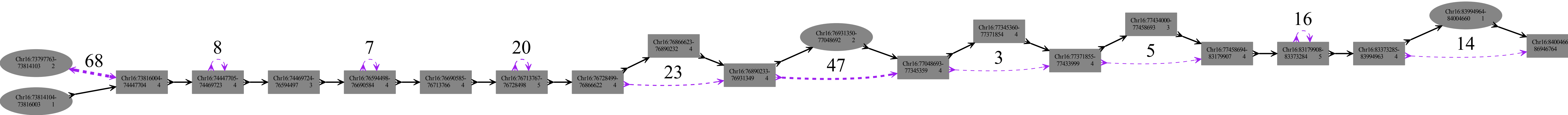

Supplement: Appendix S1 — Reconstruction of MCF-7 genome structure by fusion point guided concatenation method. (ZIP) [file pone.0046152.s017.zip › subgraph11.pdf]

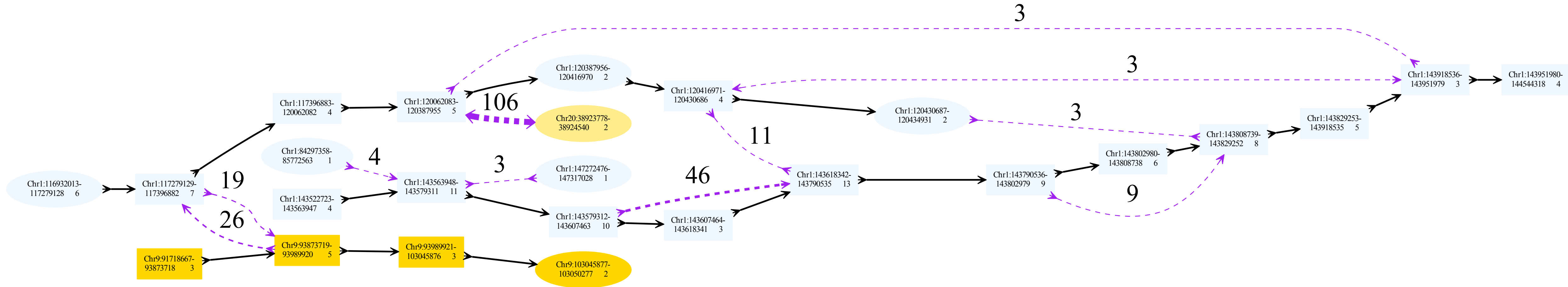

Supplement: Appendix S1 — Reconstruction of MCF-7 genome structure by fusion point guided concatenation method. (ZIP) [file pone.0046152.s017.zip › subgraph12.pdf]

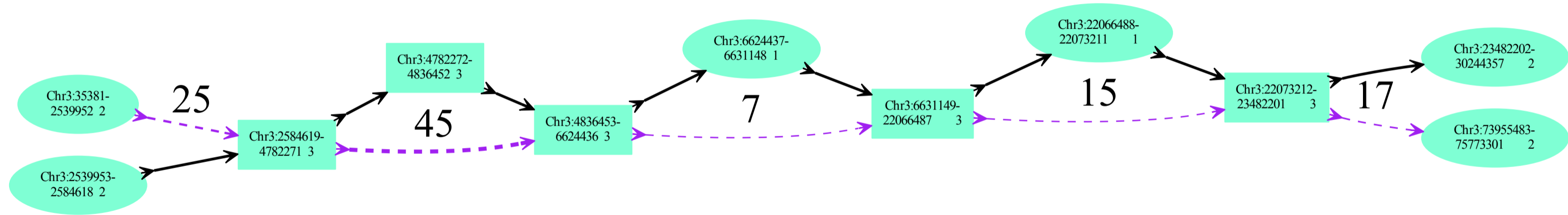

Supplement: Appendix S1 — Reconstruction of MCF-7 genome structure by fusion point guided concatenation method. (ZIP) [file pone.0046152.s017.zip › subgraph13.pdf]

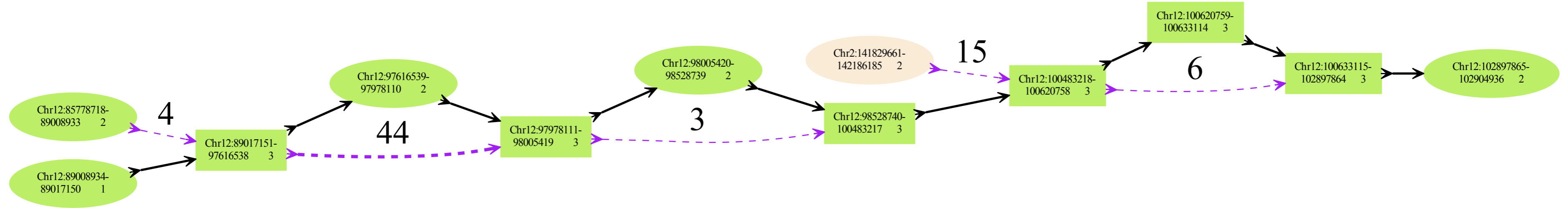

Supplement: Appendix S1 — Reconstruction of MCF-7 genome structure by fusion point guided concatenation method. (ZIP) [file pone.0046152.s017.zip › subgraph14.pdf]

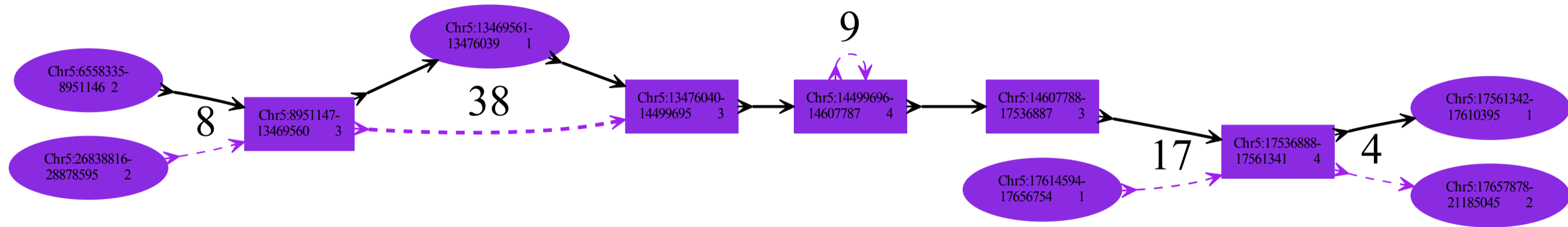

Supplement: Appendix S1 — Reconstruction of MCF-7 genome structure by fusion point guided concatenation method. (ZIP) [file pone.0046152.s017.zip › subgraph15.pdf]

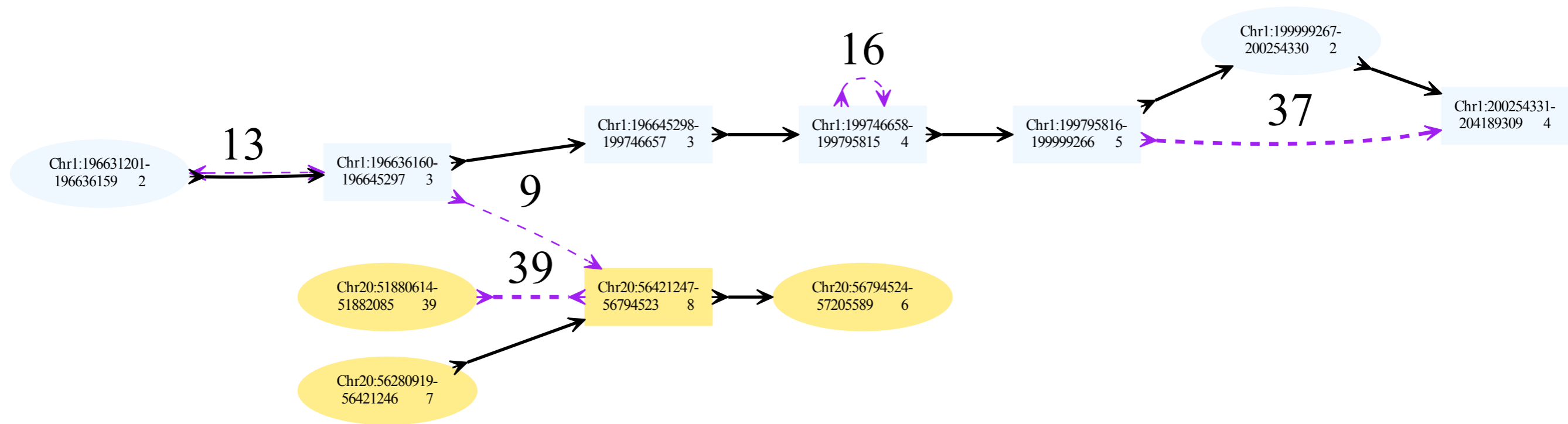

Supplement: Appendix S1 — Reconstruction of MCF-7 genome structure by fusion point guided concatenation method. (ZIP) [file pone.0046152.s017.zip › subgraph16.pdf]

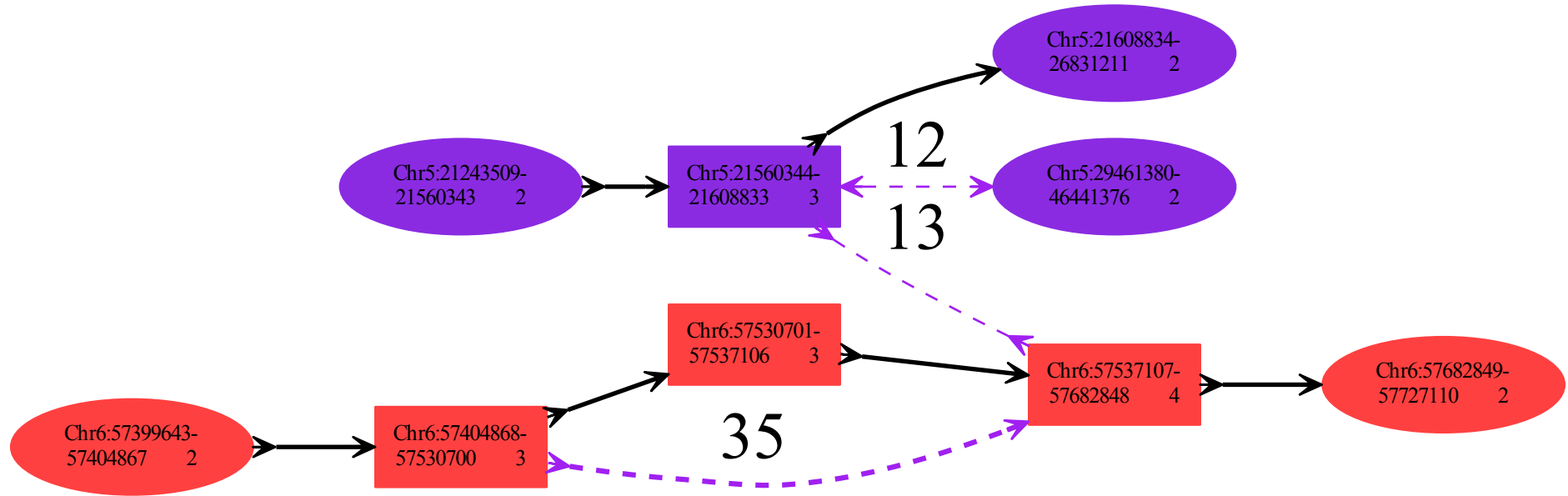

Supplement: Appendix S1 — Reconstruction of MCF-7 genome structure by fusion point guided concatenation method. (ZIP) [file pone.0046152.s017.zip › subgraph17.pdf]

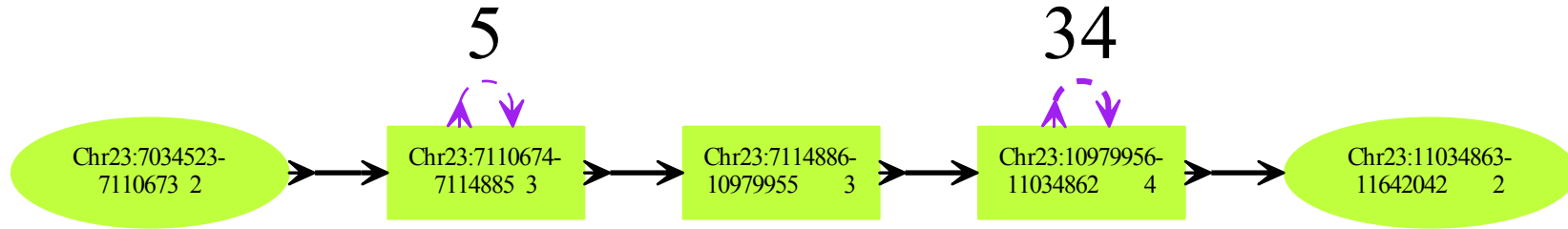

Supplement: Appendix S1 — Reconstruction of MCF-7 genome structure by fusion point guided concatenation method. (ZIP) [file pone.0046152.s017.zip › subgraph18.pdf]

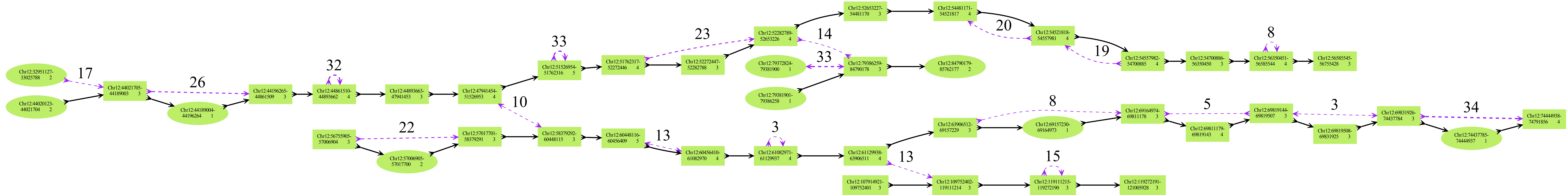

Supplement: Appendix S1 — Reconstruction of MCF-7 genome structure by fusion point guided concatenation method. (ZIP) [file pone.0046152.s017.zip › subgraph19.pdf]

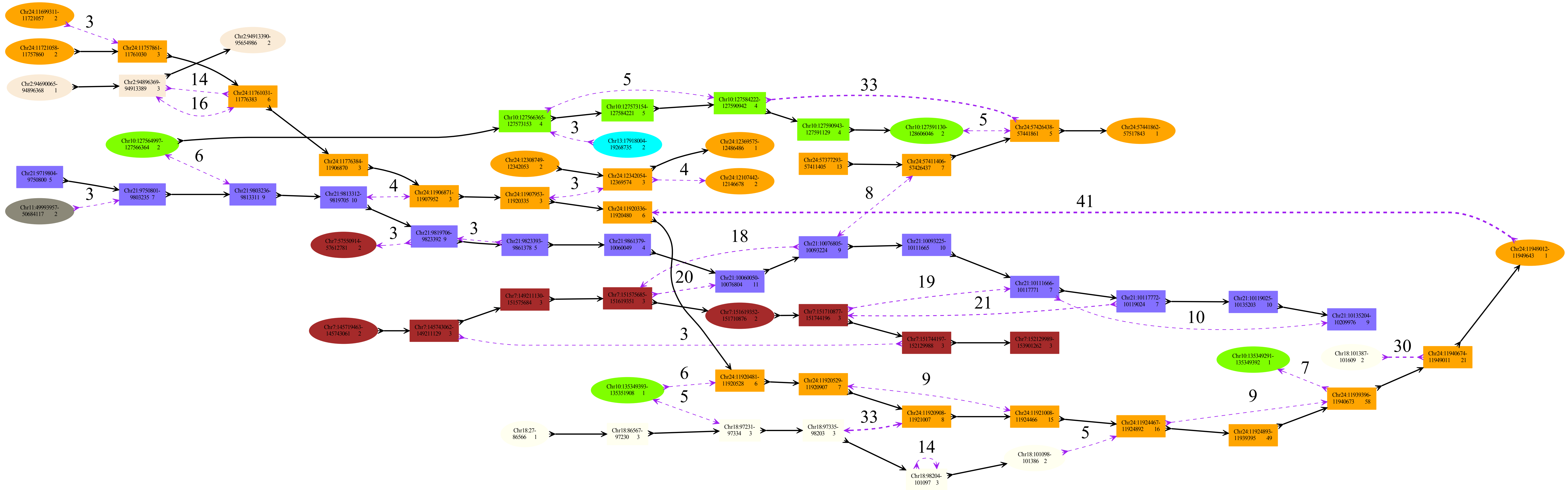

Supplement: Appendix S1 — Reconstruction of MCF-7 genome structure by fusion point guided concatenation method. (ZIP) [file pone.0046152.s017.zip › subgraph20.pdf]

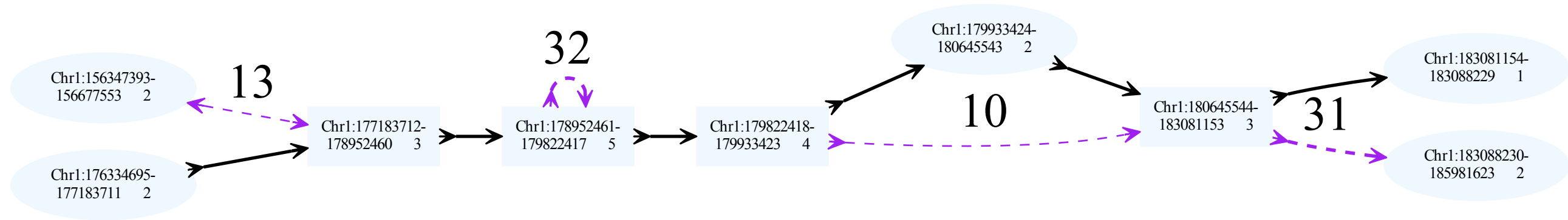

Supplement: Appendix S1 — Reconstruction of MCF-7 genome structure by fusion point guided concatenation method. (ZIP) [file pone.0046152.s017.zip › subgraph21.pdf]

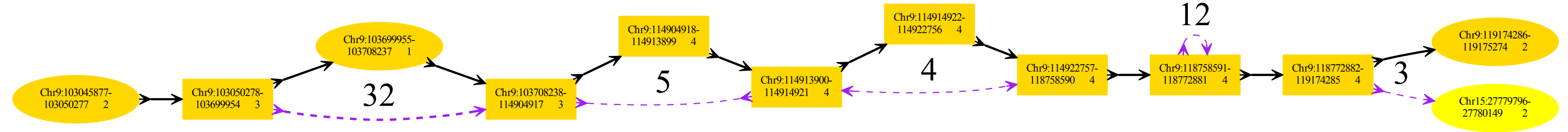

Supplement: Appendix S1 — Reconstruction of MCF-7 genome structure by fusion point guided concatenation method. (ZIP) [file pone.0046152.s017.zip › subgraph22.pdf]

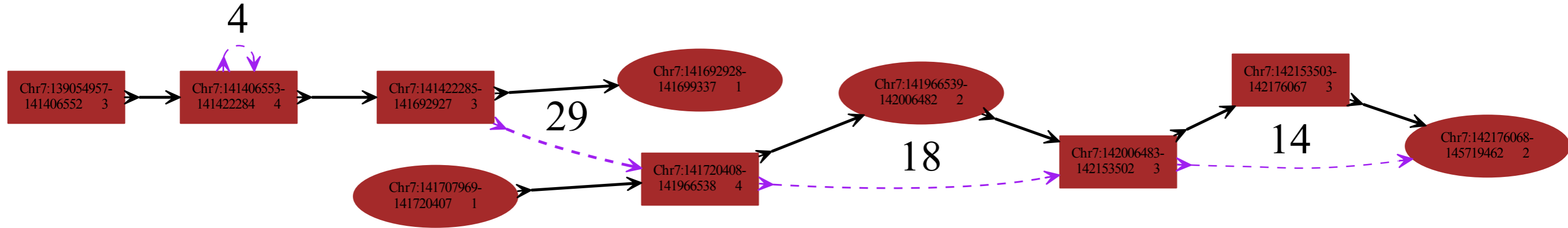

Supplement: Appendix S1 — Reconstruction of MCF-7 genome structure by fusion point guided concatenation method. (ZIP) [file pone.0046152.s017.zip › subgraph23.pdf]

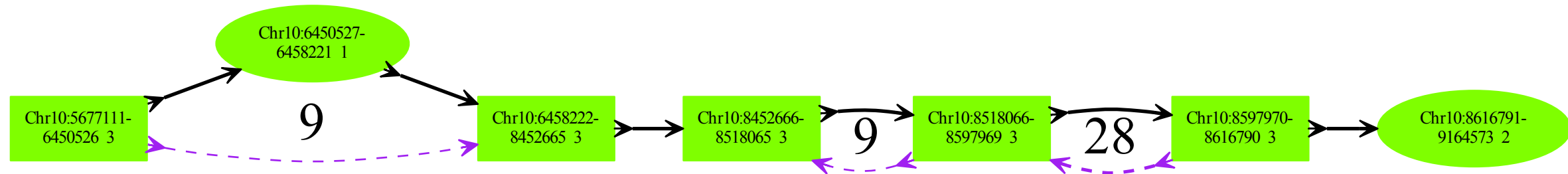

Supplement: Appendix S1 — Reconstruction of MCF-7 genome structure by fusion point guided concatenation method. (ZIP) [file pone.0046152.s017.zip › subgraph24.pdf]

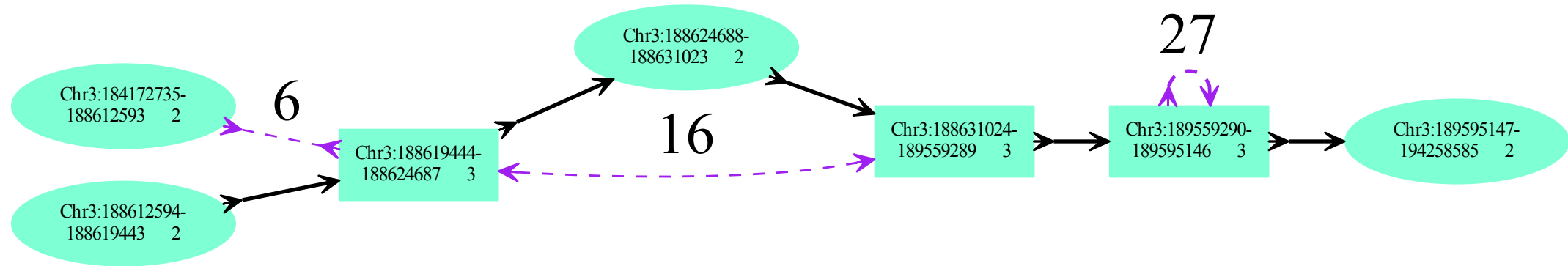

Supplement: Appendix S1 — Reconstruction of MCF-7 genome structure by fusion point guided concatenation method. (ZIP) [file pone.0046152.s017.zip › subgraph25.pdf]

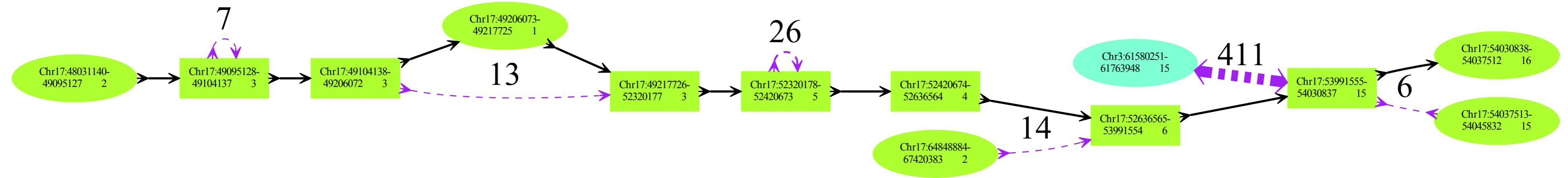

Supplement: Appendix S1 — Reconstruction of MCF-7 genome structure by fusion point guided concatenation method. (ZIP) [file pone.0046152.s017.zip › subgraph26.pdf]

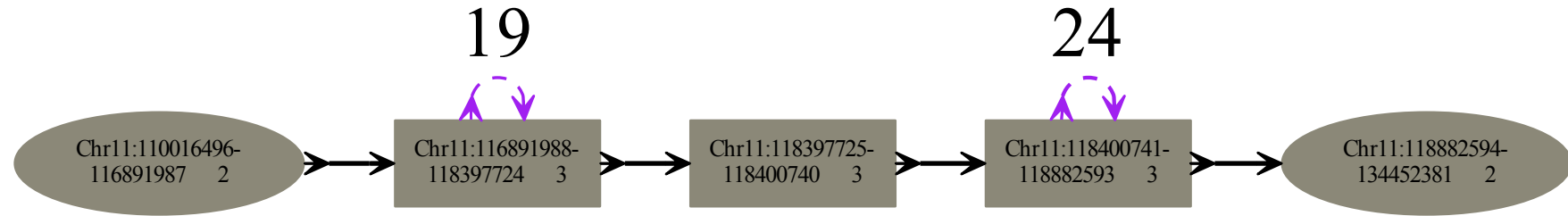

Supplement: Appendix S1 — Reconstruction of MCF-7 genome structure by fusion point guided concatenation method. (ZIP) [file pone.0046152.s017.zip › subgraph27.pdf]

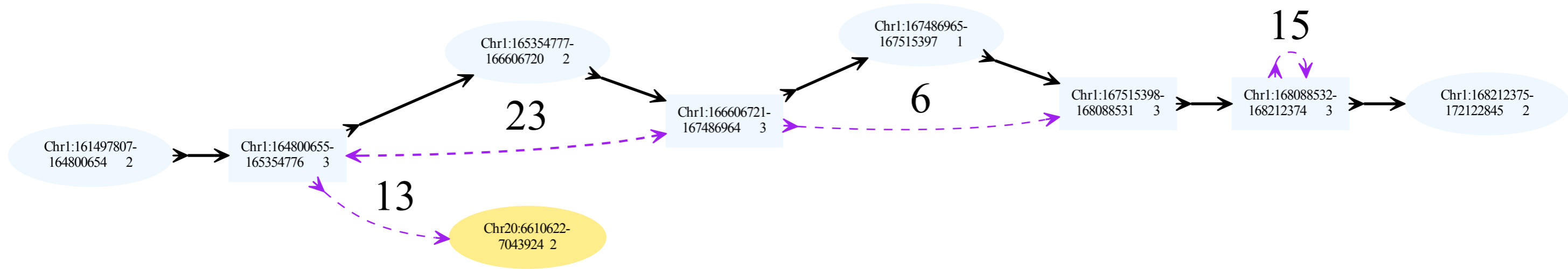

Supplement: Appendix S1 — Reconstruction of MCF-7 genome structure by fusion point guided concatenation method. (ZIP) [file pone.0046152.s017.zip › subgraph28.pdf]

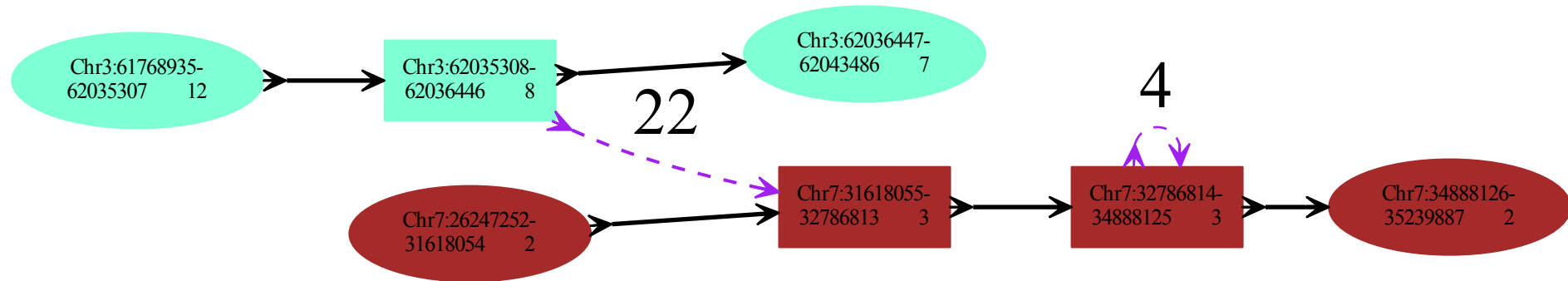

Supplement: Appendix S1 — Reconstruction of MCF-7 genome structure by fusion point guided concatenation method. (ZIP) [file pone.0046152.s017.zip › subgraph29.pdf]

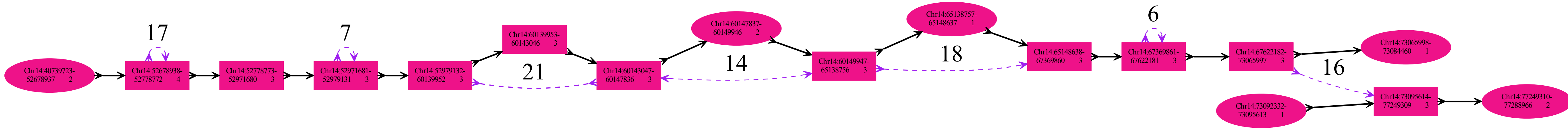

Supplement: Appendix S1 — Reconstruction of MCF-7 genome structure by fusion point guided concatenation method. (ZIP) [file pone.0046152.s017.zip › subgraph30.pdf]

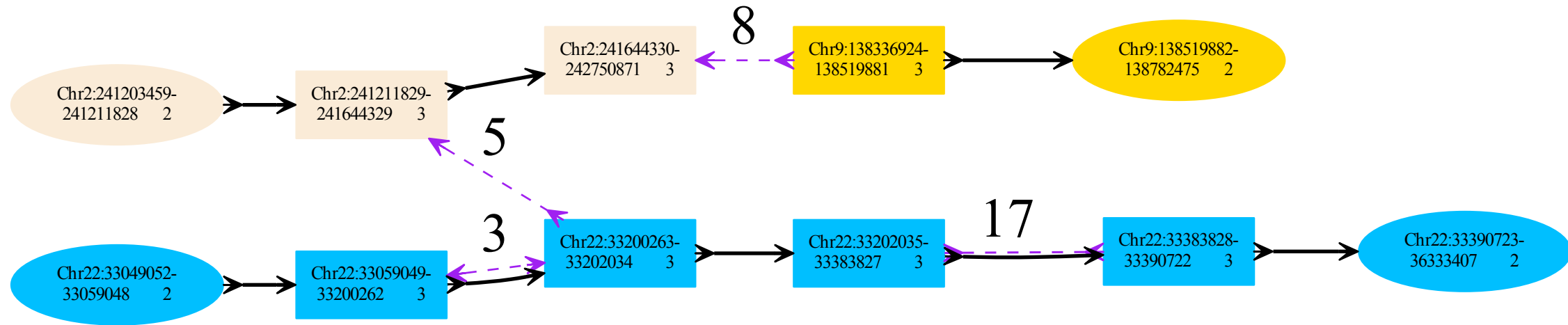

Supplement: Appendix S1 — Reconstruction of MCF-7 genome structure by fusion point guided concatenation method. (ZIP) [file pone.0046152.s017.zip › subgraph31.pdf]

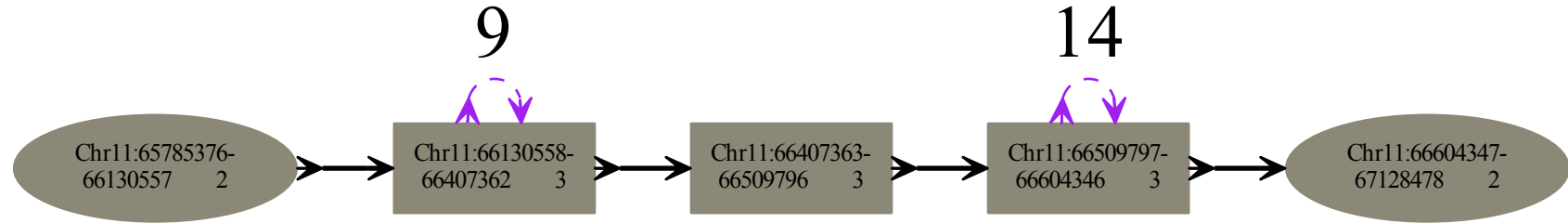

Supplement: Appendix S1 — Reconstruction of MCF-7 genome structure by fusion point guided concatenation method. (ZIP) [file pone.0046152.s017.zip › subgraph32.pdf]

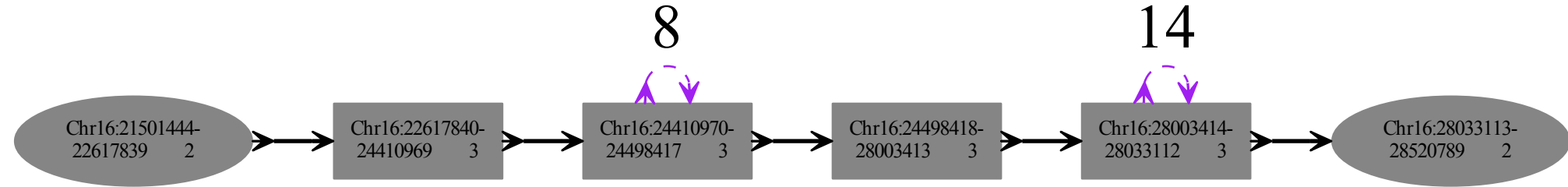

Supplement: Appendix S1 — Reconstruction of MCF-7 genome structure by fusion point guided concatenation method. (ZIP) [file pone.0046152.s017.zip › subgraph33.pdf]

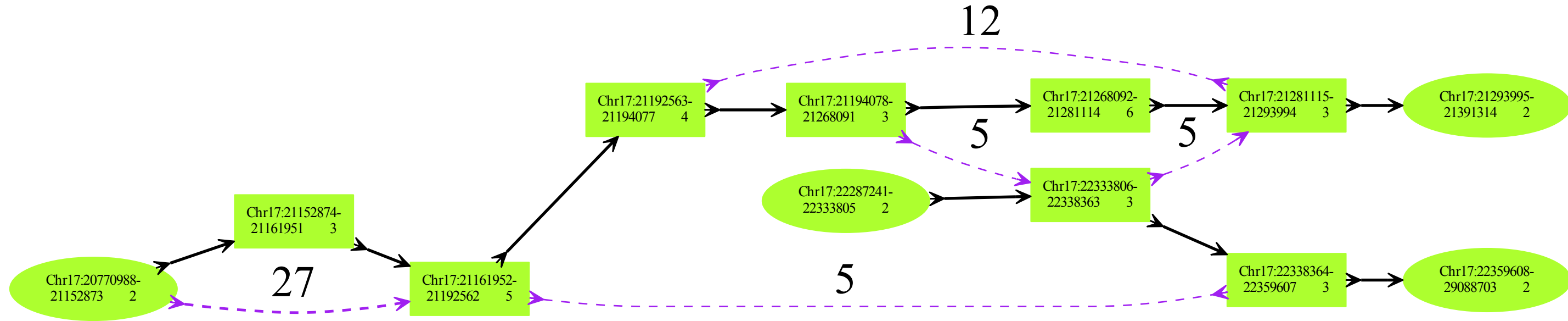

Supplement: Appendix S1 — Reconstruction of MCF-7 genome structure by fusion point guided concatenation method. (ZIP) [file pone.0046152.s017.zip › subgraph34.pdf]

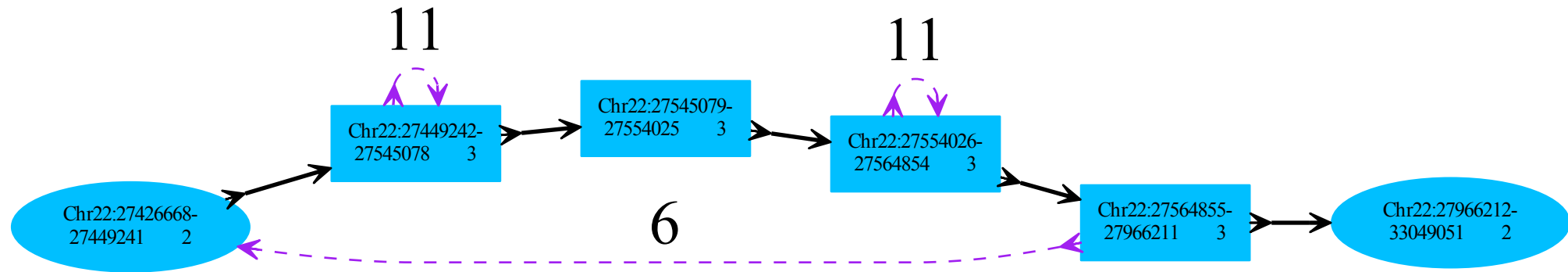

Supplement: Appendix S1 — Reconstruction of MCF-7 genome structure by fusion point guided concatenation method. (ZIP) [file pone.0046152.s017.zip › subgraph35.pdf]

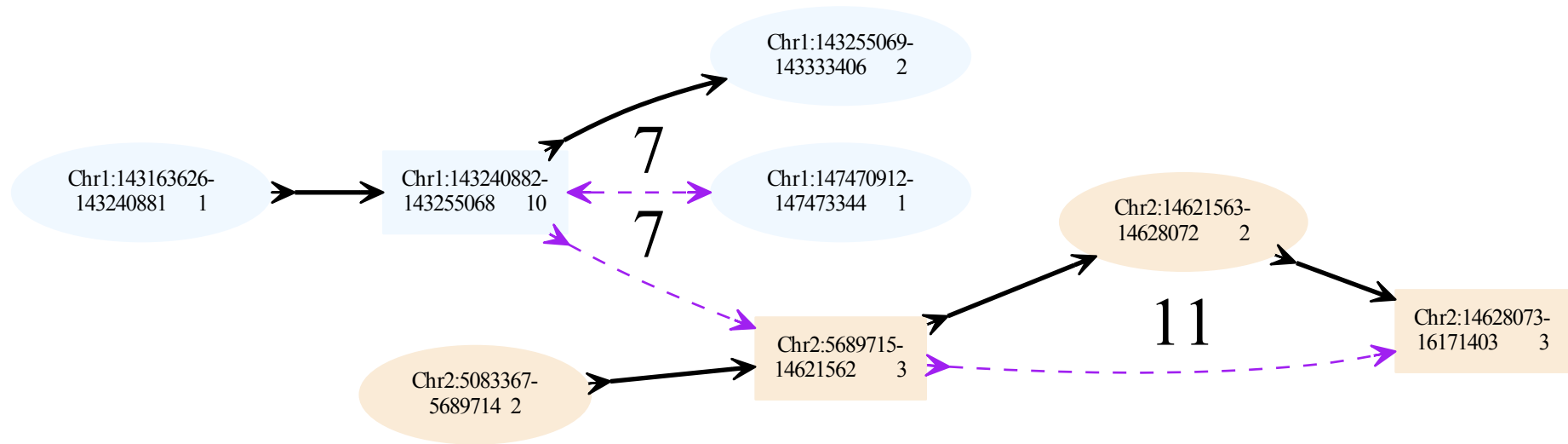

Supplement: Appendix S1 — Reconstruction of MCF-7 genome structure by fusion point guided concatenation method. (ZIP) [file pone.0046152.s017.zip › subgraph36.pdf]

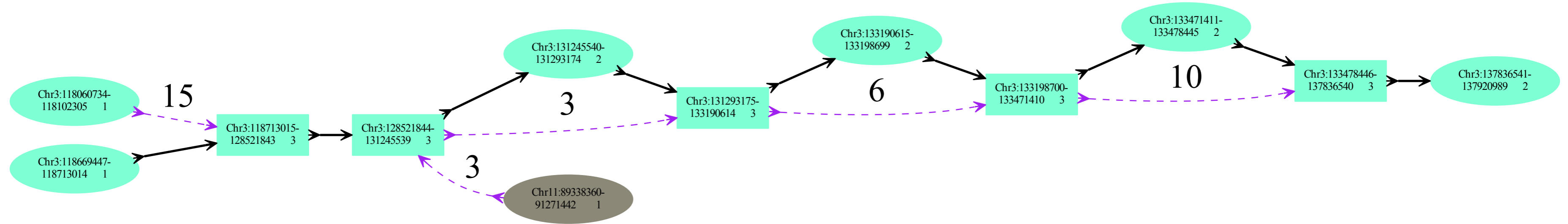

Supplement: Appendix S1 — Reconstruction of MCF-7 genome structure by fusion point guided concatenation method. (ZIP) [file pone.0046152.s017.zip › subgraph37.pdf]

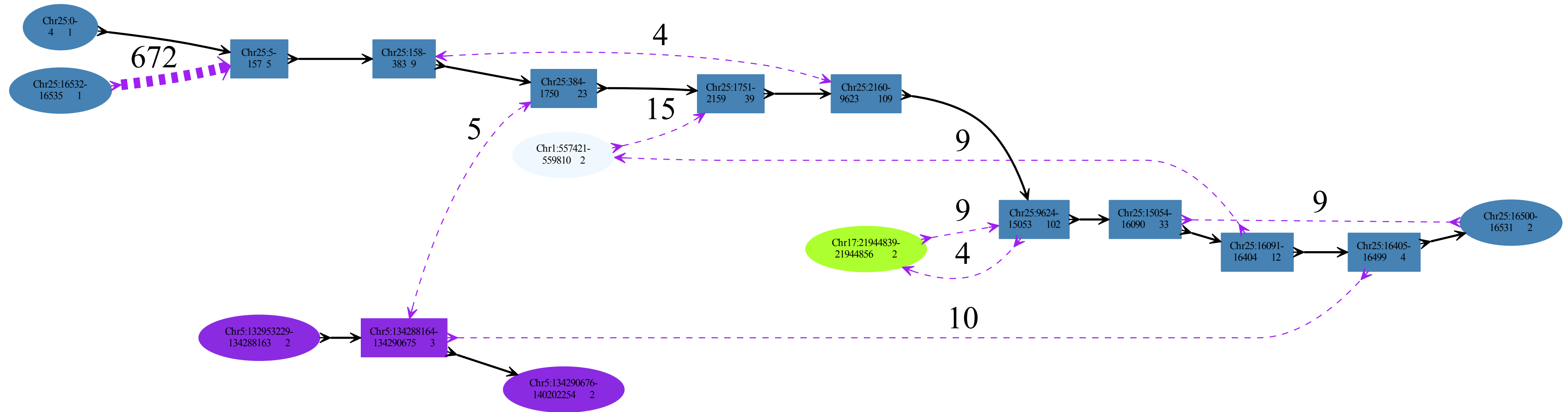

Supplement: Appendix S1 — Reconstruction of MCF-7 genome structure by fusion point guided concatenation method. (ZIP) [file pone.0046152.s017.zip › subgraph38.pdf]

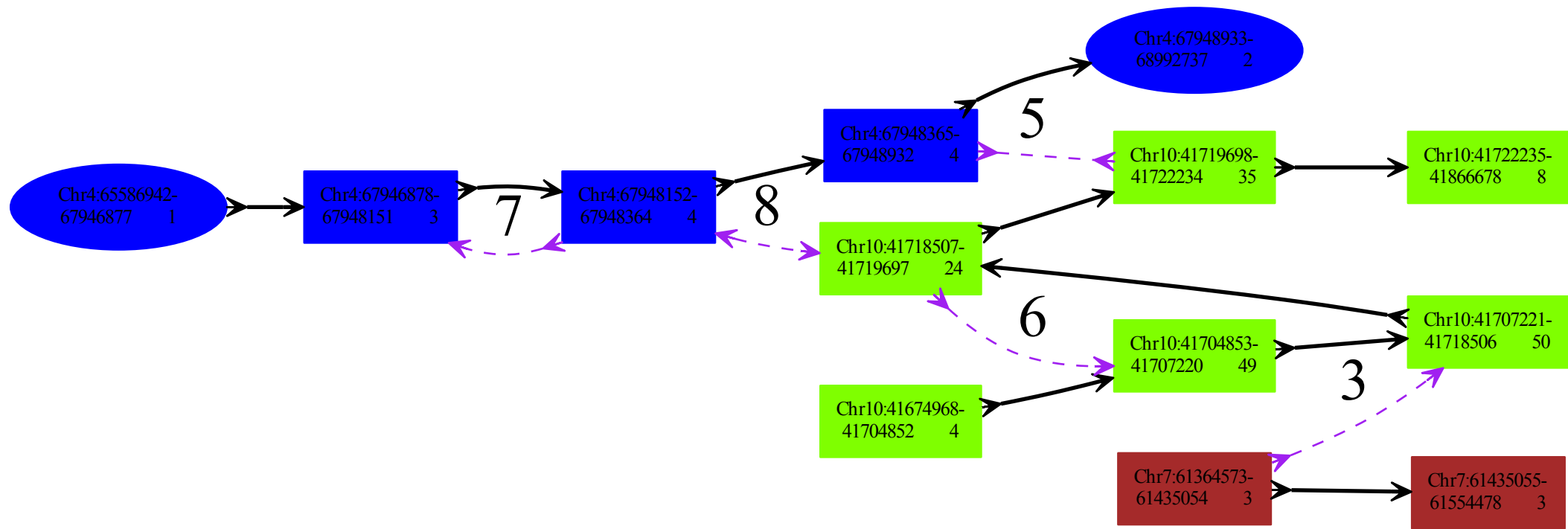

Supplement: Appendix S1 — Reconstruction of MCF-7 genome structure by fusion point guided concatenation method. (ZIP) [file pone.0046152.s017.zip › subgraph39.pdf]

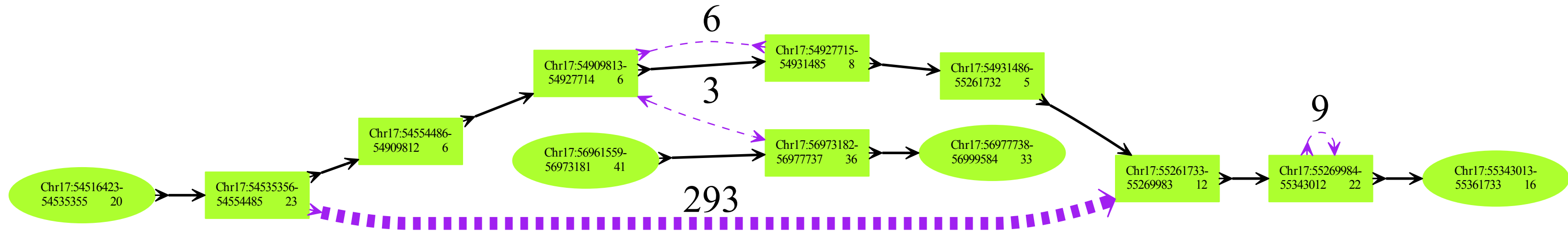

Supplement: Appendix S1 — Reconstruction of MCF-7 genome structure by fusion point guided concatenation method. (ZIP) [file pone.0046152.s017.zip › subgraph4.pdf]

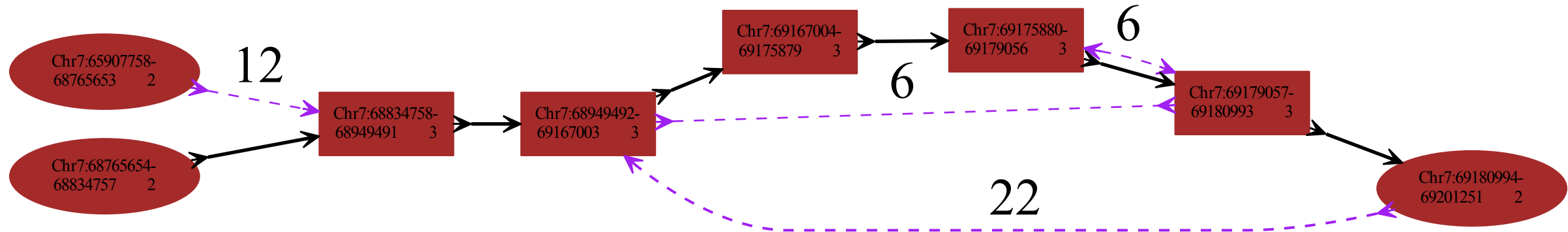

Supplement: Appendix S1 — Reconstruction of MCF-7 genome structure by fusion point guided concatenation method. (ZIP) [file pone.0046152.s017.zip › subgraph40.pdf]

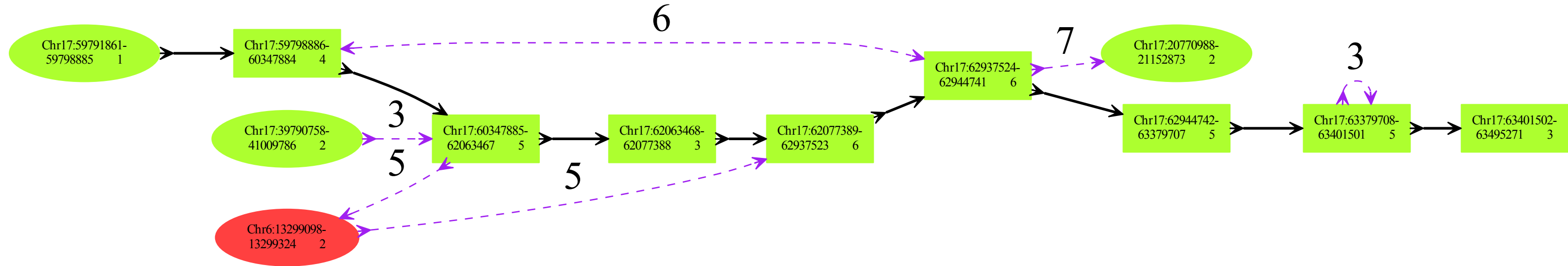

Supplement: Appendix S1 — Reconstruction of MCF-7 genome structure by fusion point guided concatenation method. (ZIP) [file pone.0046152.s017.zip › subgraph41.pdf]

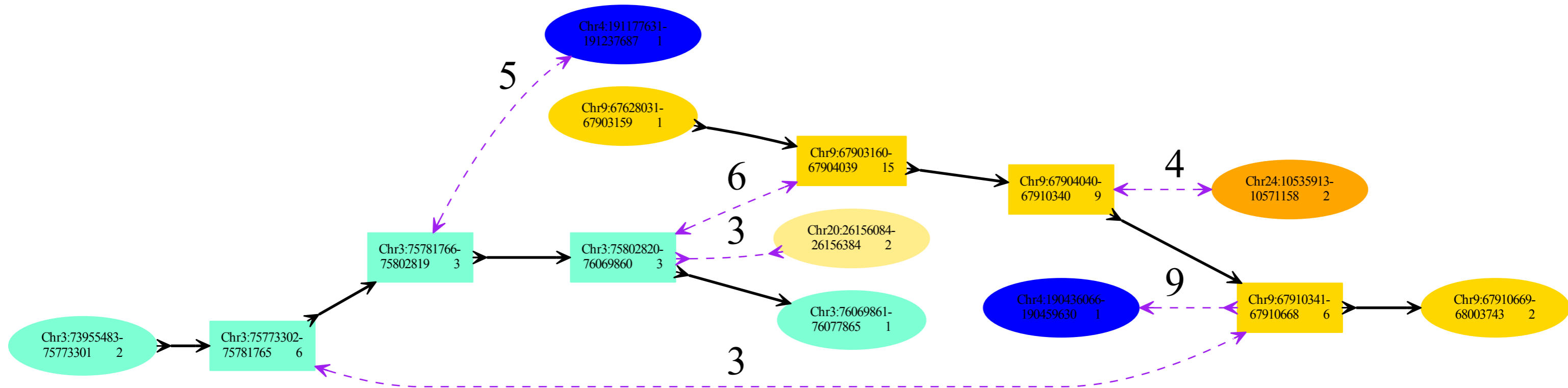

Supplement: Appendix S1 — Reconstruction of MCF-7 genome structure by fusion point guided concatenation method. (ZIP) [file pone.0046152.s017.zip › subgraph42.pdf]

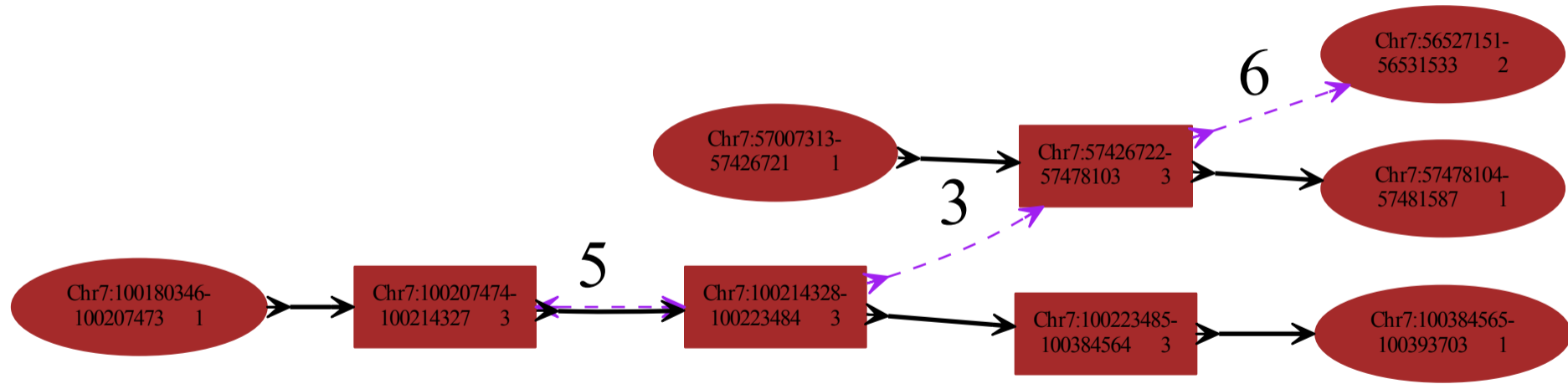

Supplement: Appendix S1 — Reconstruction of MCF-7 genome structure by fusion point guided concatenation method. (ZIP) [file pone.0046152.s017.zip › subgraph43.pdf]

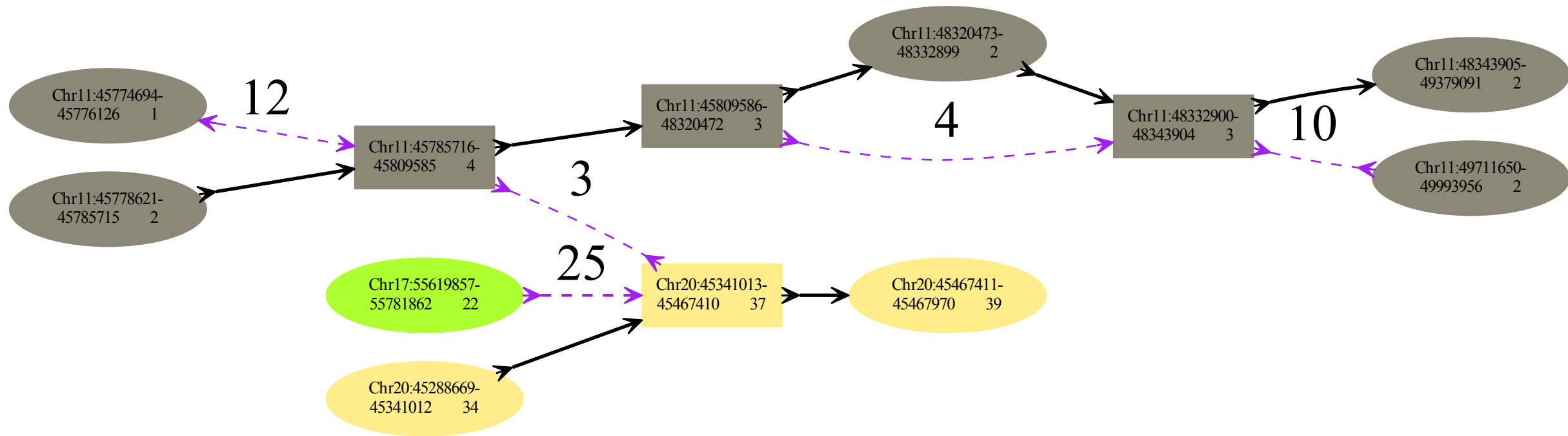

Supplement: Appendix S1 — Reconstruction of MCF-7 genome structure by fusion point guided concatenation method. (ZIP) [file pone.0046152.s017.zip › subgraph44.pdf]

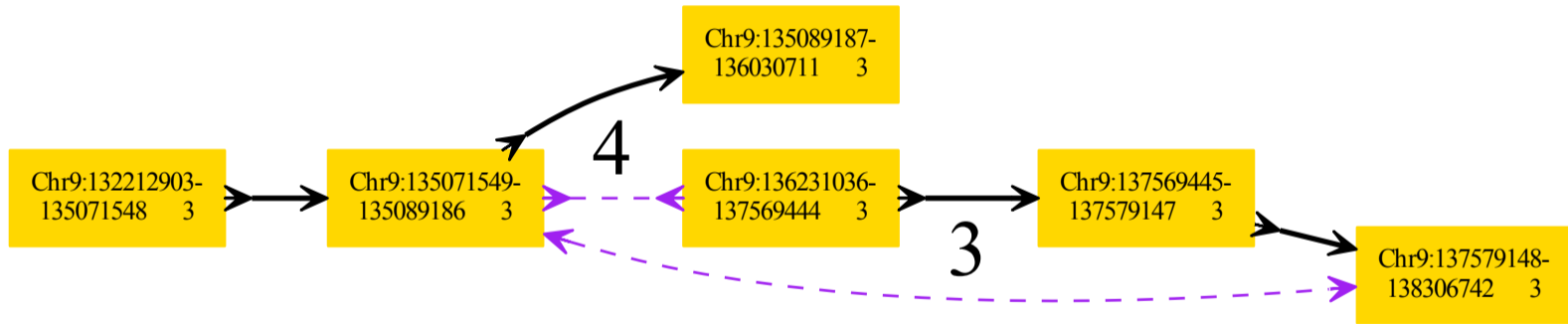

Supplement: Appendix S1 — Reconstruction of MCF-7 genome structure by fusion point guided concatenation method. (ZIP) [file pone.0046152.s017.zip › subgraph45.pdf]

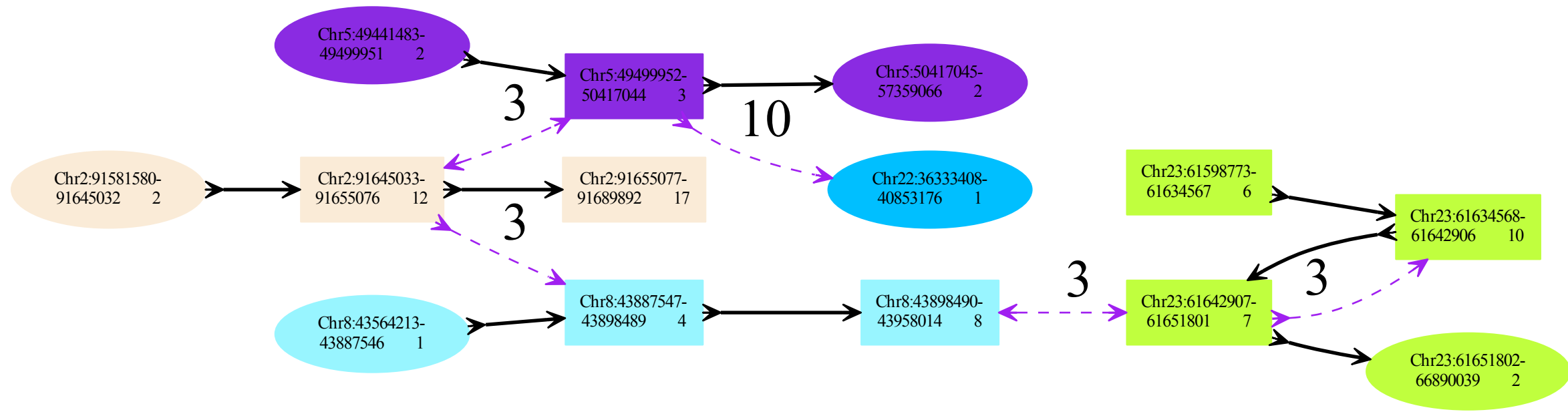

Supplement: Appendix S1 — Reconstruction of MCF-7 genome structure by fusion point guided concatenation method. (ZIP) [file pone.0046152.s017.zip › subgraph46.pdf]

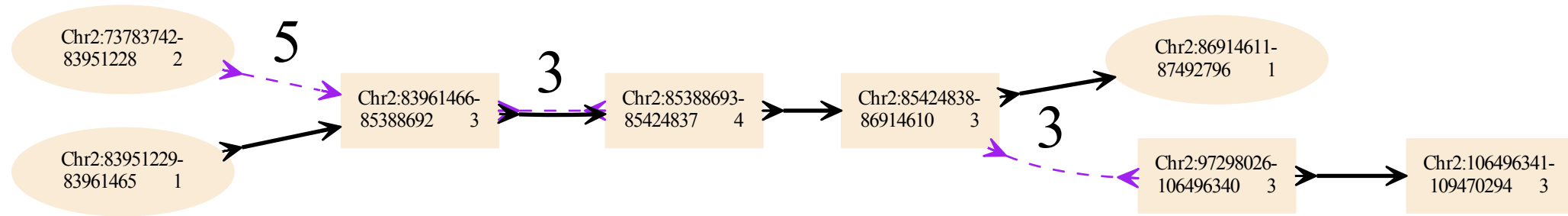

Supplement: Appendix S1 — Reconstruction of MCF-7 genome structure by fusion point guided concatenation method. (ZIP) [file pone.0046152.s017.zip › subgraph47.pdf]

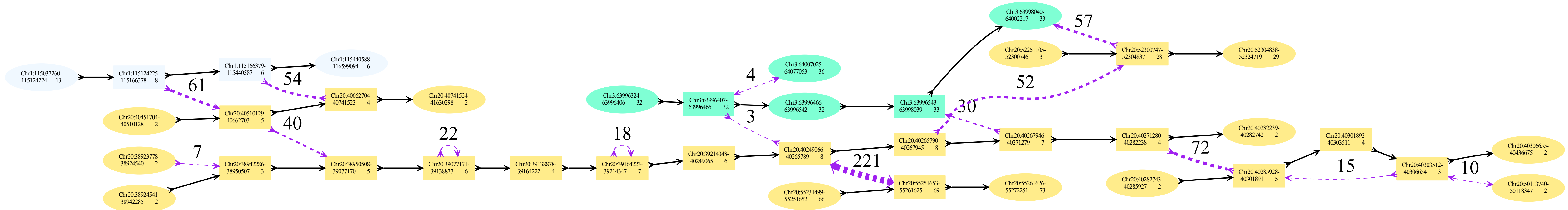

Supplement: Appendix S1 — Reconstruction of MCF-7 genome structure by fusion point guided concatenation method. (ZIP) [file pone.0046152.s017.zip › subgraph5.pdf]

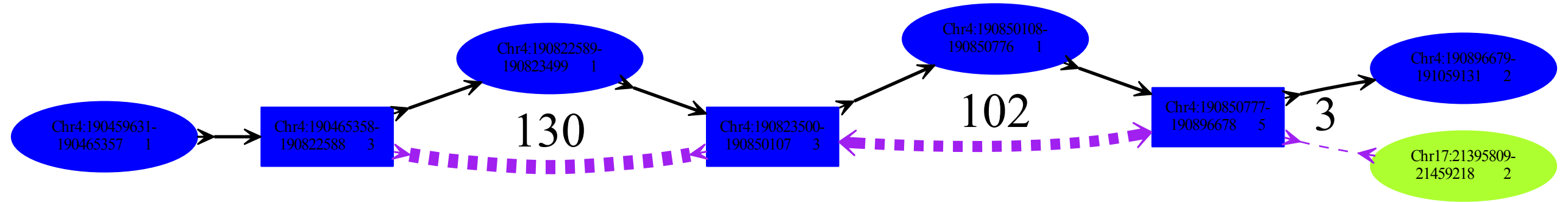

Supplement: Appendix S1 — Reconstruction of MCF-7 genome structure by fusion point guided concatenation method. (ZIP) [file pone.0046152.s017.zip › subgraph6.pdf]

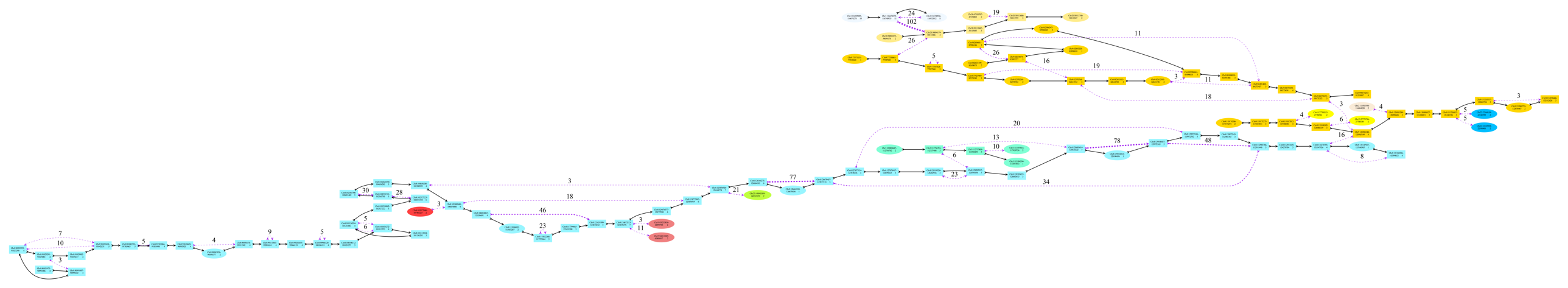

Supplement: Appendix S1 — Reconstruction of MCF-7 genome structure by fusion point guided concatenation method. (ZIP) [file pone.0046152.s017.zip › subgraph7.pdf]

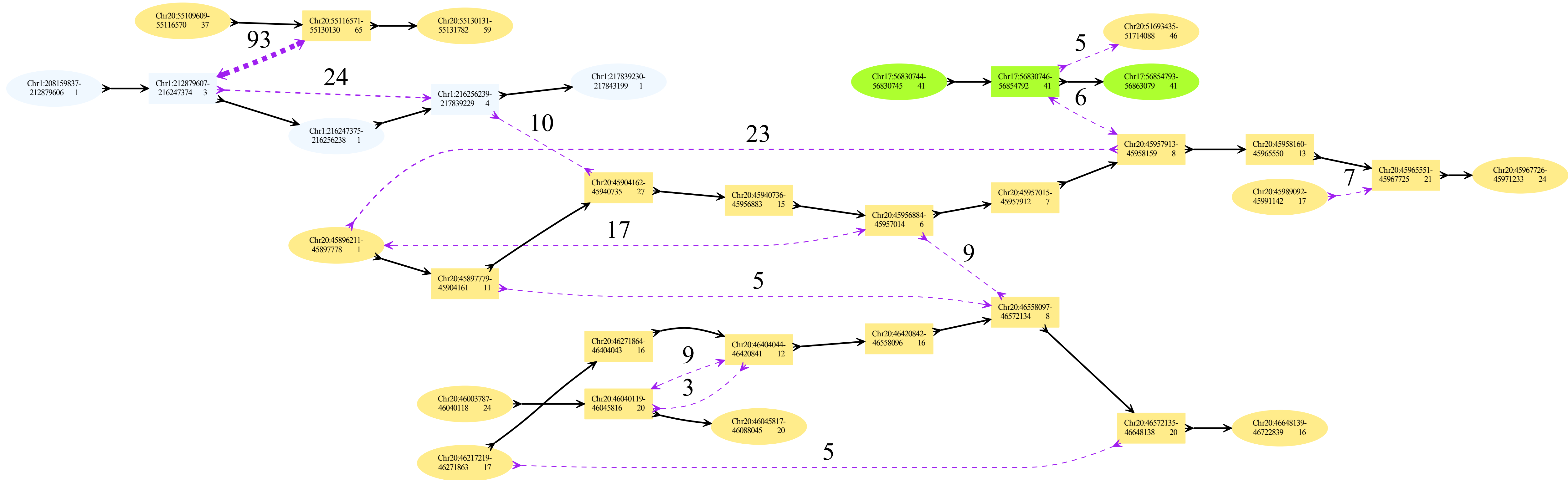

Supplement: Appendix S1 — Reconstruction of MCF-7 genome structure by fusion point guided concatenation method. (ZIP) [file pone.0046152.s017.zip › subgraph8.pdf]

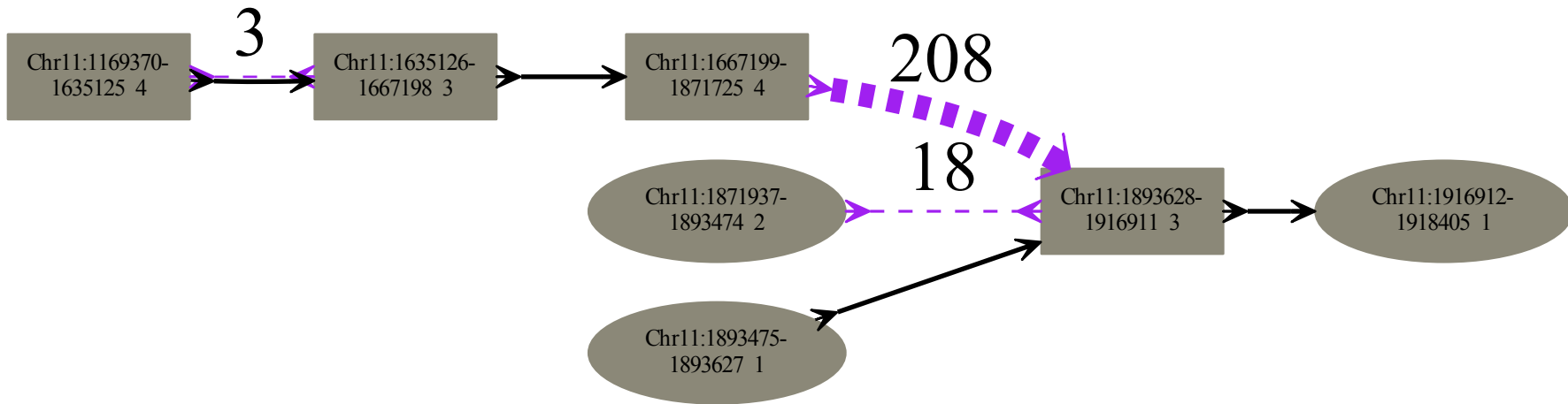

Supplement: Appendix S2 — Reconstruction of HCT116 genome structure by fusion point guided concatenation method. (ZIP) [file pone.0046152.s018.zip › subgraph1.pdf]

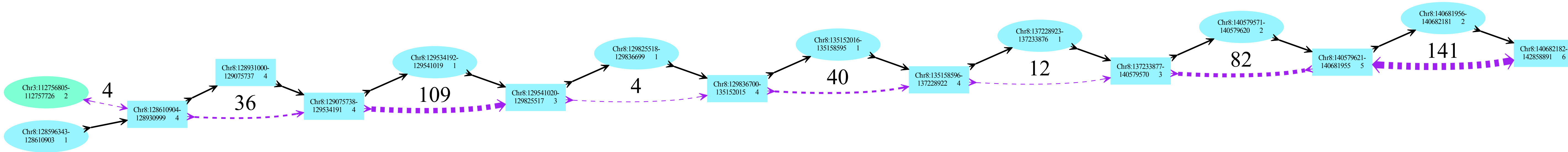

Supplement: Appendix S2 — Reconstruction of HCT116 genome structure by fusion point guided concatenation method. (ZIP) [file pone.0046152.s018.zip › subgraph2.pdf]

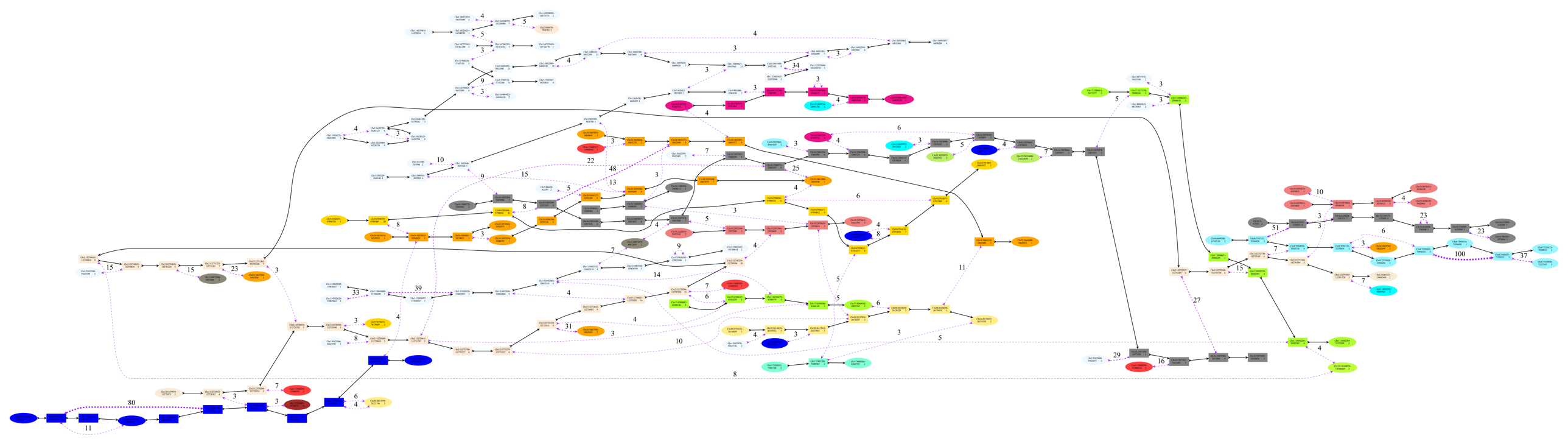

Supplement: Appendix S2 — Reconstruction of HCT116 genome structure by fusion point guided concatenation method. (ZIP) [file pone.0046152.s018.zip › subgraph3.pdf]

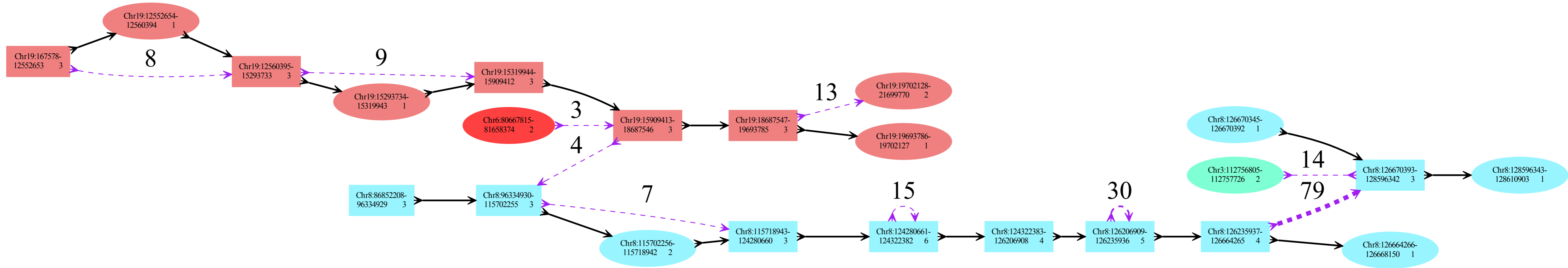

Supplement: Appendix S2 — Reconstruction of HCT116 genome structure by fusion point guided concatenation method. (ZIP) [file pone.0046152.s018.zip › subgraph4.pdf]

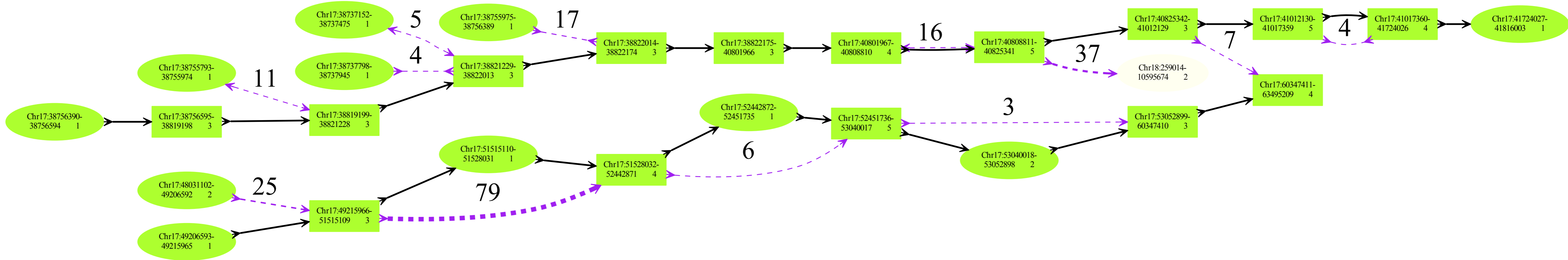

Supplement: Appendix S2 — Reconstruction of HCT116 genome structure by fusion point guided concatenation method. (ZIP) [file pone.0046152.s018.zip › subgraph5.pdf]

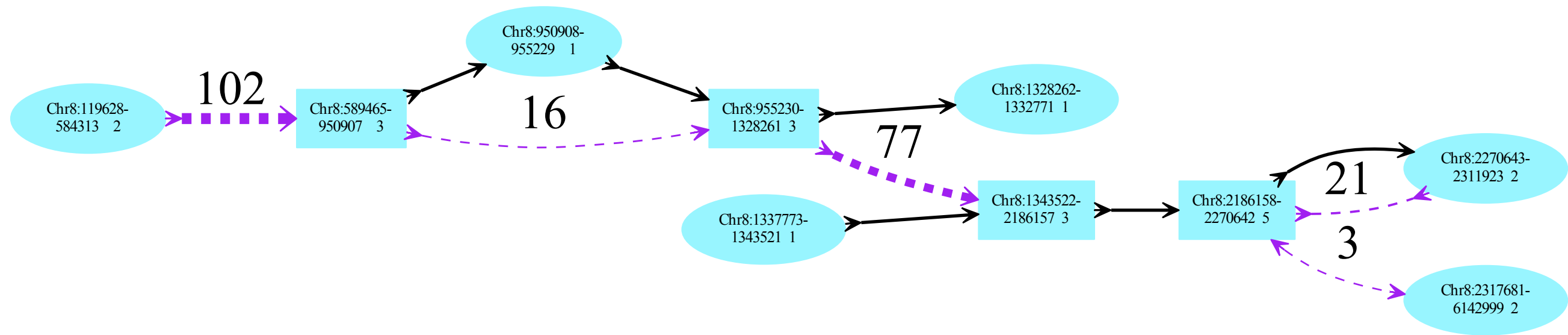

Supplement: Appendix S2 — Reconstruction of HCT116 genome structure by fusion point guided concatenation method. (ZIP) [file pone.0046152.s018.zip › subgraph6.pdf]

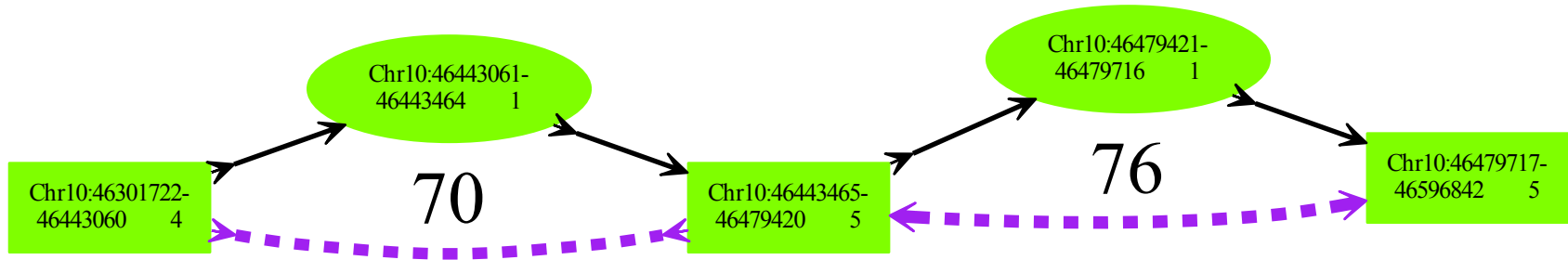

Supplement: Appendix S2 — Reconstruction of HCT116 genome structure by fusion point guided concatenation method. (ZIP) [file pone.0046152.s018.zip › subgraph7.pdf]

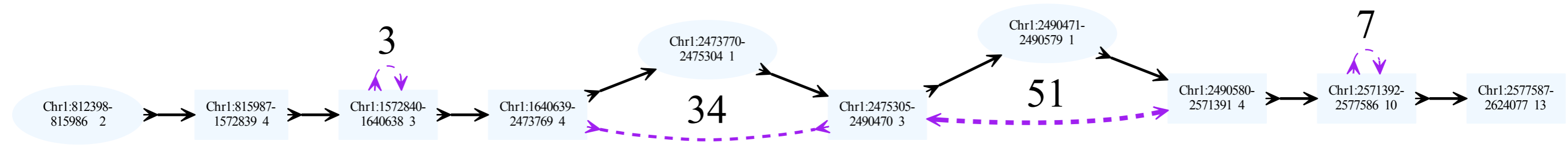

Supplement: Appendix S2 — Reconstruction of HCT116 genome structure by fusion point guided concatenation method. (ZIP) [file pone.0046152.s018.zip › subgraph9.pdf]

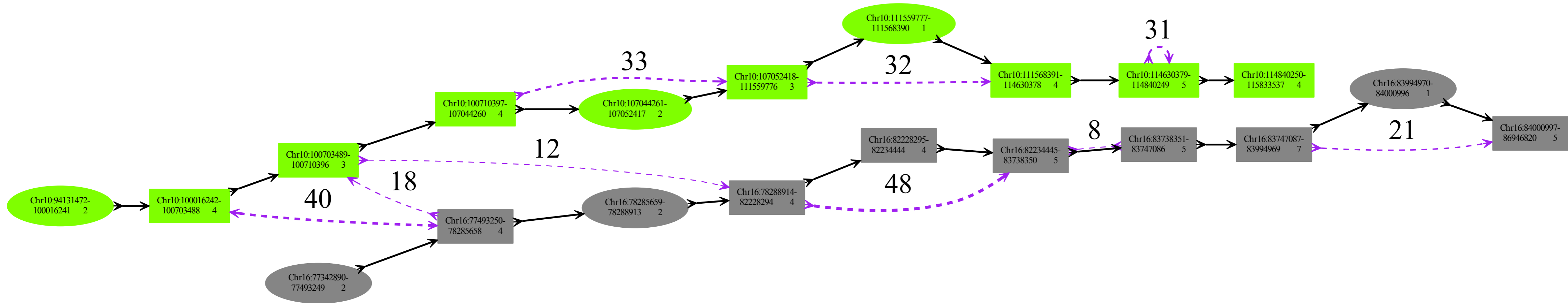

Supplement: Appendix S2 — Reconstruction of HCT116 genome structure by fusion point guided concatenation method. (ZIP) [file pone.0046152.s018.zip › subgraph10.pdf]

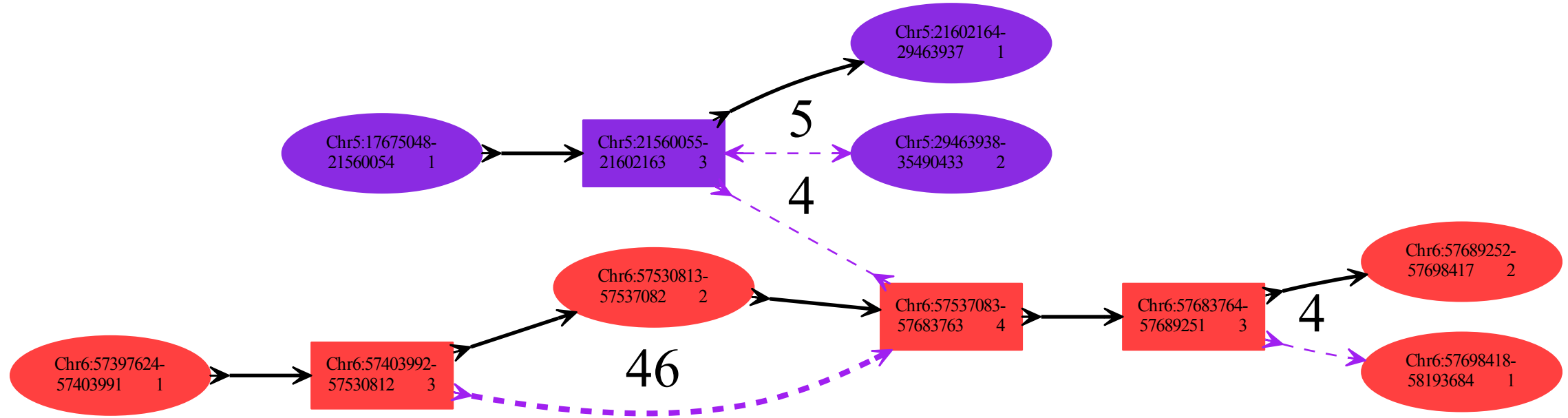

Supplement: Appendix S2 — Reconstruction of HCT116 genome structure by fusion point guided concatenation method. (ZIP) [file pone.0046152.s018.zip › subgraph11.pdf]

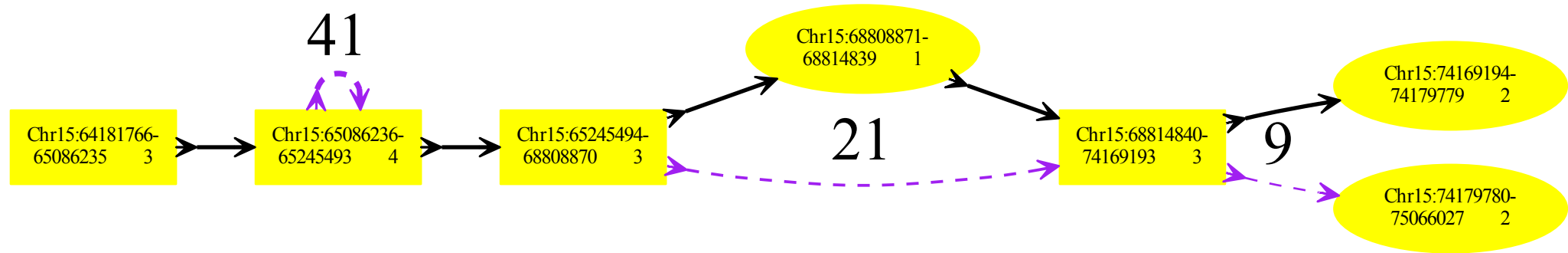

Supplement: Appendix S2 — Reconstruction of HCT116 genome structure by fusion point guided concatenation method. (ZIP) [file pone.0046152.s018.zip › subgraph12.pdf]

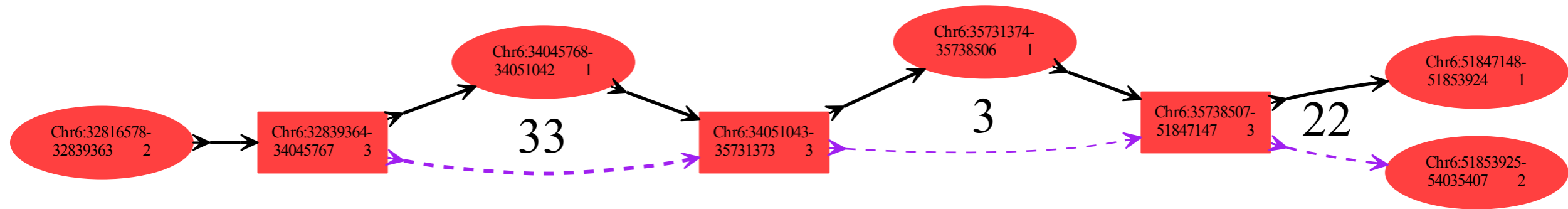

Supplement: Appendix S2 — Reconstruction of HCT116 genome structure by fusion point guided concatenation method. (ZIP) [file pone.0046152.s018.zip › subgraph13.pdf]

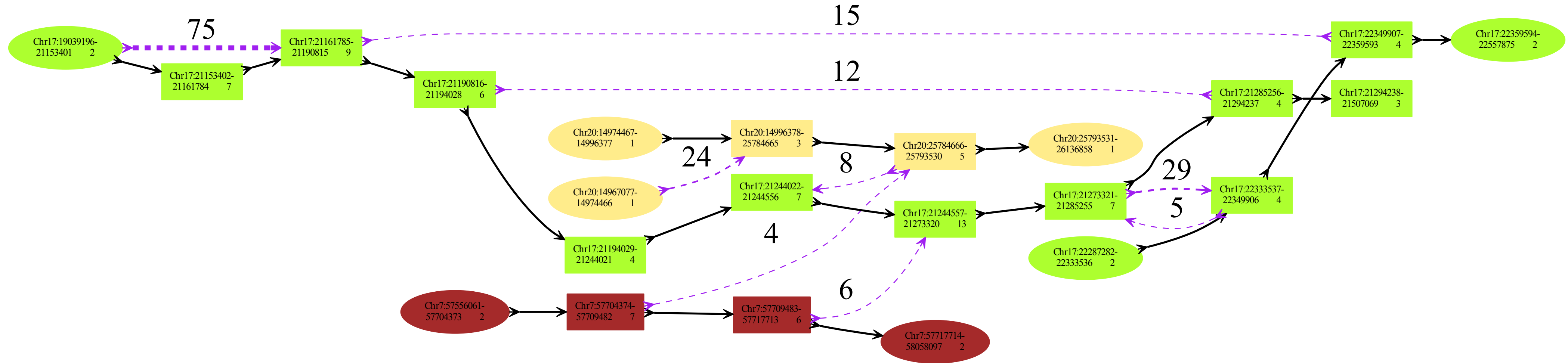

Supplement: Appendix S2 — Reconstruction of HCT116 genome structure by fusion point guided concatenation method. (ZIP) [file pone.0046152.s018.zip › subgraph14.pdf]

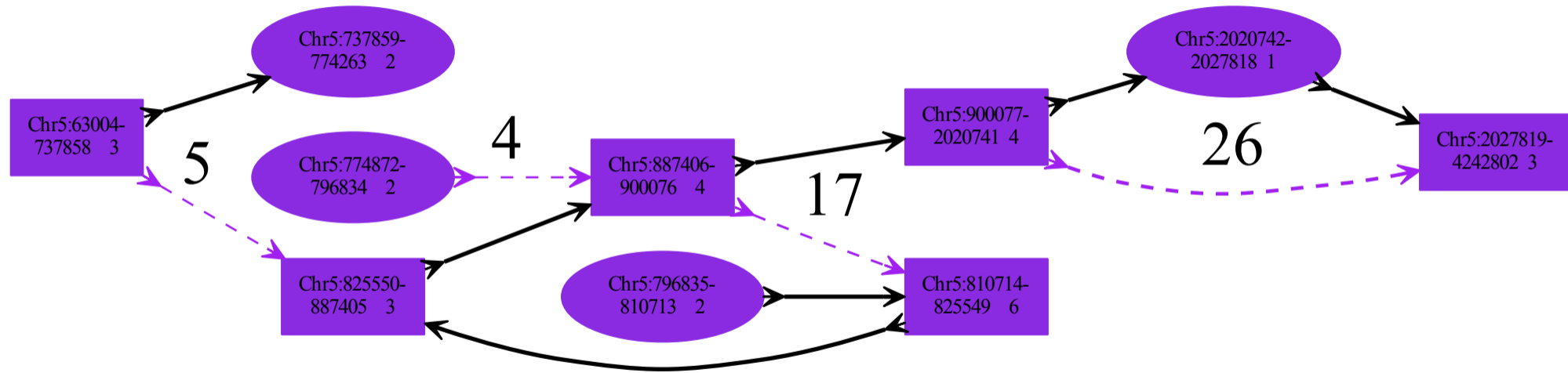

Supplement: Appendix S2 — Reconstruction of HCT116 genome structure by fusion point guided concatenation method. (ZIP) [file pone.0046152.s018.zip › subgraph15.pdf]

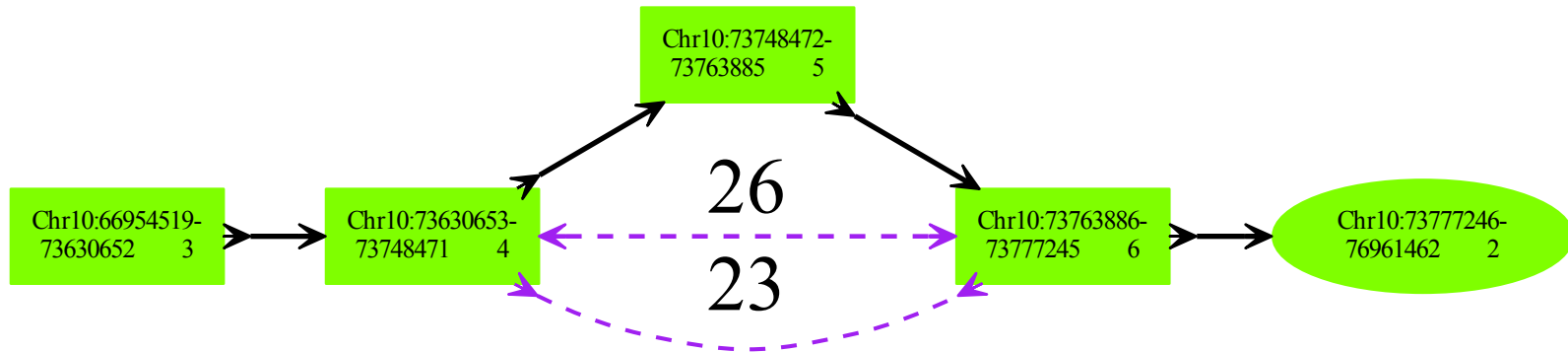

Supplement: Appendix S2 — Reconstruction of HCT116 genome structure by fusion point guided concatenation method. (ZIP) [file pone.0046152.s018.zip › subgraph16.pdf]

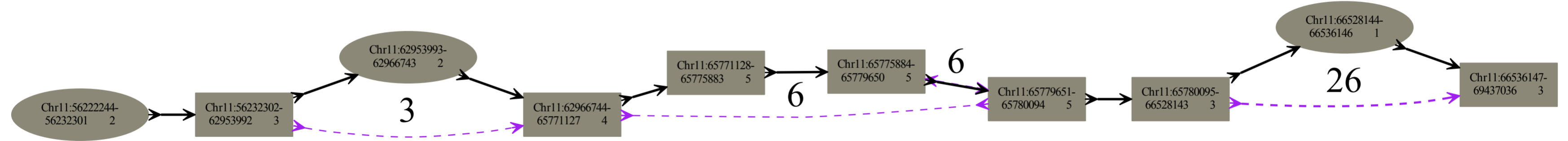

Supplement: Appendix S2 — Reconstruction of HCT116 genome structure by fusion point guided concatenation method. (ZIP) [file pone.0046152.s018.zip › subgraph17.pdf]

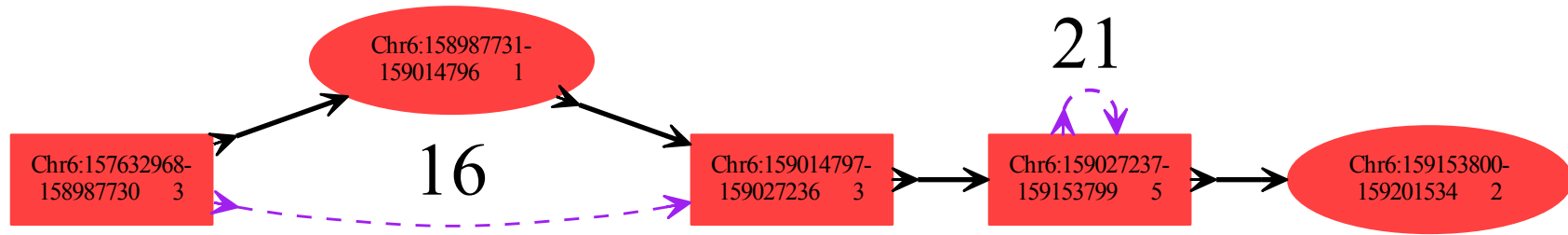

Supplement: Appendix S2 — Reconstruction of HCT116 genome structure by fusion point guided concatenation method. (ZIP) [file pone.0046152.s018.zip › subgraph18.pdf]

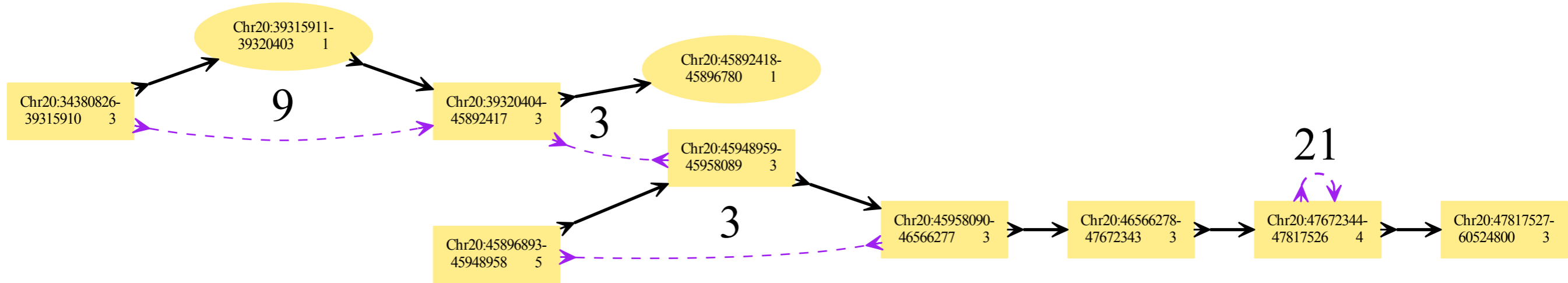

Supplement: Appendix S2 — Reconstruction of HCT116 genome structure by fusion point guided concatenation method. (ZIP) [file pone.0046152.s018.zip › subgraph19.pdf]

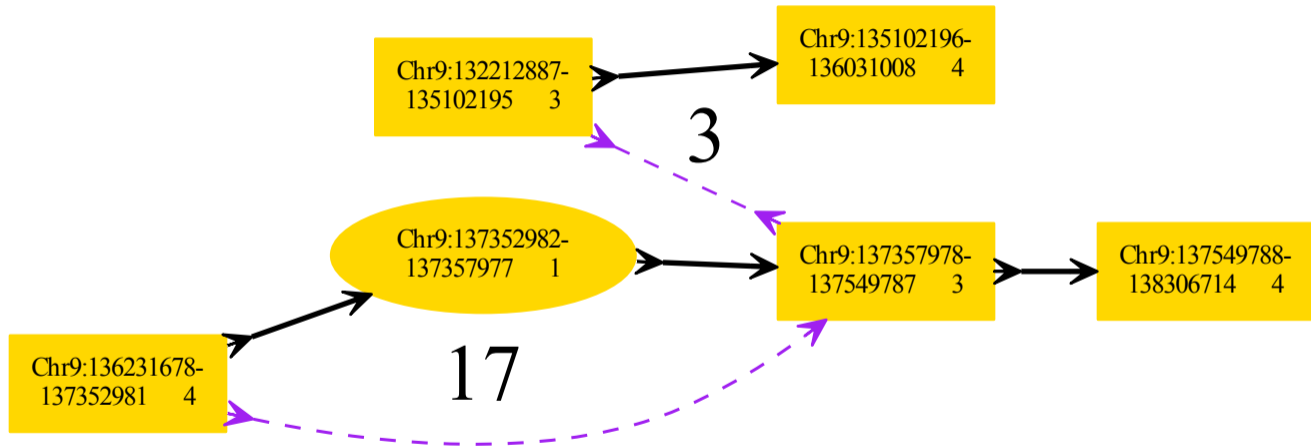

Supplement: Appendix S2 — Reconstruction of HCT116 genome structure by fusion point guided concatenation method. (ZIP) [file pone.0046152.s018.zip › subgraph20.pdf]

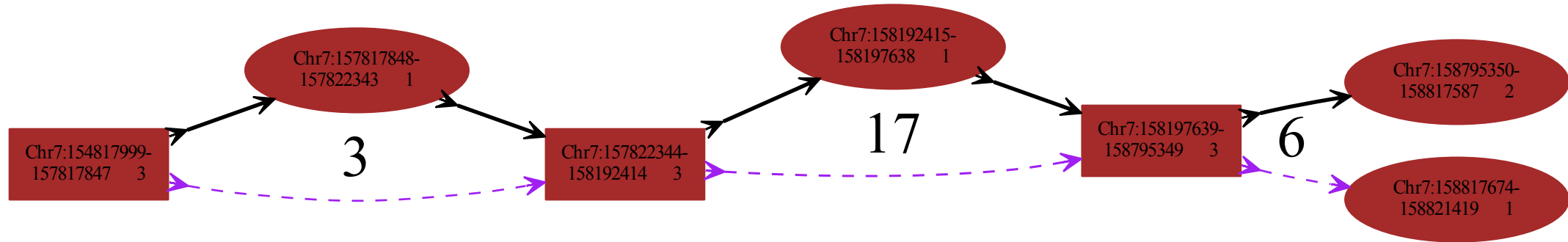

Supplement: Appendix S2 — Reconstruction of HCT116 genome structure by fusion point guided concatenation method. (ZIP) [file pone.0046152.s018.zip › subgraph21.pdf]

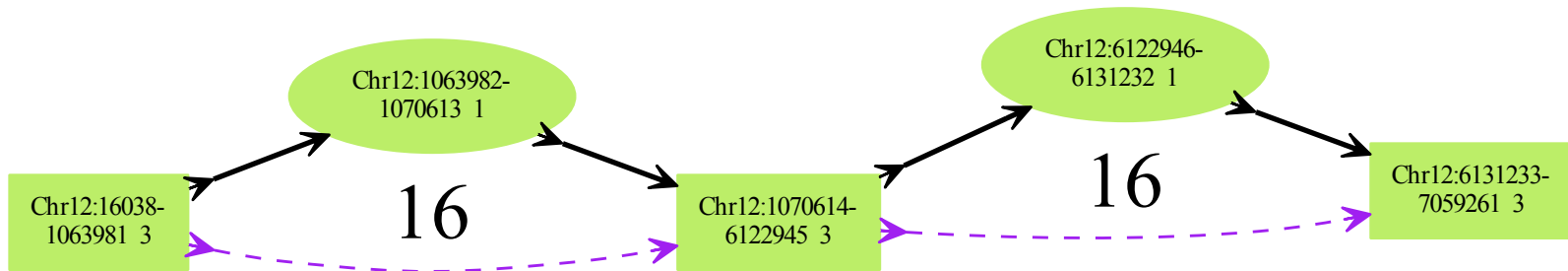

Supplement: Appendix S2 — Reconstruction of HCT116 genome structure by fusion point guided concatenation method. (ZIP) [file pone.0046152.s018.zip › subgraph22.pdf]

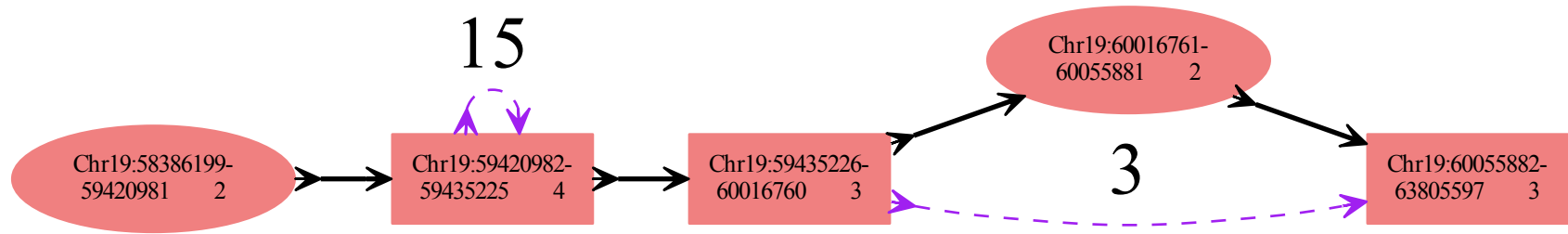

Supplement: Appendix S2 — Reconstruction of HCT116 genome structure by fusion point guided concatenation method. (ZIP) [file pone.0046152.s018.zip › subgraph23.pdf]

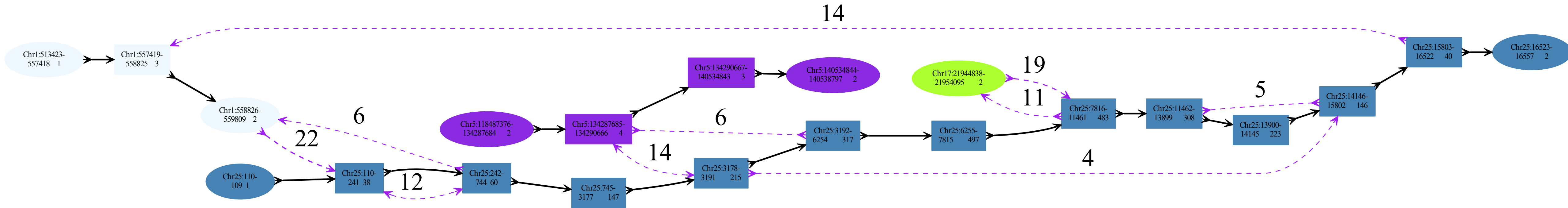

Supplement: Appendix S2 — Reconstruction of HCT116 genome structure by fusion point guided concatenation method. (ZIP) [file pone.0046152.s018.zip › subgraph24.pdf]

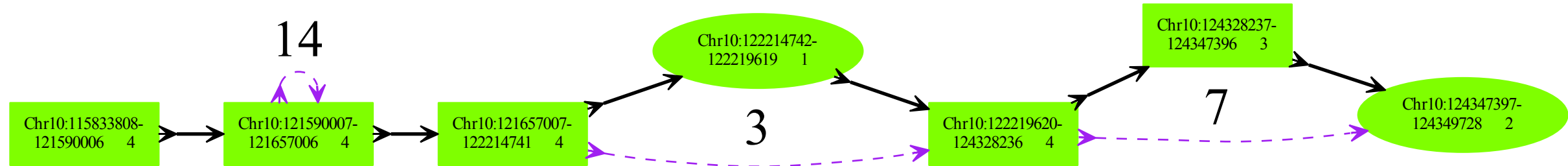

Supplement: Appendix S2 — Reconstruction of HCT116 genome structure by fusion point guided concatenation method. (ZIP) [file pone.0046152.s018.zip › subgraph25.pdf]

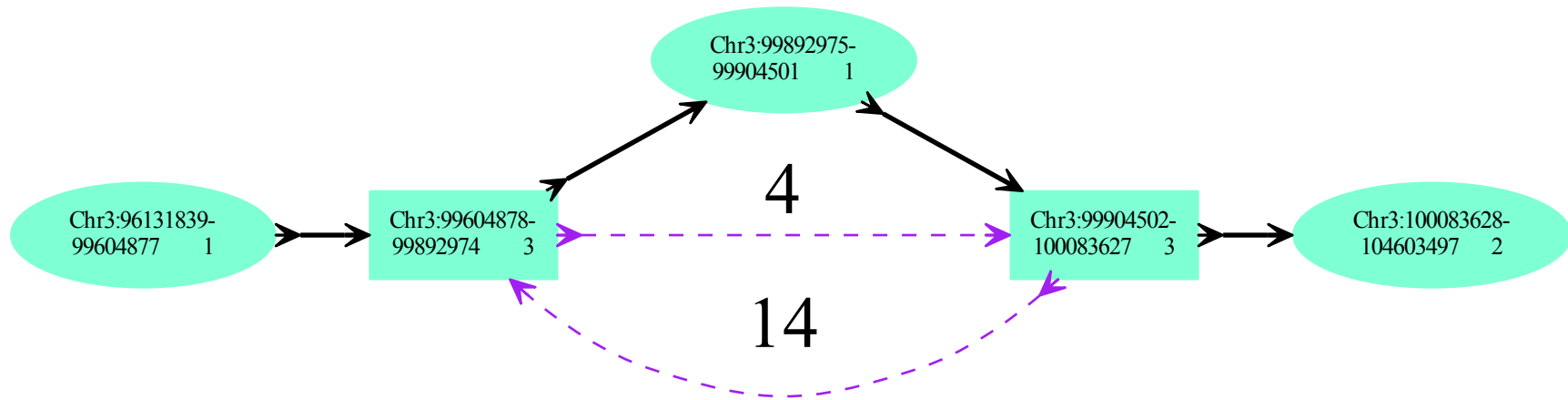

Supplement: Appendix S2 — Reconstruction of HCT116 genome structure by fusion point guided concatenation method. (ZIP) [file pone.0046152.s018.zip › subgraph26.pdf]

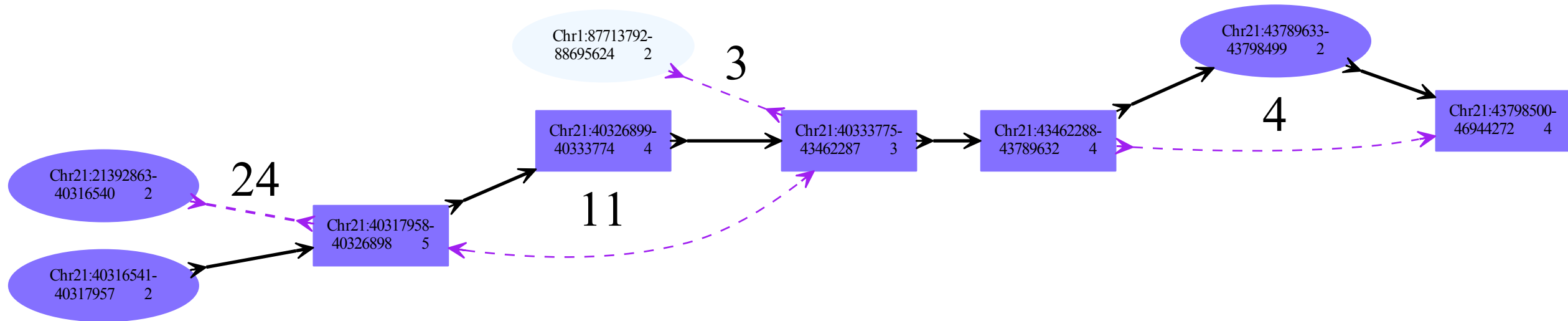

Supplement: Appendix S2 — Reconstruction of HCT116 genome structure by fusion point guided concatenation method. (ZIP) [file pone.0046152.s018.zip › subgraph27.pdf]

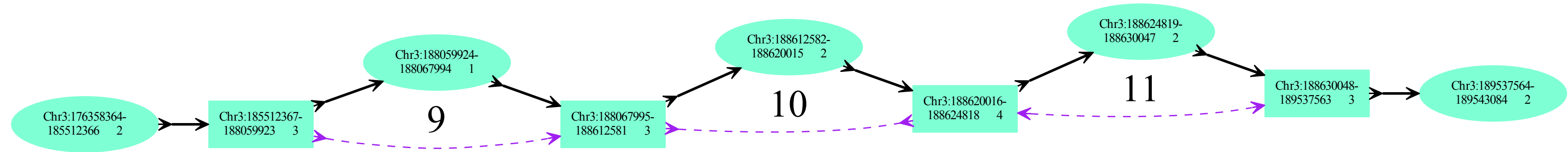

Supplement: Appendix S2 — Reconstruction of HCT116 genome structure by fusion point guided concatenation method. (ZIP) [file pone.0046152.s018.zip › subgraph28.pdf]

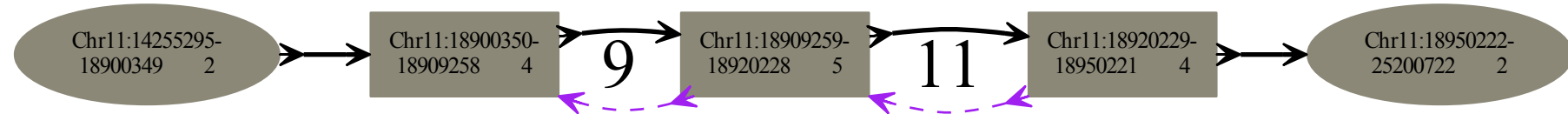

Supplement: Appendix S2 — Reconstruction of HCT116 genome structure by fusion point guided concatenation method. (ZIP) [file pone.0046152.s018.zip › subgraph29.pdf]

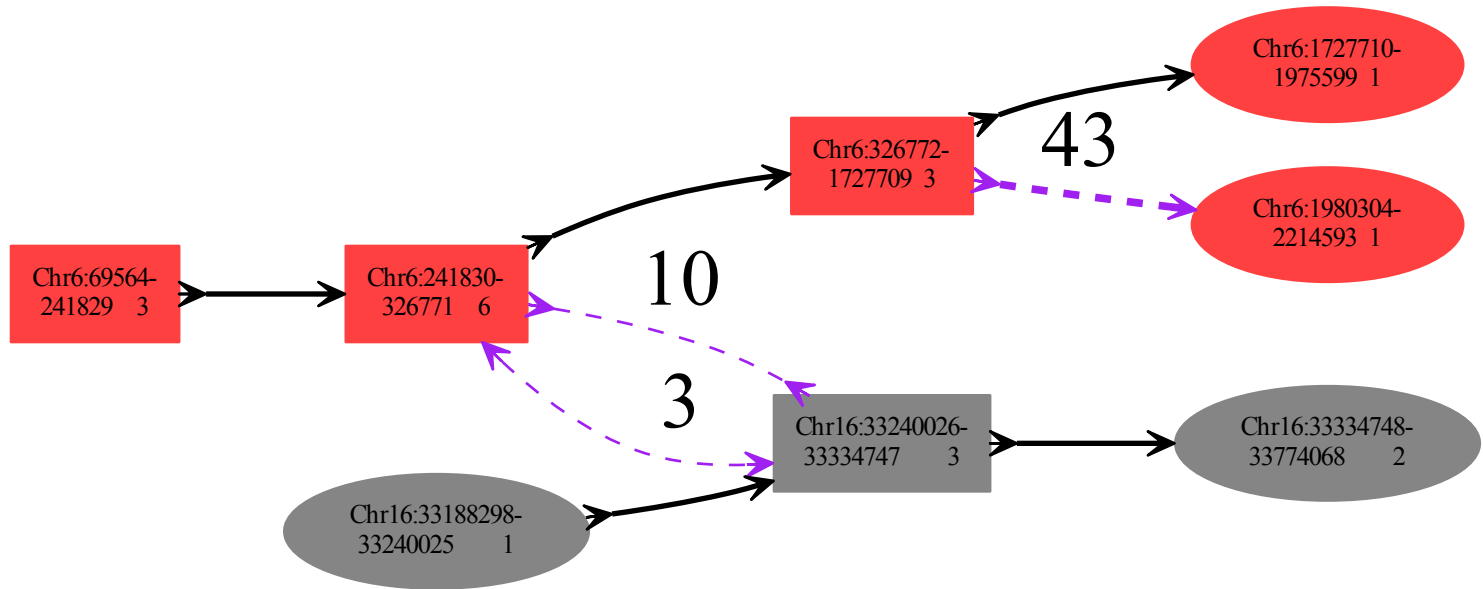

Supplement: Appendix S2 — Reconstruction of HCT116 genome structure by fusion point guided concatenation method. (ZIP) [file pone.0046152.s018.zip › subgraph30.pdf]

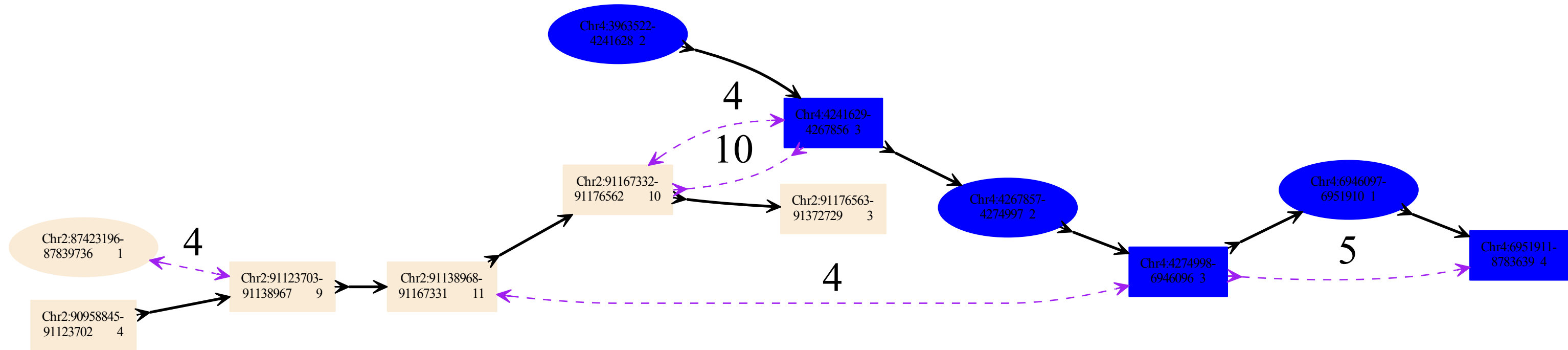

Supplement: Appendix S2 — Reconstruction of HCT116 genome structure by fusion point guided concatenation method. (ZIP) [file pone.0046152.s018.zip › subgraph31.pdf]

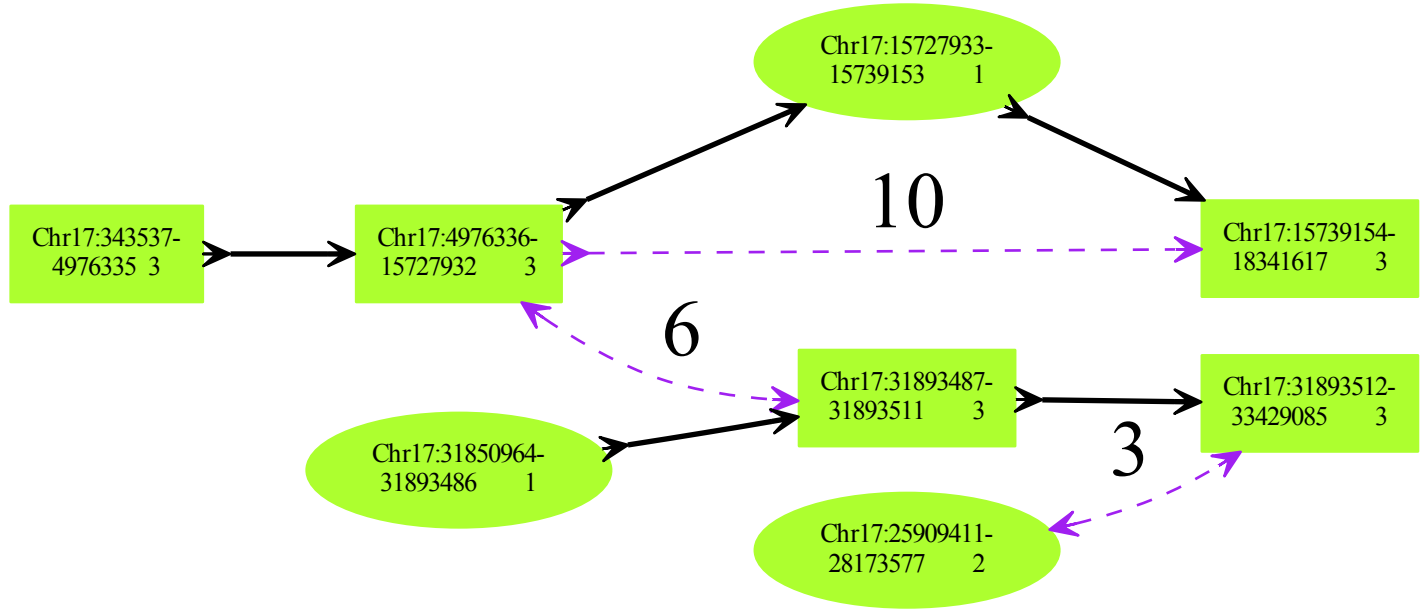

Supplement: Appendix S2 — Reconstruction of HCT116 genome structure by fusion point guided concatenation method. (ZIP) [file pone.0046152.s018.zip › subgraph32.pdf]

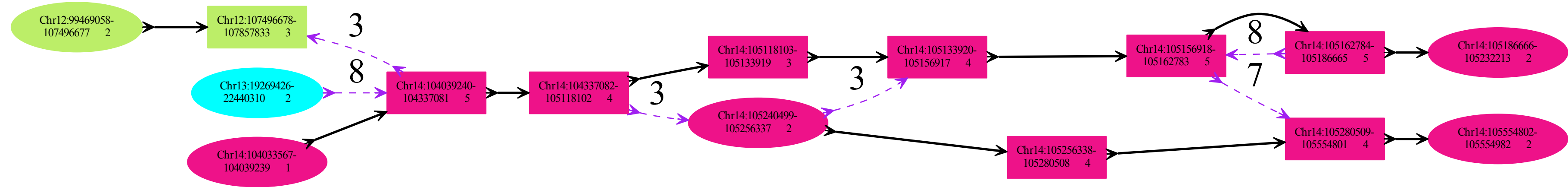

Supplement: Appendix S2 — Reconstruction of HCT116 genome structure by fusion point guided concatenation method. (ZIP) [file pone.0046152.s018.zip › subgraph33.pdf]

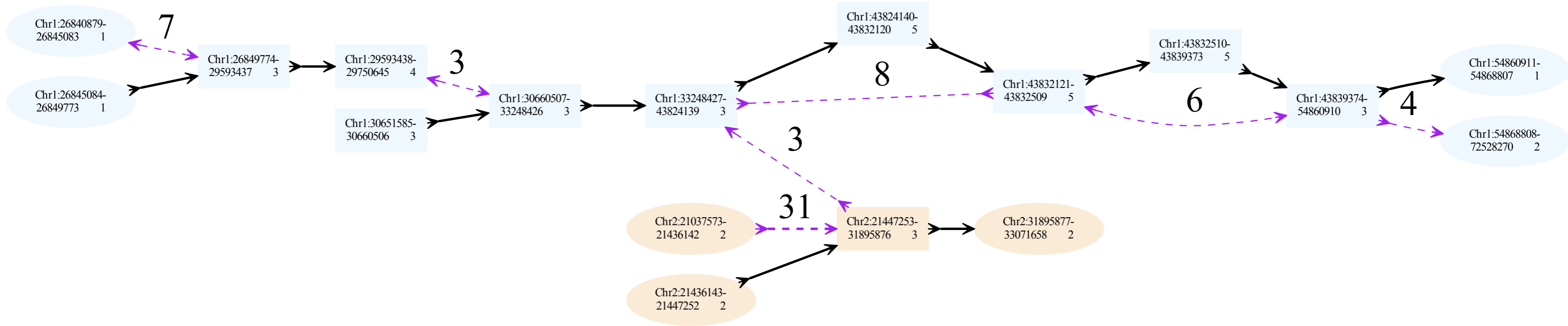

Supplement: Appendix S2 — Reconstruction of HCT116 genome structure by fusion point guided concatenation method. (ZIP) [file pone.0046152.s018.zip › subgraph34.pdf]

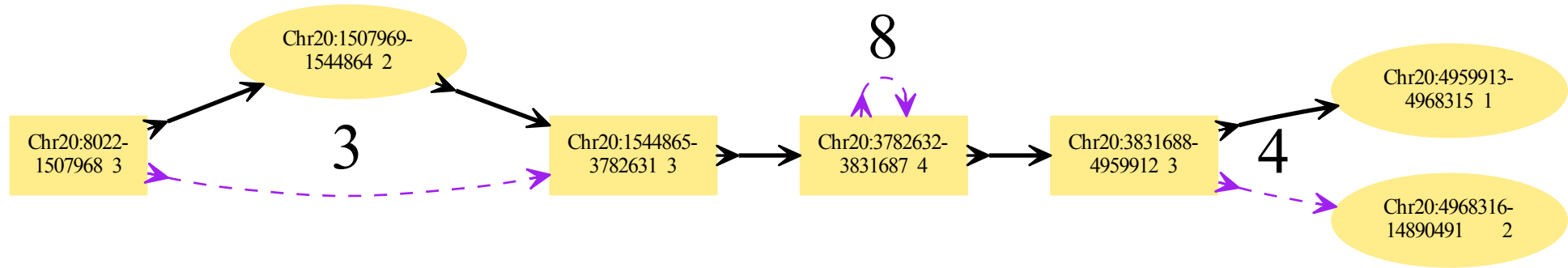

Supplement: Appendix S2 — Reconstruction of HCT116 genome structure by fusion point guided concatenation method. (ZIP) [file pone.0046152.s018.zip › subgraph35.pdf]

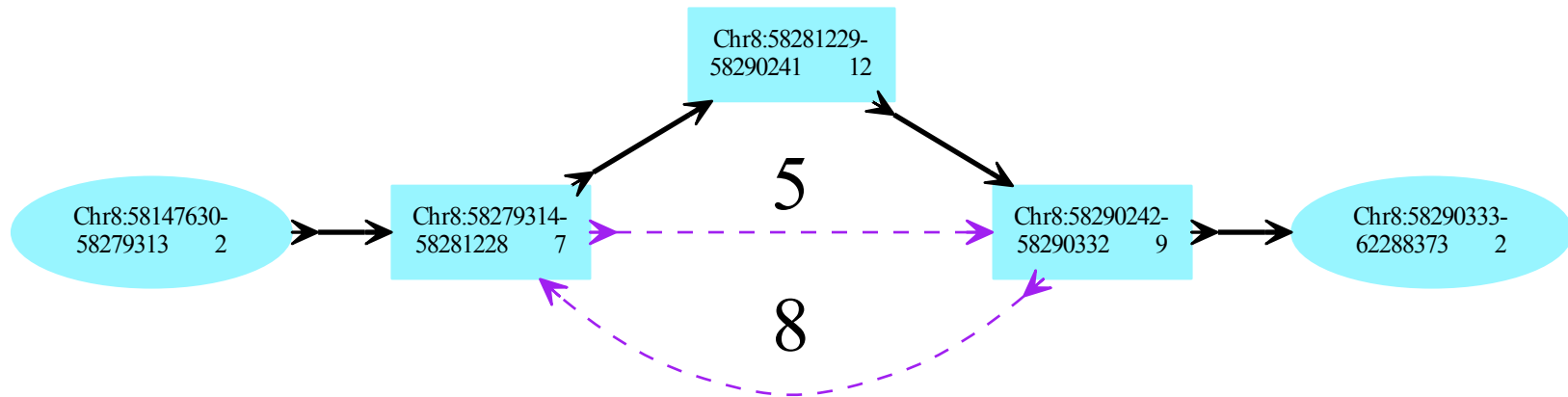

Supplement: Appendix S2 — Reconstruction of HCT116 genome structure by fusion point guided concatenation method. (ZIP) [file pone.0046152.s018.zip › subgraph36.pdf]

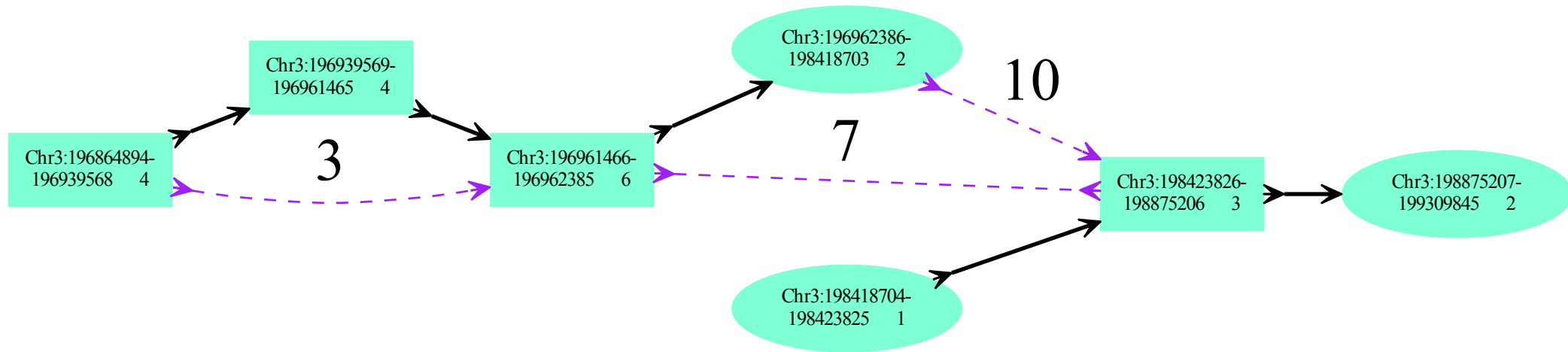

Supplement: Appendix S2 — Reconstruction of HCT116 genome structure by fusion point guided concatenation method. (ZIP) [file pone.0046152.s018.zip › subgraph37.pdf]

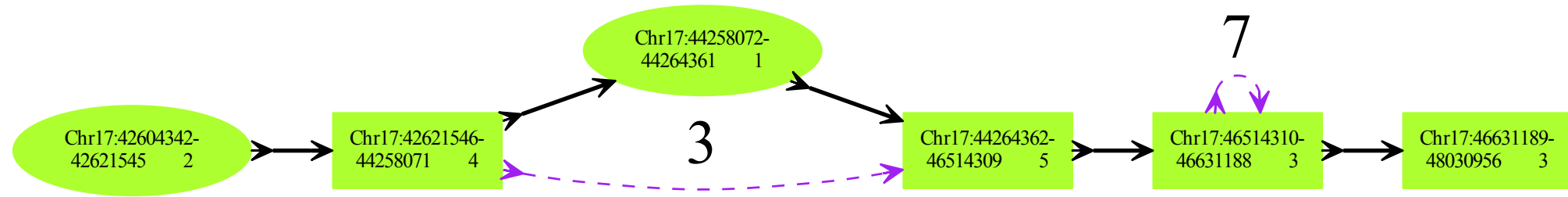

Supplement: Appendix S2 — Reconstruction of HCT116 genome structure by fusion point guided concatenation method. (ZIP) [file pone.0046152.s018.zip › subgraph38.pdf]

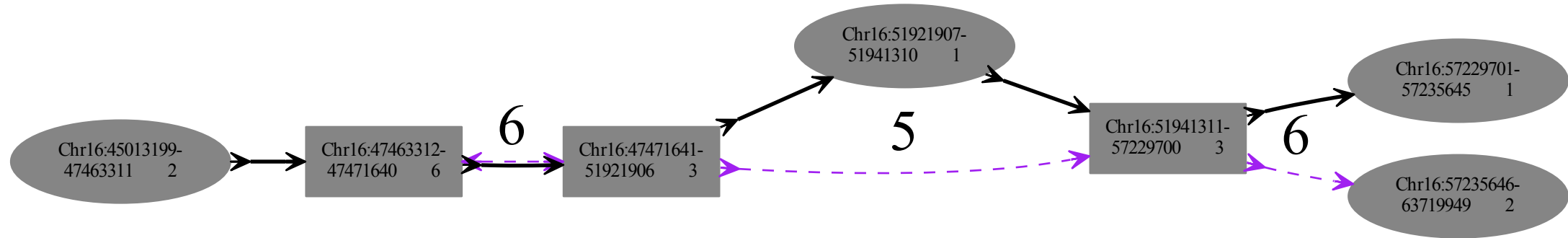

Supplement: Appendix S2 — Reconstruction of HCT116 genome structure by fusion point guided concatenation method. (ZIP) [file pone.0046152.s018.zip › subgraph39.pdf]

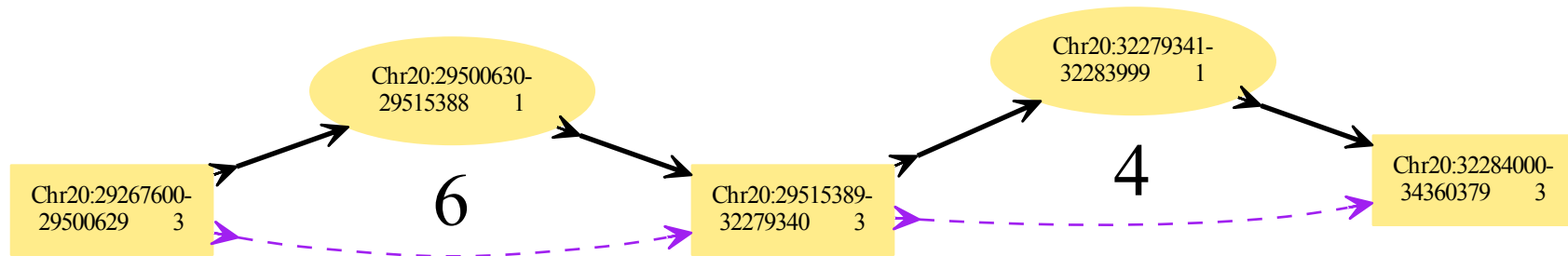

Supplement: Appendix S2 — Reconstruction of HCT116 genome structure by fusion point guided concatenation method. (ZIP) [file pone.0046152.s018.zip › subgraph40.pdf]

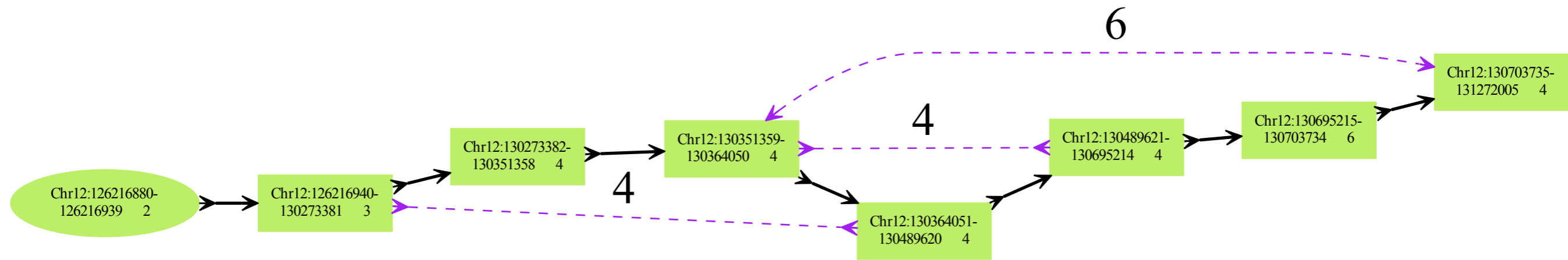

Supplement: Appendix S2 — Reconstruction of HCT116 genome structure by fusion point guided concatenation method. (ZIP) [file pone.0046152.s018.zip › subgraph41.pdf]

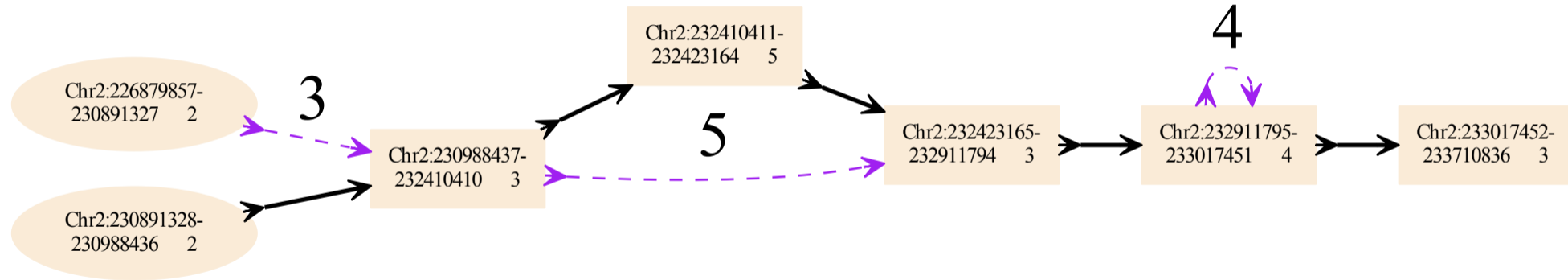

Supplement: Appendix S2 — Reconstruction of HCT116 genome structure by fusion point guided concatenation method. (ZIP) [file pone.0046152.s018.zip › subgraph42.pdf]

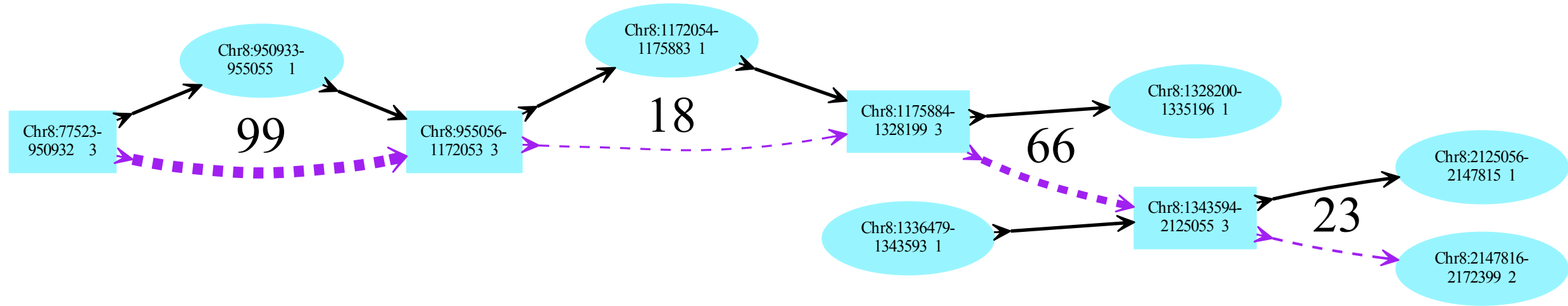

Supplement: Appendix S3 — Reconstruction of K562 genome structure by fusion point guided concatenation method. (ZIP) [file pone.0046152.s019.zip › subgraph10.pdf]

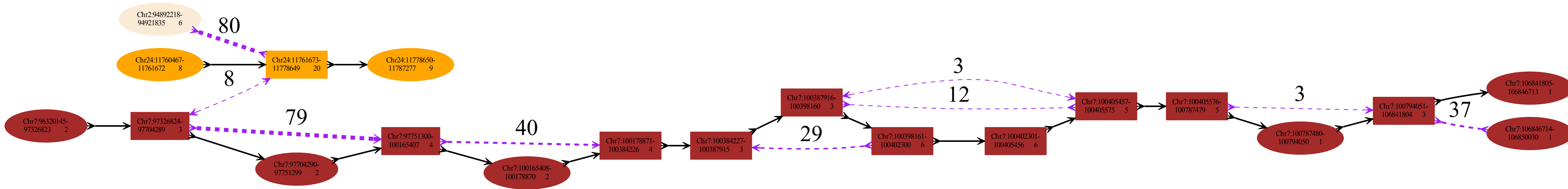

Supplement: Appendix S3 — Reconstruction of K562 genome structure by fusion point guided concatenation method. (ZIP) [file pone.0046152.s019.zip › subgraph11.pdf]

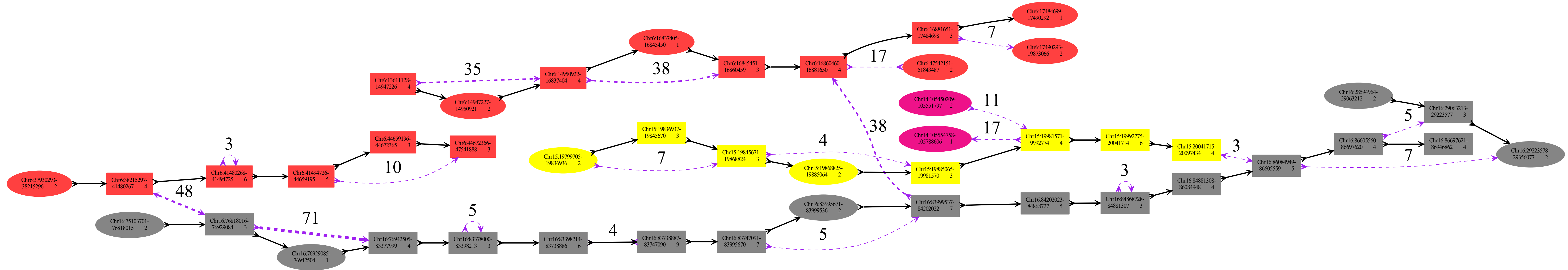

Supplement: Appendix S3 — Reconstruction of K562 genome structure by fusion point guided concatenation method. (ZIP) [file pone.0046152.s019.zip › subgraph12.pdf]

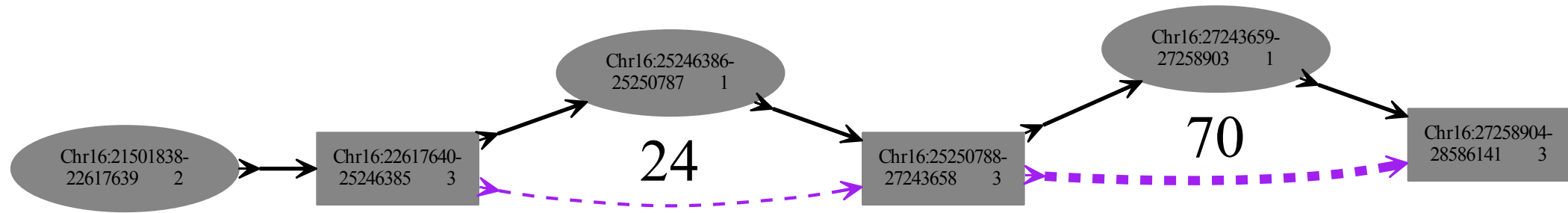

Supplement: Appendix S3 — Reconstruction of K562 genome structure by fusion point guided concatenation method. (ZIP) [file pone.0046152.s019.zip › subgraph13.pdf]

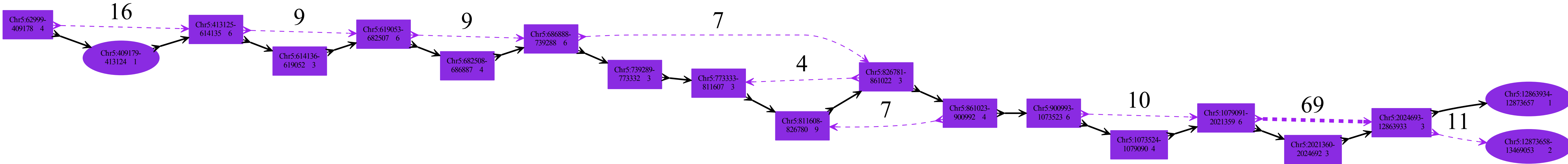

Supplement: Appendix S3 — Reconstruction of K562 genome structure by fusion point guided concatenation method. (ZIP) [file pone.0046152.s019.zip › subgraph14.pdf]

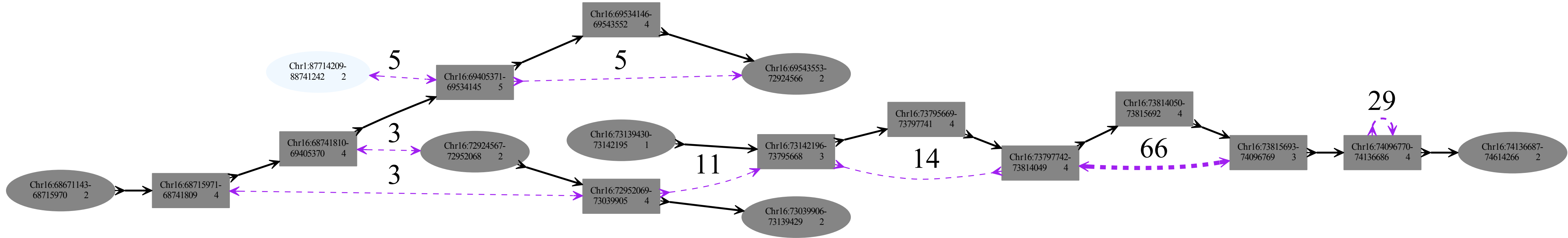

Supplement: Appendix S3 — Reconstruction of K562 genome structure by fusion point guided concatenation method. (ZIP) [file pone.0046152.s019.zip › subgraph15.pdf]

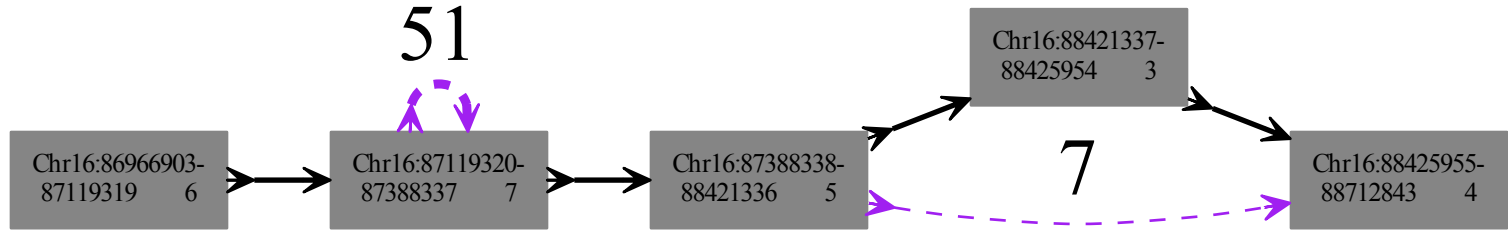

Supplement: Appendix S3 — Reconstruction of K562 genome structure by fusion point guided concatenation method. (ZIP) [file pone.0046152.s019.zip › subgraph16.pdf]

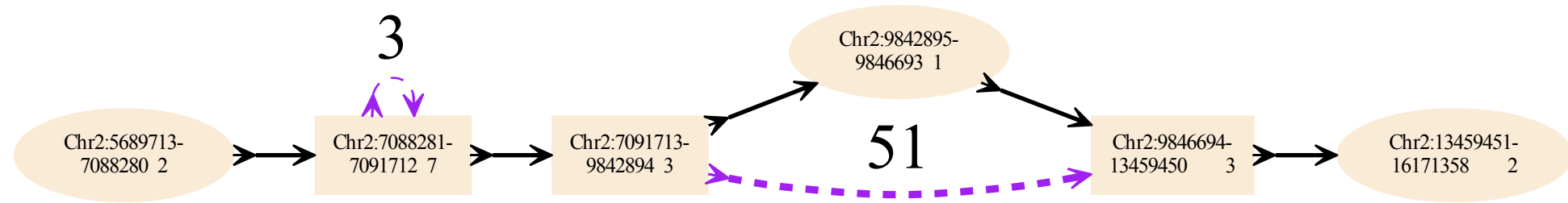

Supplement: Appendix S3 — Reconstruction of K562 genome structure by fusion point guided concatenation method. (ZIP) [file pone.0046152.s019.zip › subgraph17.pdf]

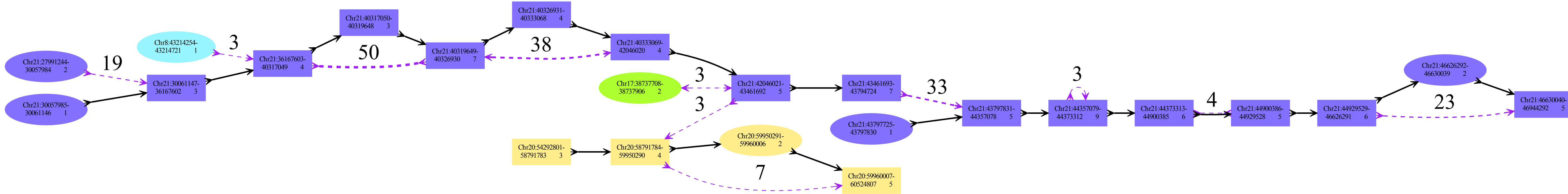

Supplement: Appendix S3 — Reconstruction of K562 genome structure by fusion point guided concatenation method. (ZIP) [file pone.0046152.s019.zip › subgraph18.pdf]
