# Supplementary material for: Long Span DNA Paired-End-Tag (DNA-PET) Sequencing Strategy for the Interrogation of Genomic Structural Mutations and Fusion-Point-Guided Reconstruction of Amplicons
Source: PLoS One. 2012 Sep 28;7(9):e46152. doi: 10.1371/journal.pone.0046152 (PMC3461012; doi:10.1371/journal.pone.0046152)
Supplement: Protocol S1 — Long Span DNA-PET Library Construction by EcoP15I. (DOC) [file pone.0046152.s016.doc]

**Overview**

The purpose of this procedure is to construct a mate-paired library. A mate-paired library consists of pairs of DNA fragments that are “mates” because they originate from the two ends of the same genomic DNA fragment. EcoP15I CAP adaptors connect the DNA mate-pairs together through an internal adaptor. The EcoP15I restriction enzyme sites in the genomic DNA are methylated prior to EcoP15I CAP adaptor ligation to ensure that only the unmethylated enzyme recognition sites in the CAP adaptor are recognized by EcoP15I during the restriction enzyme digestion step. As a result, EcoP15I cleaves 25-27 bp away from the unmethylated enzyme recognition sites in the CAP linker, yielding mate-paired genomic DNA attached to either side of the internal adaptor. P1 (ds) and P2 (ds) Adaptors are then ligated to the ends of the mate-paired library for subsequent amplification by PCR and enrichment of amplified template.

**Workflow:**

***Adapted from SOLiD^TM^ System Mate-Paired Library Preparation 2007***

**Material required**

Refer to *Appendix A*

**Procedures**

**Shear the DNA to Appropriate Size Using Hydroshear**

(1) Incubate the genomic DNA in 1 x TE buffer (pH 8.0) for 30 min at 37 ℃; vortex every 10 mins during incubation.

**This step is very important, especially when the genomic DNA is stored at low temperature. The hydroshear assembly is very easy to be clogged if there is some precipitation in your DNA sample.*

(2) Immediately before shearing, centrifuge the sample at a speed of 13,200 rpm at room temperature for 20 min.

(3) Transfer the supernatant by pipette into a new tube and discard the pellet.

(4) Check the concentration of genomic DNA by nanodrop.

(5) Shear the DNA using suitable shearing assembly at suitable speed code. Here are some examples:

| Fragment size | DNA concentration | Volume | Speed Code | Assembly |
| --- | --- | --- | --- | --- |
| 1kb | 150ng/µl | 150µl | 5 | Standard |
| 10kb | 250-300ng/µl | 250µl | 9 | Large |
| 20kb | 250-300ng/µl | 250µl | 11 | Large |

(6) Take out 500ng unsheared and sheared DNA to run agarose gel (70v, 1hr) to check the shearing quality.

(7) If the shearing size is right, do phenol/chloroform extraction and ethanol precipitation to purify DNA and re-suspend in 100µl nuclease free water.

1 2 3 4 5


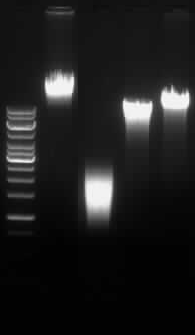


1 Generuler 1 kb DNA ladder

2 Un-sheared genomic DNA

3 Sheared genomic DNA to 1 kb

4 Sheared genomic DNA to 10 kb

5 Sheared genomic DNA to 20 kb

**Methylation of EcoP15I sites**

(1) Prepare the mixture:

100µl sheared DNA (30µg)

20µl 10x NEB Buffer 3

2µl 100x BSA

3µl EcoP15I enzyme 10unit/µl (final concentration of 10unit/µg)

2.5 µl 32mM SAM (final concentration of 400µM)

27.5µl H_2_O

200µl Total

-Incubate at 37℃ for 4hr or overnight (Both of them work very well).

(2) Phenol/chloroform extraction and ethanol precipitation, re-suspend in 30µl nuclease free water

(3) EcoP15I digestion to check the methylation quality

1 µl methylated sheared DNA (1µg)

20 µl 10x NEB Buffer 3

2 µl 100× BSA

40 µl 10×ATP (2 fold)

20 µl 1mM Sinefungin

2 µl EcoP15I enzyme 10unit/µl (final concentration of 10unit/µg)

115µl H_2_O

200µl Total

-Incubate at 37℃ for 2 hr

(4) Phenol/chloroform extraction and ethanol precipitation, re-suspend in 10µl EB. Add 2µl 6x loading dye.

(5) Run 0.5% agarose gel (70v, 1hr)

1 2 3 4 5


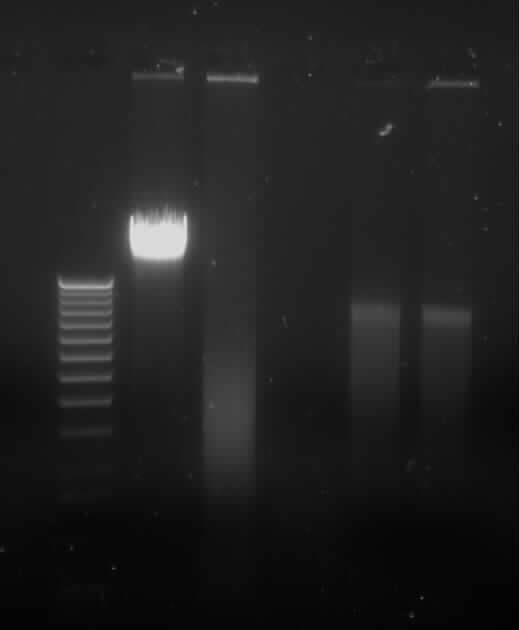


1 1 kb plus ladder (Invitrogen)

2 Un-sheared genomic DNA 280ng

3 EcoP15 I digested un-sheared genomic DNA

4 Methylated 10Kb genomic DNA

5 EcoP15 I digested methylated 10 kb genomic DNA

** Must use the un-methylated DNA as EcoP15I digestion control, otherwise you will not know your methylation is working or your EcoP15I digestion is not working.*

**End-polish and Ecop15I CAP linker ligation**

(1) Prepare the mixture:

30µl methylated DNA (30µg)

10µl 10x Epicentre Endit Buffer

10µl Epicentre Endit ATP

10µl Endit dNTPs

6µl Endit Enzyme mix

34µl H_2_O

100µl Total

-Incubate at 25℃ for 40 min

(2) Phenol/chloroform extraction and ethanol precipitation, re-suspend in 100µl nuclease free water.

(3) Calculate the cap linker to be used by this forum:

1µg of 1kb DNA = 1.52µmols

1µg of 10kb DNA = 0.152µmols

30µg of 10kb DNA = 4.56µmols

4.56µmols ×200/50pmol/µl of EcoP15 I CAP Linker = 18.24µl

(4) Prepare the mixture:

100 µl 10kb polished and methylated DNA

19 µl EcoP15I CAP Linkers (50 pmol/µl)

80 µl 5x Invitrogen ligase buffer

2 µl Fermentas High concentration ligase (30unit/µl)

199 µl H_2_O

400µl Total

-Incubate at 16℃ overnight

(5) Phenol/chloroform extraction and ethanol precipitation, re-suspend in 100µl EB buffer.

**Gel extraction**

1. Add 20µl 6x loading dye

-10 kb and 20 kb DNA: Run 0.5% agarose gel with two normal sized slots for markers and one large merged slot for the sample at 80v for 10min and 20v for 20hr at room temperature with Generuler 1 kb ladder and Invitrogen 1 kb plus ladder for 10 kb library; Generuler High Range ladder for 20 kb library.

-1 kb DNA: Run 1% agarose gel at 60V for 6hrs at room temperature with Invitrogen 100bp ladder.

(2) Staining gel with SYBR Gold for 1hr (200 ml 1xTAE buffer with 50 µl SYBR Gold).

(3) Elute DNA fragment from gel

10 kb and 20 kb : Elute DNA by QIAEX II Gel Extraction Kit, the usual cutting range is 8-9 kb, 9-10 kb, 10-11 kb and 11-12 kb for 10 kb library; 15-18 kb, 18-20 kb, 20-23 kb for 20 kb library.

1 kb: Extract DNA by QIAquick Gel Extraction Kit. The usual cutting range is 1.2-1.3kb,

1.3-1.4kb and 1.4-1.5kb.


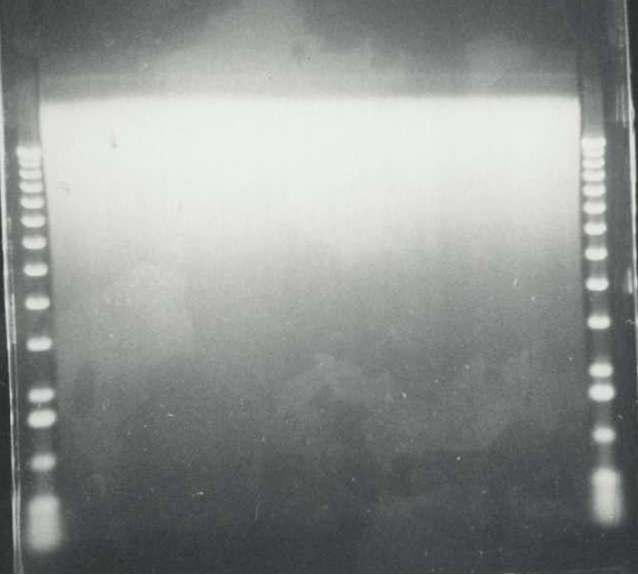


10Kb

(4) Quantitate the DNA by Agilent DNA 12000 Kit if the DNA fragment is smaller than 11 kb or picogreen if the DNA fragment is larger than 11 kb (the maximum size of Agilent DNA 12000 Kit is 12 kb). Here is one example.


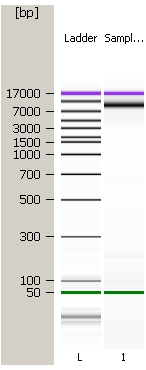

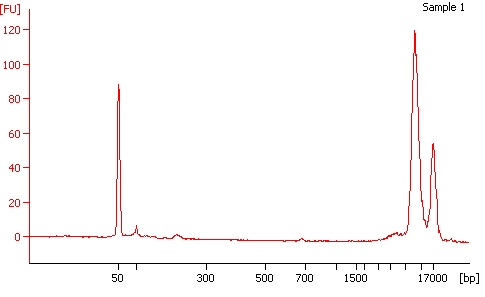


| Size [bp] | Conc. [ng/µl] | Molarity [nmol/l] |
| --- | --- | --- |
| 50 | 8.3 | 251.5 |
| 9,033 | 11.07 | 1.9 |
| 17,000 | 4.2 | 0.4 |

**Circularization to bring the 2 ends of the sheared molecule together**

(1) Calculate the amount of Sticky internal linker:

1µg of 1kb DNA=1.52pmol

1µg of 10kb DNA = 0.152pmol

If you get 500ng of 10kb DNA after gel purification:

0.5µg ×0.152pmol ×5/2pmol= 0.19µl of Sticky internal linker

**It is better to dilute the internal linker to 0.2pmol and add 1.9µl.*

1. Prepare the mixture:

100 µl 10kb fragment (final concentration 0.1ng/µl)

660 µl Invitrogen 5x ligase buffer

3.3 µl Sticky internal adaptor (1ng/µl)

66 µl Fermentas ligase (5unit/µl, final concentration 0.1unit/µl )

2560.7 µl H_2_O

5000µl Total

-Incubate at 16℃ overnight.

(3) Phenol/chloroform extraction and isopropanol precipitation, re-suspend in 40 µl nuclease free water.

**Plasmid safe and EcoP15I digestion**

1. Prepare the mixture:

40 µl Circularized DNA

5 µl 10x Buffer

2 µl 25mM ATP (final 1mM)

0.4 µl DNase (10unit/µg)

2.6 µl H_2_O

50 µl Total

-Incubate at 37℃ for 40 min

(2) Phenol/chloroform extraction and ethanol precipitation, re-suspend in 134.3µl nuclease free water.

1. Prepare the mixture:

134.3 µl Circularized DNA

20 µl 10x NEB buffer 3

2 µl 100x BSA

2 µl 10mM Sinefungin

40 µl 10x ATP

1.7 µl EcoP15I (2unit/µl)

200 µl Total

-Incubate at 37℃ for 2hr

(4) Phenol/chloroform extraction and ethanol precipitation, re-suspend in 20µl nuclease

free water.

**Sequencing adaptor ligation**

1. Prepare the mixture:

20 µl Digestion Product

5 µl 10x Epicentre Endit Buffer

5 µl Epicentre Endit ATP

5 µl Epicentre Endit dNTPs

1 µl Epicentre Endit Enzyme mix

14 µl H_2_O

50µl Total

-Incubate at 25℃ for 40min

(2) Phenol/chloroform extraction and ethanol precipitation, re-suspend in 100µl

nuclease free water.

(3) Shake the bottle of Dynal M280 streptavidin beads. Transfer 50ul of re-suspended Dynabeads to a 1.5 ml tube; with the help of MPC, wash the beads twice with 100ul of 2×B&W Buffer (10mM Tris-HCl pH7.5, 1mM EDTA, 2M NaCl); re-suspend beads in 100ul of 2×B&W Buffer.

(4) Add 100ul End Repaired EcoP15I digested product to the re-suspended beads, mix well. Incubate at RT with rotation on the Intelli-Mixer (Program F8, 30rpm) for 30 min. With the help of MPC, wash the beads twice with 100ul of 1×B&W Buffer (5mM Tris-HCl pH7.5, 0.5mM EDTA, 1M NaCl).

(5) Prepare the mixture:

4 µl P1 Adapter (200ng/ul)

4 µl P2 Adapter (200ng/ul)

10 µl 5× T4 DNA Ligase Buffer (Invitrogen)

1 µl T4 DNA Ligase (30u/ul, Fermentas)

31 µl H_2_O

50 µl Total

-Re-suspend the beads with the above ligation mix. Incubate at 16℃ with rotation on the Intelli-Mixer (Program F8, 30rpm) overnight.

**Trial PCR**

1. Wash the beads twice with 100ul of 1×B&W Buffer with the help of MPC.
2. Prepare the mixture:

5 µl 10×NEBuffer 2

2.5 µl 10mM dNTPs (500 µM in final conc.)

4 µl *E. coli* DNA Polymerase I (10u/ul, NEB)

38.5 µl H_2_O

50ul Total

-Re-suspend the beads with the above reaction mix. Incubate at RT with rotation on the Intelli-Mixer (Program F8, 30rpm) for 1h.

1. Wash the beads twice with 100ul of 1×B&W Buffer with the help of MPC. Re-suspend the beads in 50ul of EB buffer. Transfer to a fresh 1.5ml tube.
2. For each PCR reaction, in a 0.2ml PCR tube, add and mix:

2 µl Beads suspension

1 µl PCR primer 1 (10uM)

1 µl PCR primer 2 (10uM)

25 µl Phusion master mix HF (2x; Finnzymes)

21 µl H_2_O

50 µl Total

PCR program:

98°C, 30 s

98°C, 10 s }

55°C, 30 s } 16 cycles

72°C, 30 s }

72°C, 5 min

1. Transfer PCR products to a new 1.5 ml tube.
2. Using the MPC, move the beads aside and take out 25 µl of the PCR product to load on a 6% TBE PAGE gel.
3. Run at 200V, 40 min.
4. Stain with SYBR Green for 15 min.
5. View on Dark Reader to visualize the 154bp PETs.


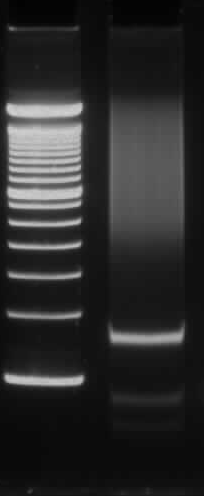


**154 bp PET**

**Large Scale amplification and purification of the PCR product**

(1) Repeat above step for another 24 PCR reactions

(2) Pool the PCR products. Remove the Dynabeads with the help of MPC

(3) Purify DNA by Isopropanol precipitation with GlycoBlue as co-precipitant and re-suspend the DNA pellet into 40ul nuclease free water.

(4) Run the 40ul DNA on the 6% TBE PAGE gel (Invitrogen), 200V, for 40 min together with 1µl of 25 bp Ladder (Invitrogen).

(5) Staining the gel with SYBR Green for 15min.


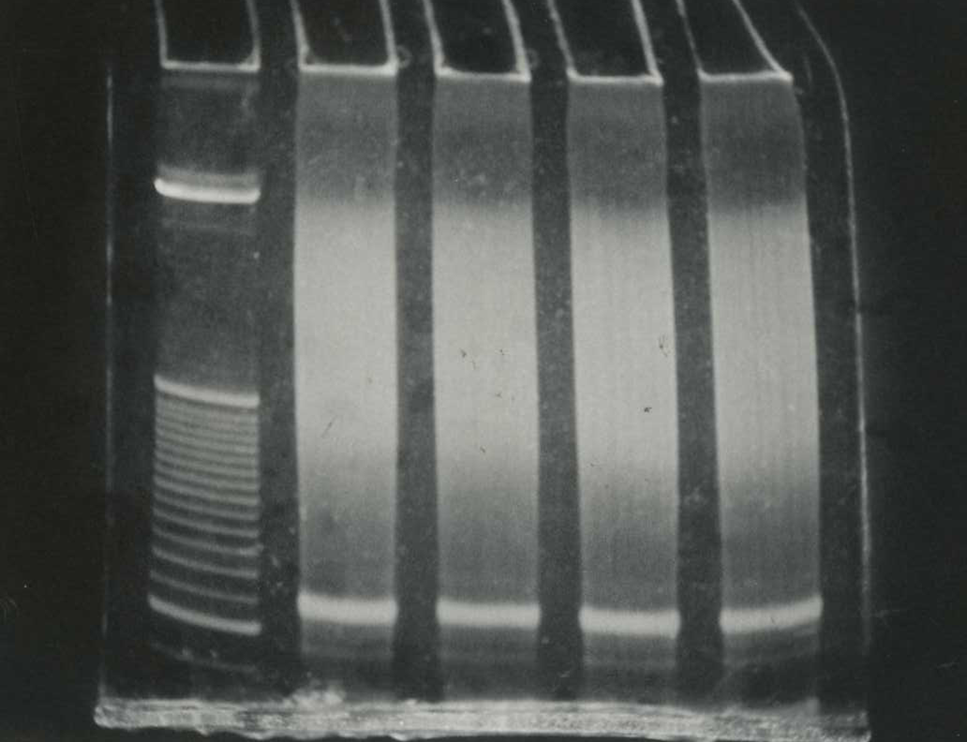


PET

(6) The PCR products with the size 154 bp are excised and place gel pieces in pierced 0.6ml tubes inserted in 1.5ml screw cap tubes.

(7) Centrifuge for 5 min at 13,200rpm, 4°C to shred gel.

(8) Add 200µl TE Buffer to each tube of shredded gel and freeze at -80°C for 1-2 hrs, then incubate at 37°C overnight.

(9) Transfer shredded gel & buffer to spin filter columns and centrifuge for 10 min at 13,200rpm, 4°C to remove shredded gel from buffer.

(10) Purify DNA by phenol/chloroform extraction and isopropanol precipitation (with GlycoBlue).

(11) Resuspend DNA in 20µl Buffer EB and Quantitate by Agilent DNA 1000.


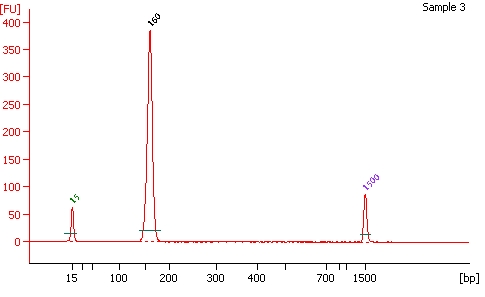

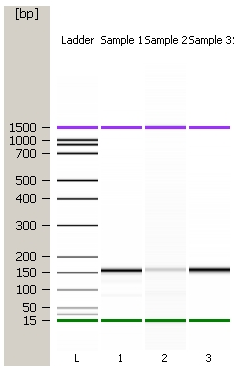


| Size [bp] | Conc. [ng/µl] | Molarity [nmol/l] |
| --- | --- | --- |
| 15 | 4.2 | 424.2 |
| 160 | 31.71 | 301 |
| 1,500 | 2.1 | 2.1 |

**Appendix A: Reagents and Solutions**

3M Sodium Acetate (Ambion)

5x T4 DNA Ligase Buffer (Invitrogen)

Agarose

Agilent DNA 1000 Kit

Agilent DNA 12000 Kit

Buffer EB (Qiagen)

Calf Thymus DNA (standard for picogreen)

DNA Ladder 100bp (Invitrogen)

DNA Ladder 1kb Plus (Invitrogen)

DNA Ladder 25bp (Invitrogen)

DNA Ladder Generuler 1kb (Fermentas)

DNA Ladder Generuler High Range (Fermentas)

Dynabeads M-280 Streptavidin (Invitrogen)

E.coli DNA Polymerase I (10u/ul, NEB)

EcoP15 I (10u/ul, NEB)

End-It DNA End-Repair Kit (Epicentre)

Ethanol

Ethidium Bromide

Fujifilm FP-3000B

Gel Handler Gel Support

GlycoBlue (Ambion)

Isopropanol

Iwaki 96-well flat bottom plate

MaXtract High Density (200 x 2 ml), Qiagen

MaXtract High Density (25 x 50 ml), Qiagen

Novex pre-cast 6% TBE PAGE gel

Nuclease-free water

Phenol/chloroform/IAA (Ambion)

Phusion Mastermix (Finnzymes)

PicoGreen (Invitrogen)

Plasmid Safe DNase (Epicentre)

QIAEXII Gel Extraction kit (Qiagen)

SAM (32mM, NEB)

Sinefungin (10mM, Calbiochem)

Spin Module and Recovery Tube

SYBR Green I (Invitrogen)

T4 DNA Ligase (30u/ul, Fermentas)

TAE buffer

TBE buffer

TE buffer
