# Supplementary figures and images for: Long Span DNA Paired-End-Tag (DNA-PET) Sequencing Strategy for the Interrogation of Genomic Structural Mutations and Fusion-Point-Guided Reconstruction of Amplicons (part 2 of 2)
Source: PLoS One. 2012 Sep 28;7(9):e46152. doi: 10.1371/journal.pone.0046152 (PMC3461012; doi:10.1371/journal.pone.0046152)

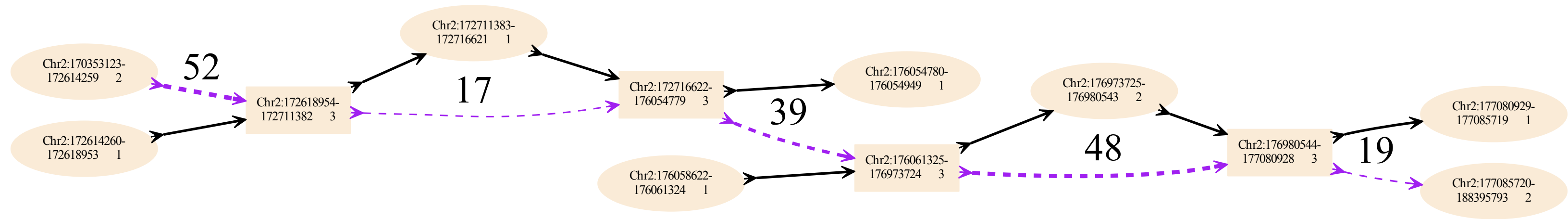

Supplement: Appendix S3 — Reconstruction of K562 genome structure by fusion point guided concatenation method. (ZIP) [file pone.0046152.s019.zip › subgraph19.pdf]

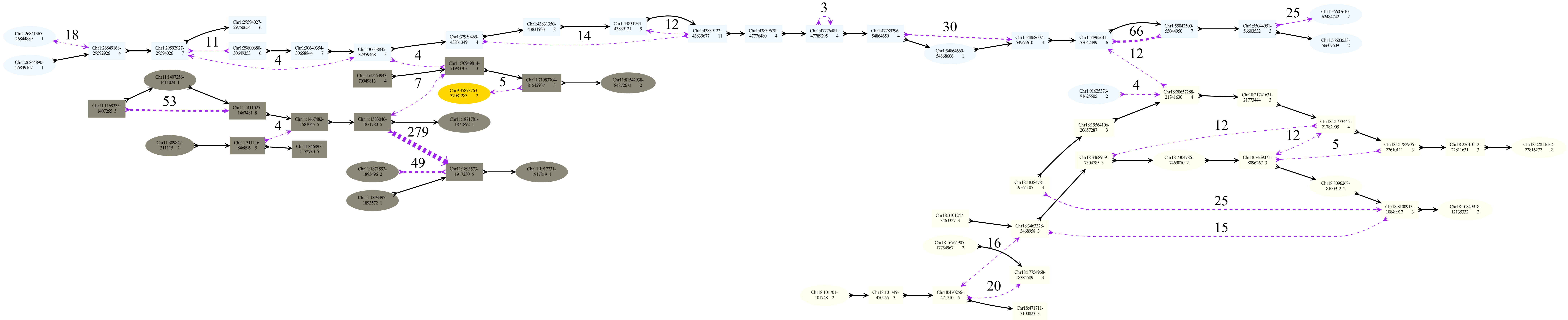

Supplement: Appendix S3 — Reconstruction of K562 genome structure by fusion point guided concatenation method. (ZIP) [file pone.0046152.s019.zip › subgraph2.pdf]

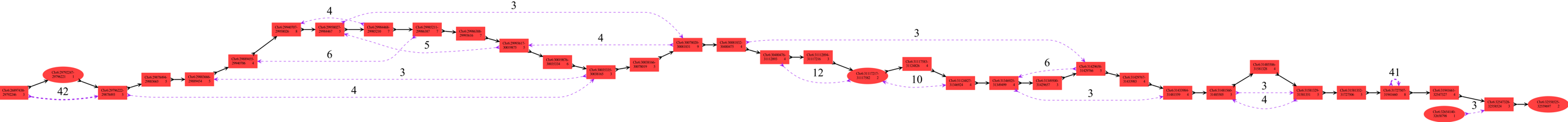

Supplement: Appendix S3 — Reconstruction of K562 genome structure by fusion point guided concatenation method. (ZIP) [file pone.0046152.s019.zip › subgraph20.pdf]

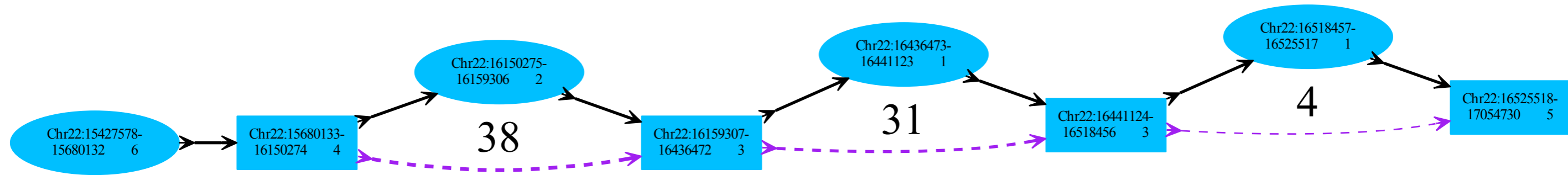

Supplement: Appendix S3 — Reconstruction of K562 genome structure by fusion point guided concatenation method. (ZIP) [file pone.0046152.s019.zip › subgraph21.pdf]

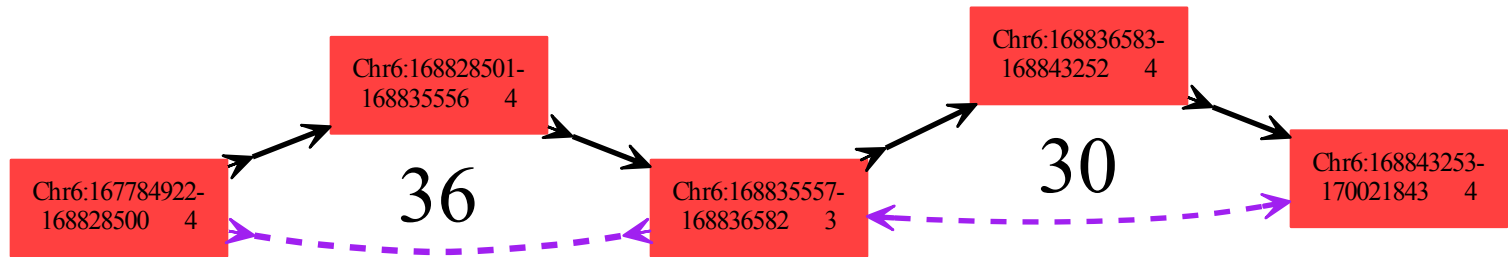

Supplement: Appendix S3 — Reconstruction of K562 genome structure by fusion point guided concatenation method. (ZIP) [file pone.0046152.s019.zip › subgraph22.pdf]

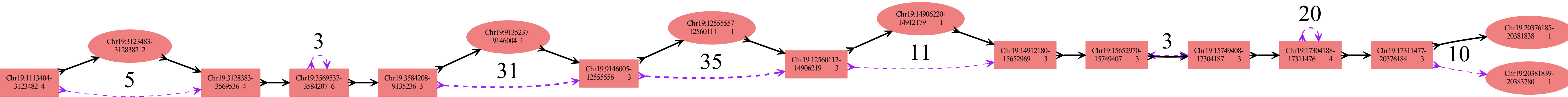

Supplement: Appendix S3 — Reconstruction of K562 genome structure by fusion point guided concatenation method. (ZIP) [file pone.0046152.s019.zip › subgraph23.pdf]

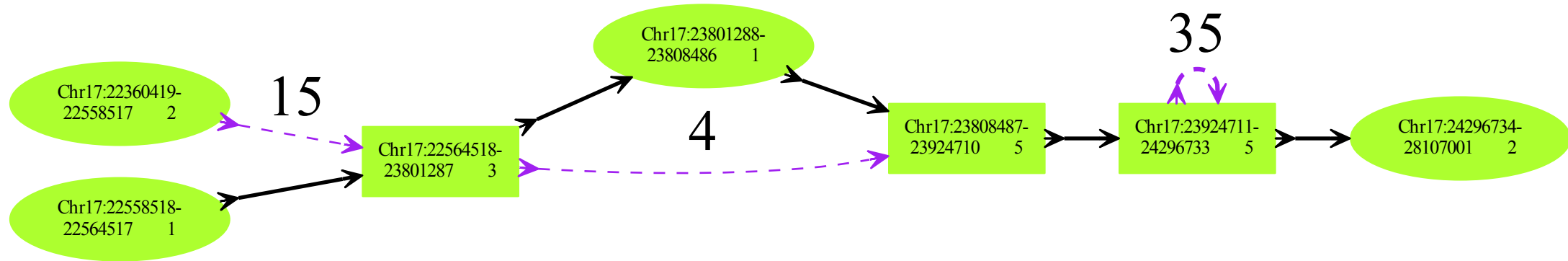

Supplement: Appendix S3 — Reconstruction of K562 genome structure by fusion point guided concatenation method. (ZIP) [file pone.0046152.s019.zip › subgraph24.pdf]

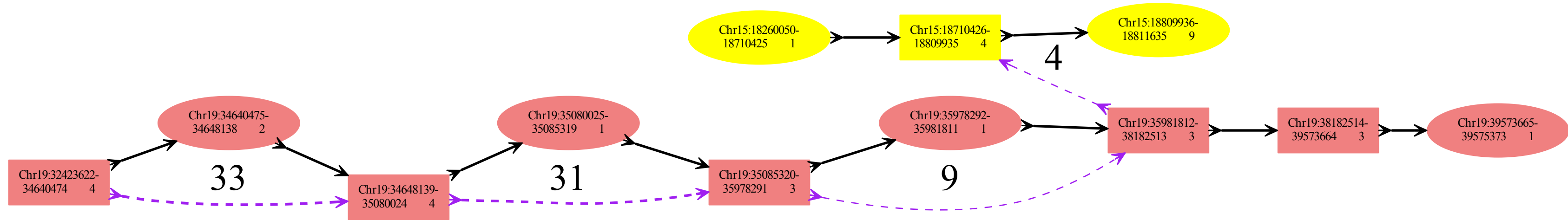

Supplement: Appendix S3 — Reconstruction of K562 genome structure by fusion point guided concatenation method. (ZIP) [file pone.0046152.s019.zip › subgraph25.pdf]

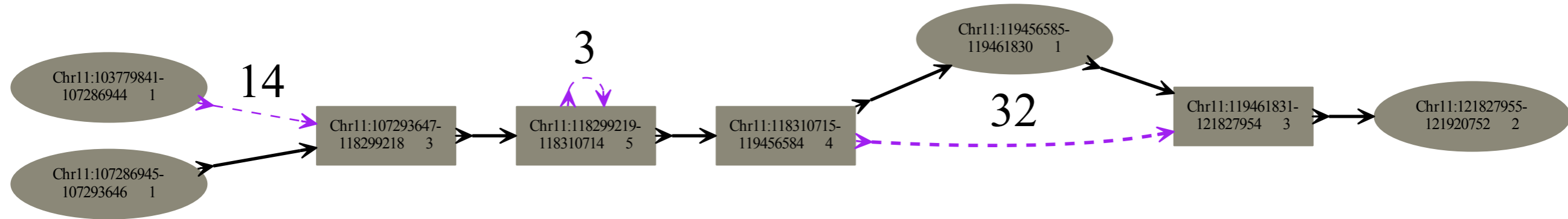

Supplement: Appendix S3 — Reconstruction of K562 genome structure by fusion point guided concatenation method. (ZIP) [file pone.0046152.s019.zip › subgraph26.pdf]

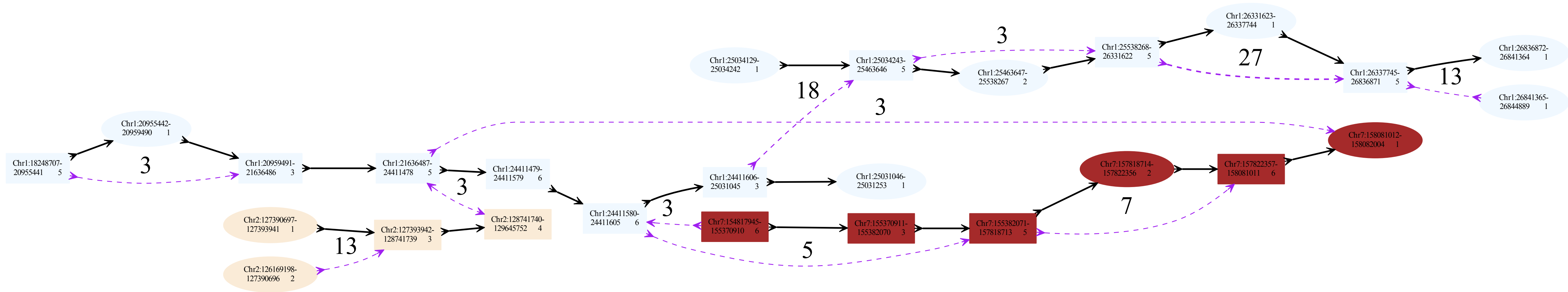

Supplement: Appendix S3 — Reconstruction of K562 genome structure by fusion point guided concatenation method. (ZIP) [file pone.0046152.s019.zip › subgraph27.pdf]

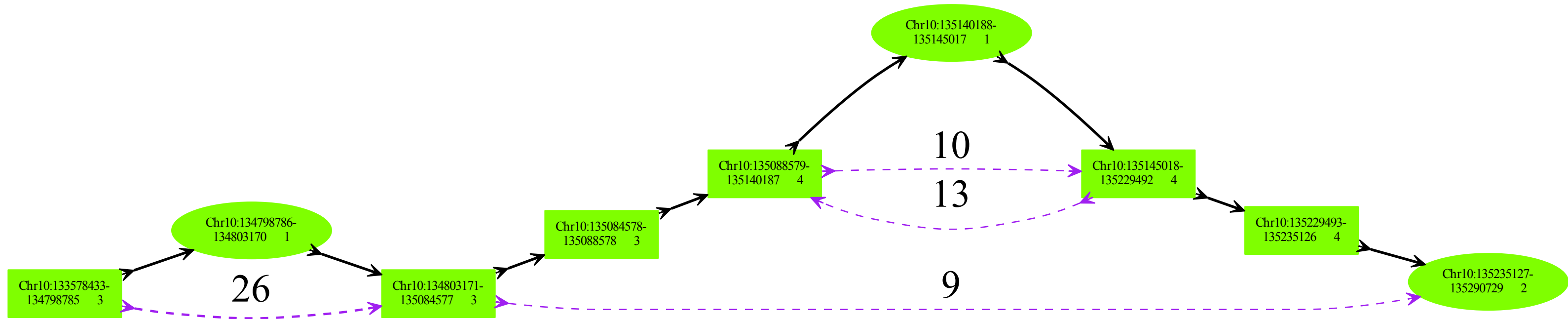

Supplement: Appendix S3 — Reconstruction of K562 genome structure by fusion point guided concatenation method. (ZIP) [file pone.0046152.s019.zip › subgraph28.pdf]

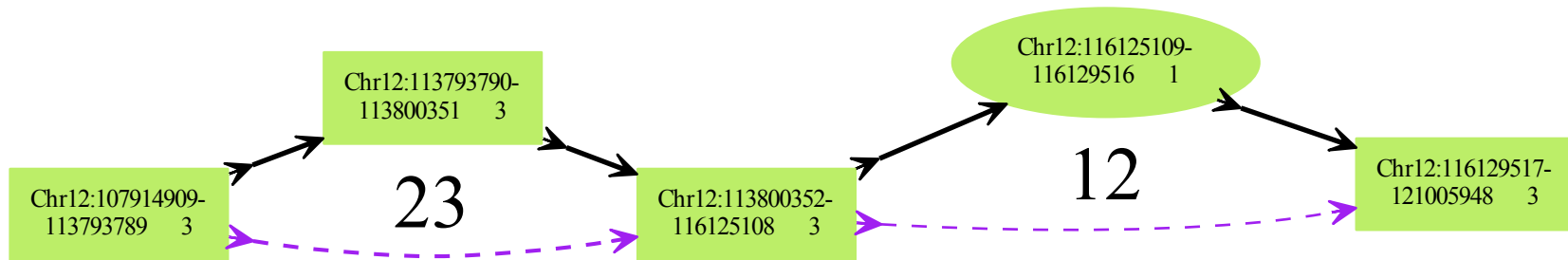

Supplement: Appendix S3 — Reconstruction of K562 genome structure by fusion point guided concatenation method. (ZIP) [file pone.0046152.s019.zip › subgraph29.pdf]

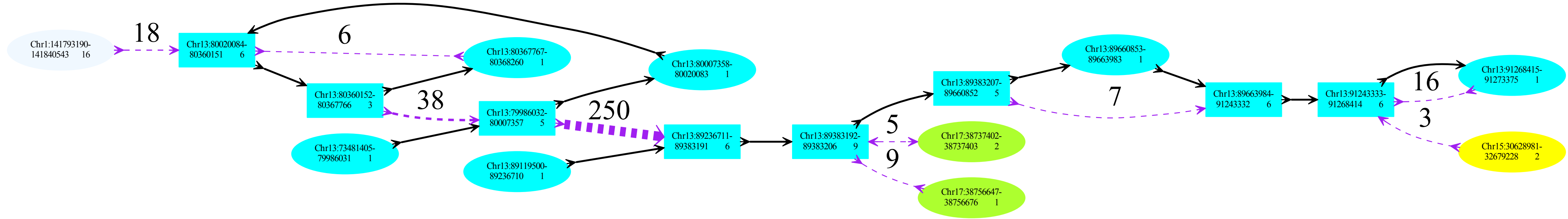

Supplement: Appendix S3 — Reconstruction of K562 genome structure by fusion point guided concatenation method. (ZIP) [file pone.0046152.s019.zip › subgraph3.pdf]

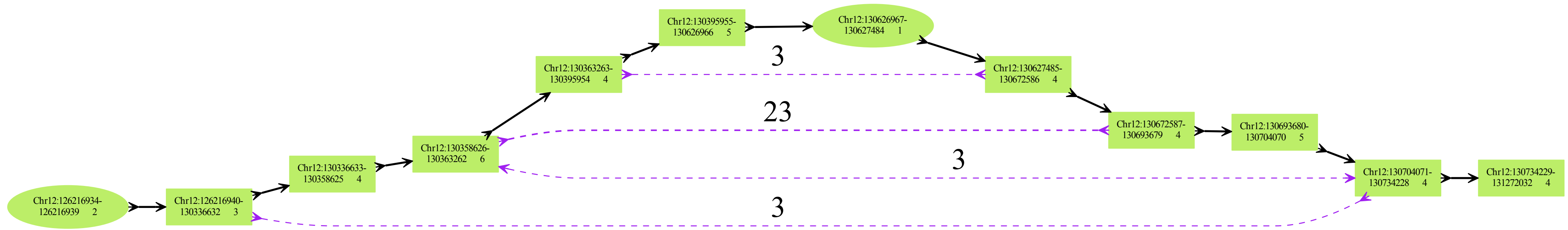

Supplement: Appendix S3 — Reconstruction of K562 genome structure by fusion point guided concatenation method. (ZIP) [file pone.0046152.s019.zip › subgraph30.pdf]

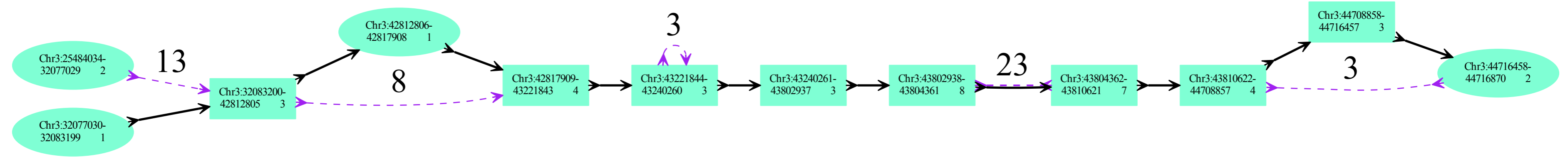

Supplement: Appendix S3 — Reconstruction of K562 genome structure by fusion point guided concatenation method. (ZIP) [file pone.0046152.s019.zip › subgraph31.pdf]

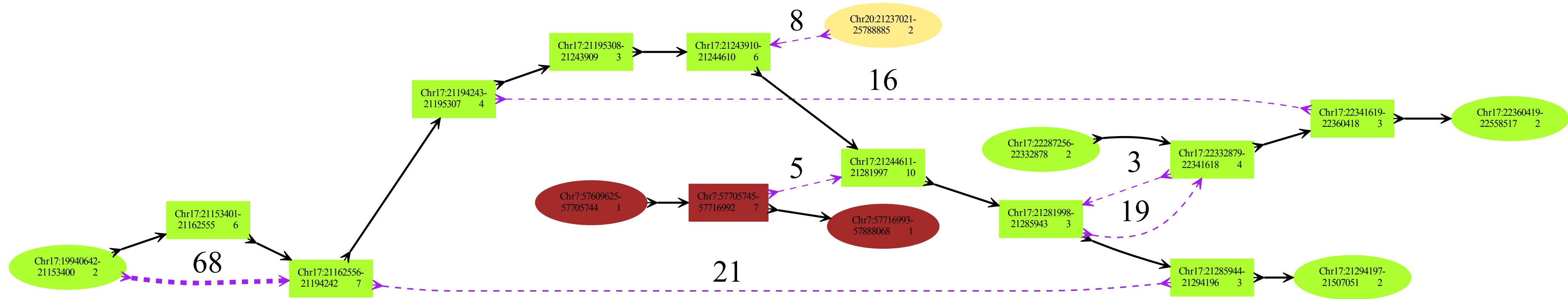

Supplement: Appendix S3 — Reconstruction of K562 genome structure by fusion point guided concatenation method. (ZIP) [file pone.0046152.s019.zip › subgraph32.pdf]

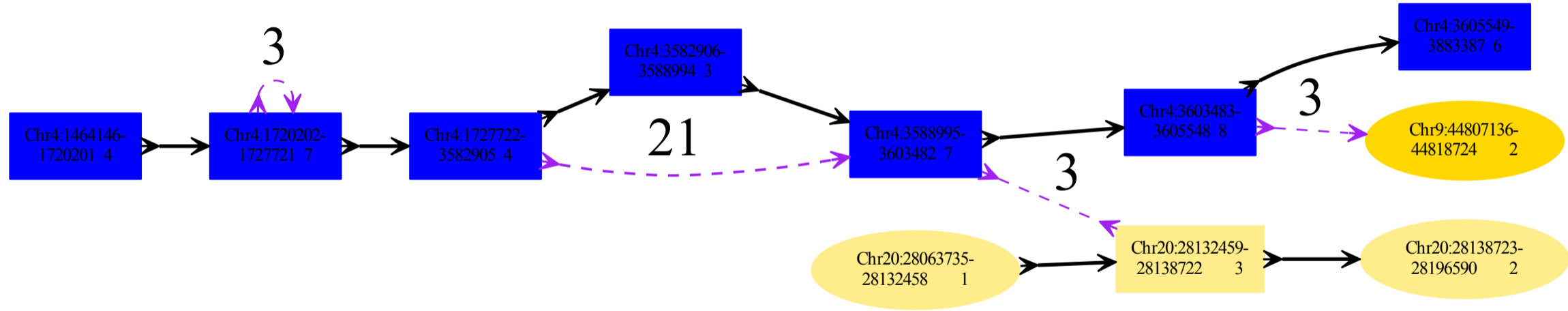

Supplement: Appendix S3 — Reconstruction of K562 genome structure by fusion point guided concatenation method. (ZIP) [file pone.0046152.s019.zip › subgraph33.pdf]

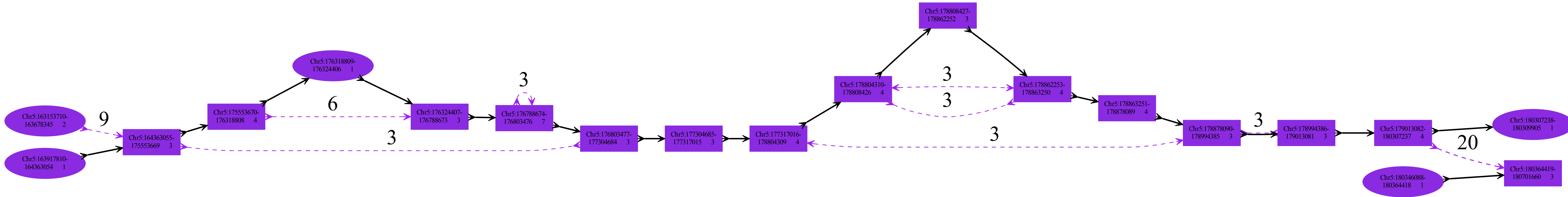

Supplement: Appendix S3 — Reconstruction of K562 genome structure by fusion point guided concatenation method. (ZIP) [file pone.0046152.s019.zip › subgraph34.pdf]

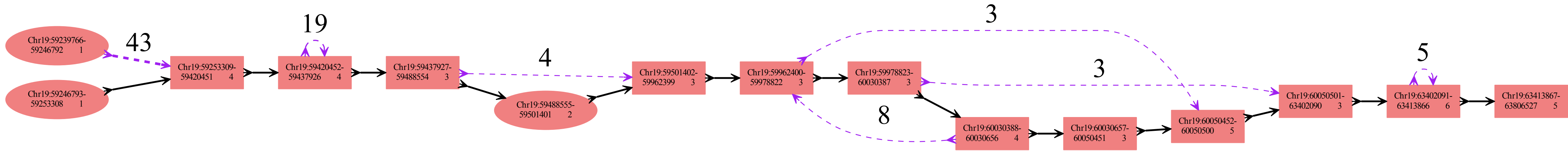

Supplement: Appendix S3 — Reconstruction of K562 genome structure by fusion point guided concatenation method. (ZIP) [file pone.0046152.s019.zip › subgraph35.pdf]

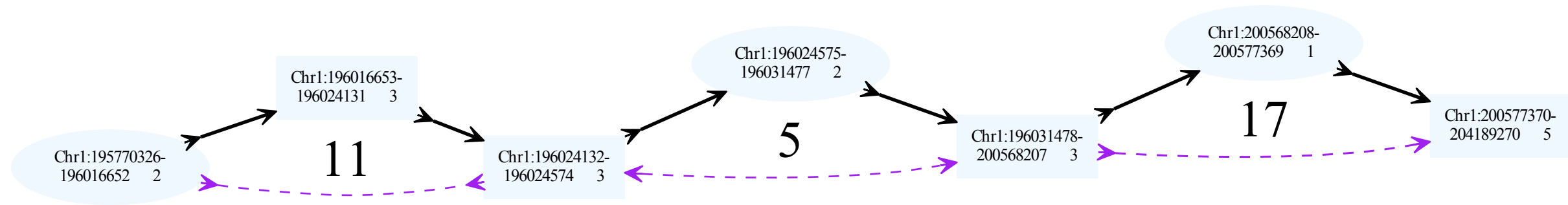

Supplement: Appendix S3 — Reconstruction of K562 genome structure by fusion point guided concatenation method. (ZIP) [file pone.0046152.s019.zip › subgraph36.pdf]

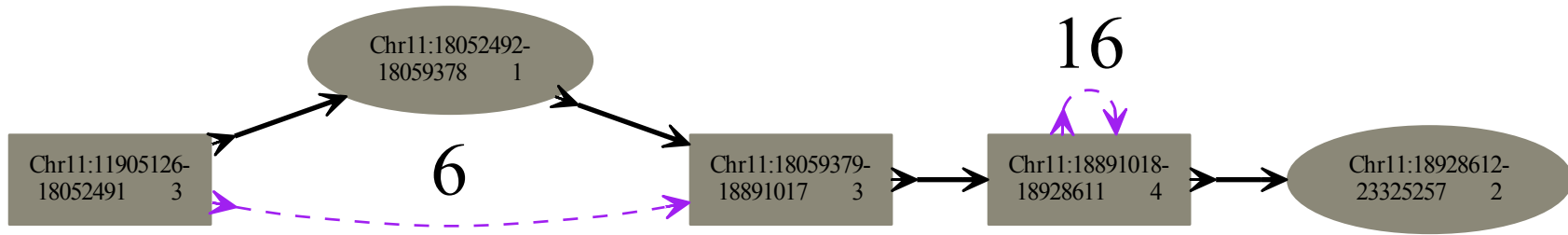

Supplement: Appendix S3 — Reconstruction of K562 genome structure by fusion point guided concatenation method. (ZIP) [file pone.0046152.s019.zip › subgraph37.pdf]

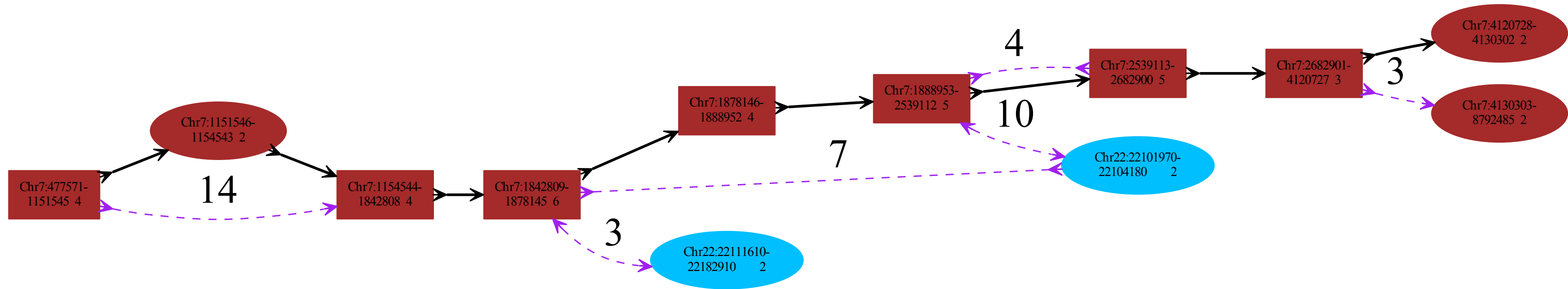

Supplement: Appendix S3 — Reconstruction of K562 genome structure by fusion point guided concatenation method. (ZIP) [file pone.0046152.s019.zip › subgraph38.pdf]

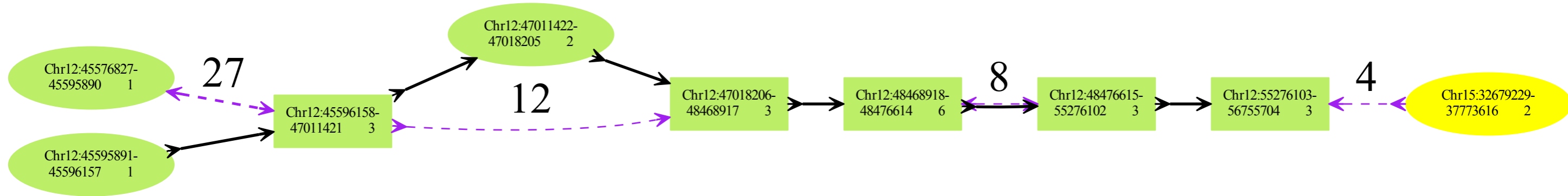

Supplement: Appendix S3 — Reconstruction of K562 genome structure by fusion point guided concatenation method. (ZIP) [file pone.0046152.s019.zip › subgraph39.pdf]

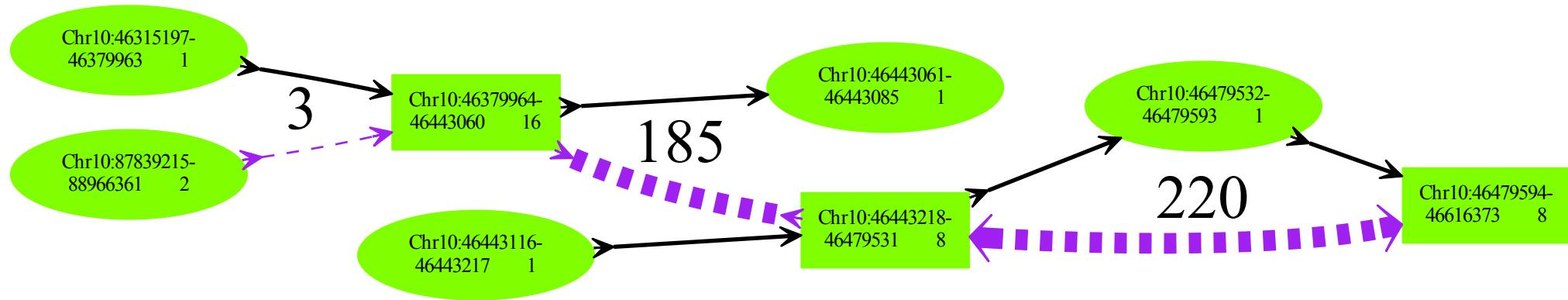

Supplement: Appendix S3 — Reconstruction of K562 genome structure by fusion point guided concatenation method. (ZIP) [file pone.0046152.s019.zip › subgraph4.pdf]

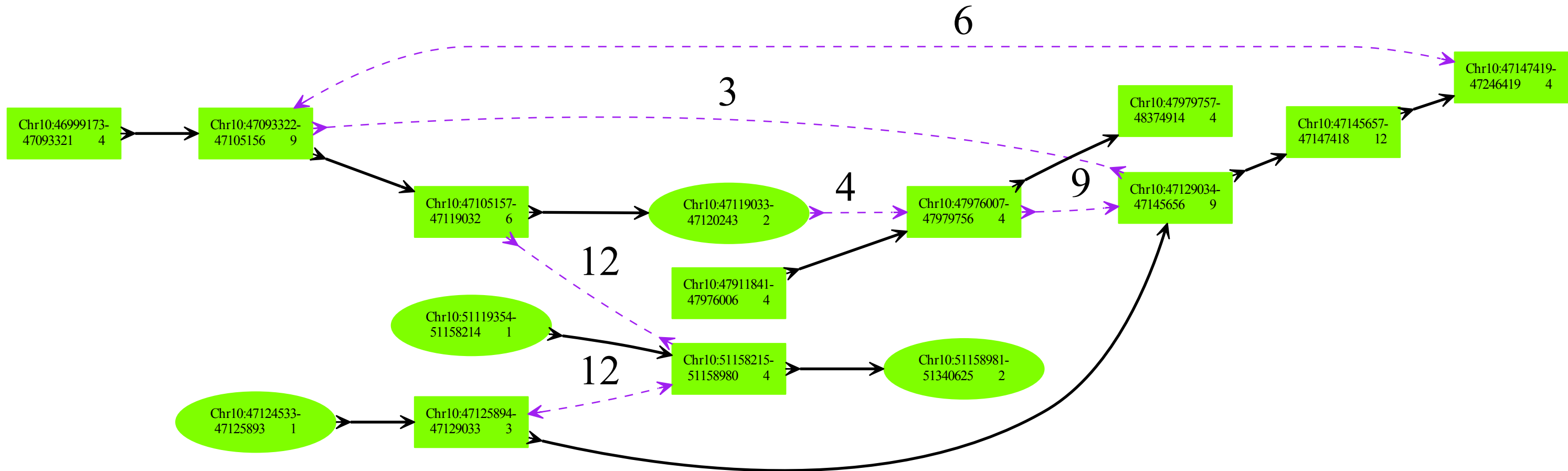

Supplement: Appendix S3 — Reconstruction of K562 genome structure by fusion point guided concatenation method. (ZIP) [file pone.0046152.s019.zip › subgraph40.pdf]

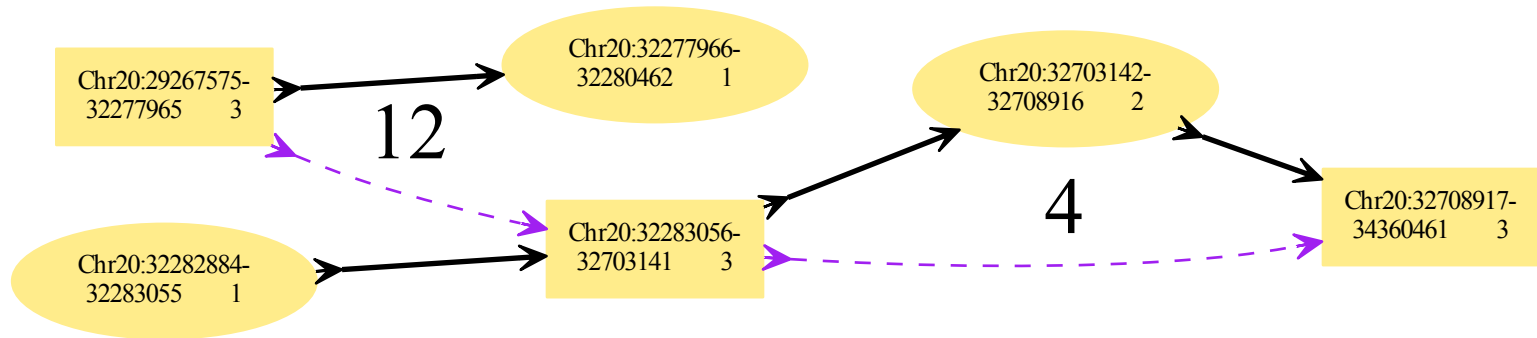

Supplement: Appendix S3 — Reconstruction of K562 genome structure by fusion point guided concatenation method. (ZIP) [file pone.0046152.s019.zip › subgraph41.pdf]

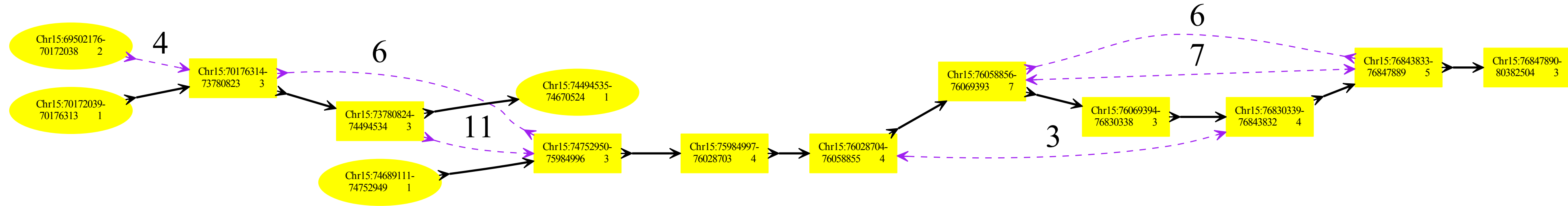

Supplement: Appendix S3 — Reconstruction of K562 genome structure by fusion point guided concatenation method. (ZIP) [file pone.0046152.s019.zip › subgraph42.pdf]

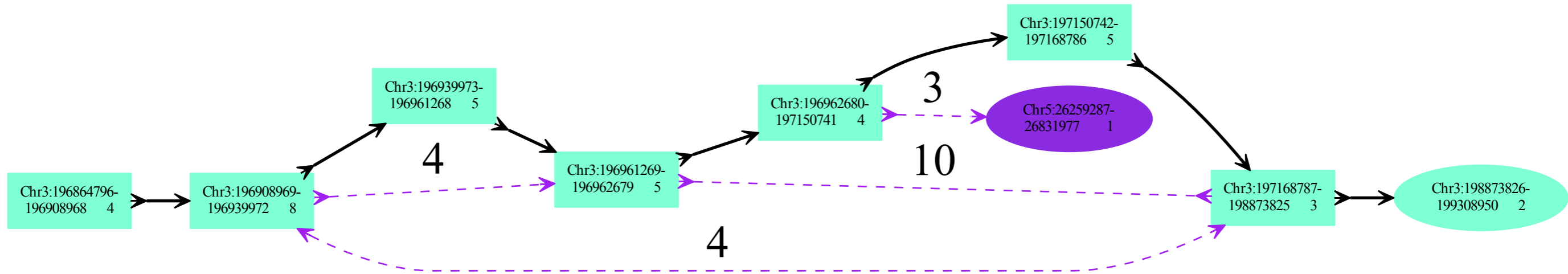

Supplement: Appendix S3 — Reconstruction of K562 genome structure by fusion point guided concatenation method. (ZIP) [file pone.0046152.s019.zip › subgraph43.pdf]

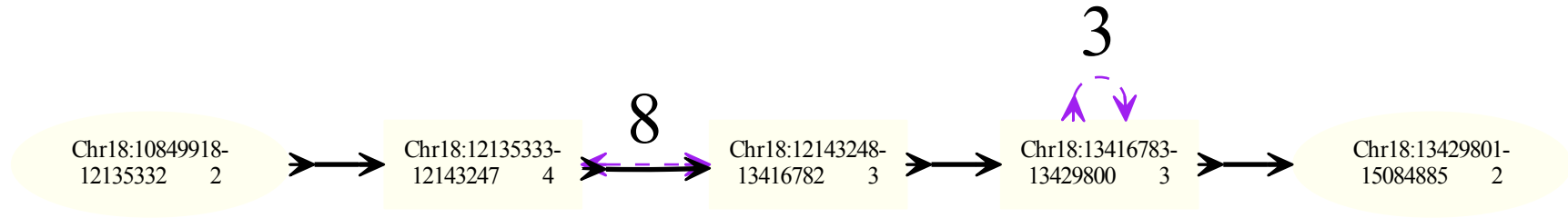

Supplement: Appendix S3 — Reconstruction of K562 genome structure by fusion point guided concatenation method. (ZIP) [file pone.0046152.s019.zip › subgraph44.pdf]

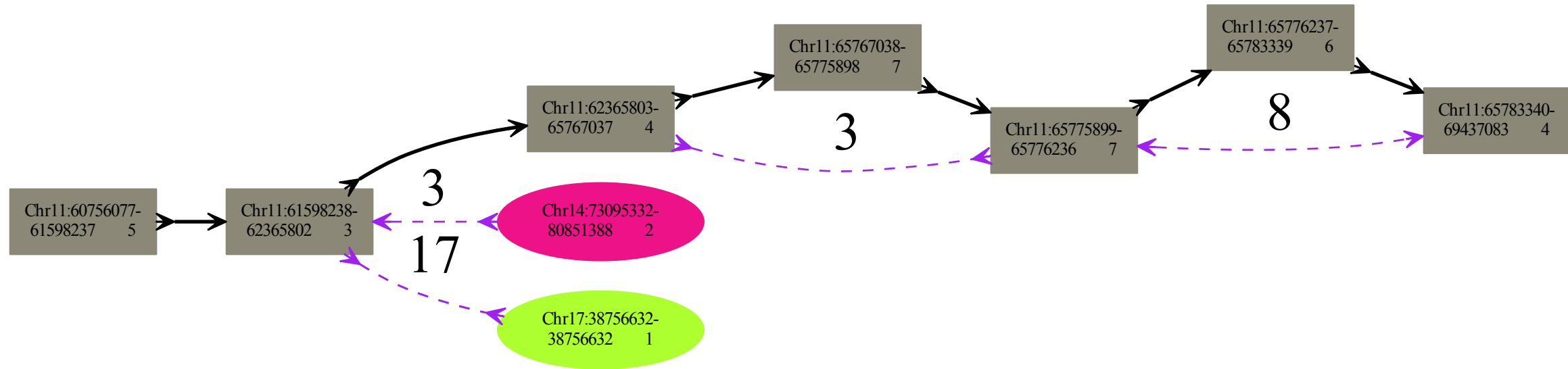

Supplement: Appendix S3 — Reconstruction of K562 genome structure by fusion point guided concatenation method. (ZIP) [file pone.0046152.s019.zip › subgraph45.pdf]

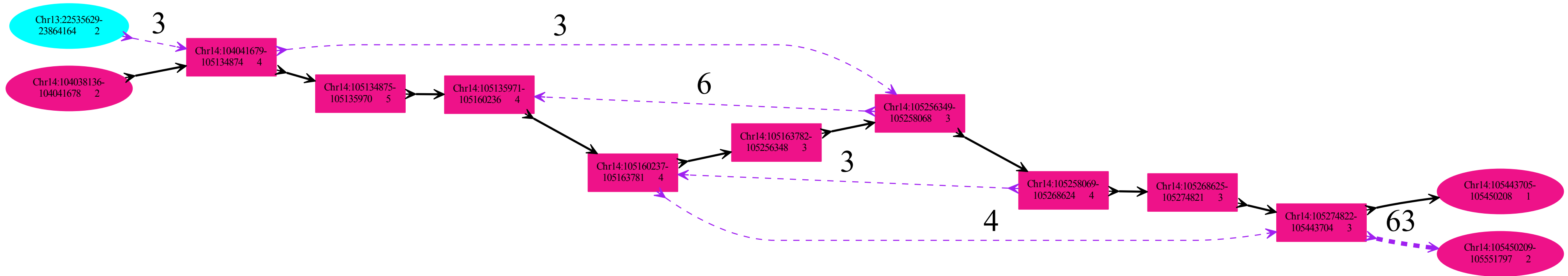

Supplement: Appendix S3 — Reconstruction of K562 genome structure by fusion point guided concatenation method. (ZIP) [file pone.0046152.s019.zip › subgraph46.pdf]

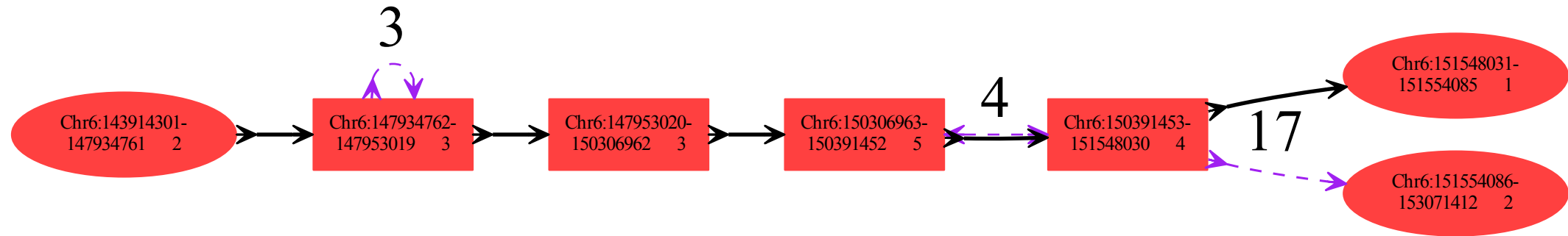

Supplement: Appendix S3 — Reconstruction of K562 genome structure by fusion point guided concatenation method. (ZIP) [file pone.0046152.s019.zip › subgraph47.pdf]

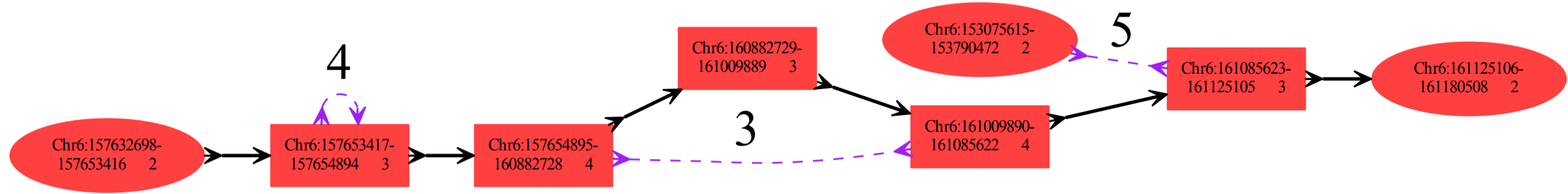

Supplement: Appendix S3 — Reconstruction of K562 genome structure by fusion point guided concatenation method. (ZIP) [file pone.0046152.s019.zip › subgraph48.pdf]

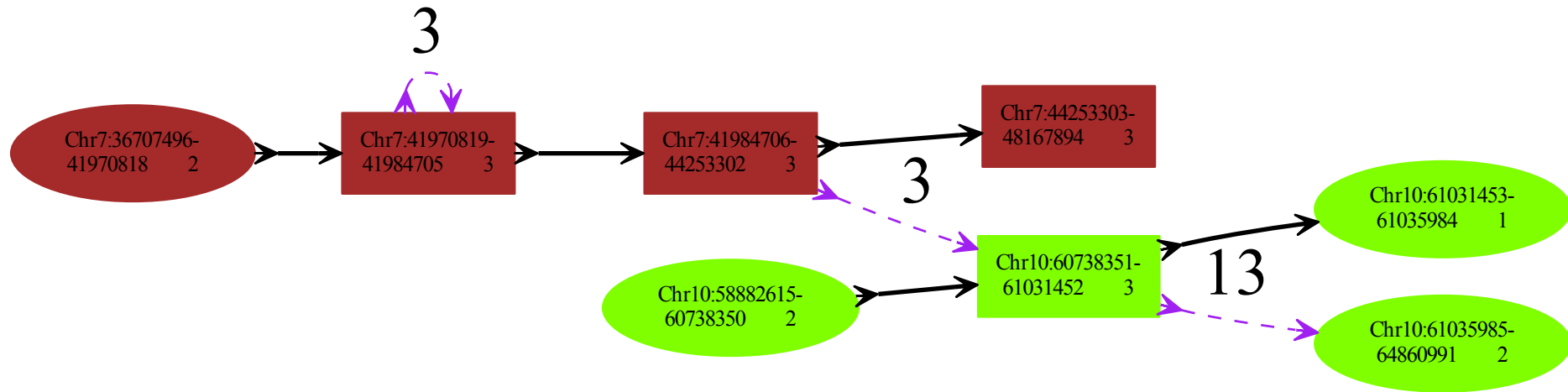

Supplement: Appendix S3 — Reconstruction of K562 genome structure by fusion point guided concatenation method. (ZIP) [file pone.0046152.s019.zip › subgraph49.pdf]

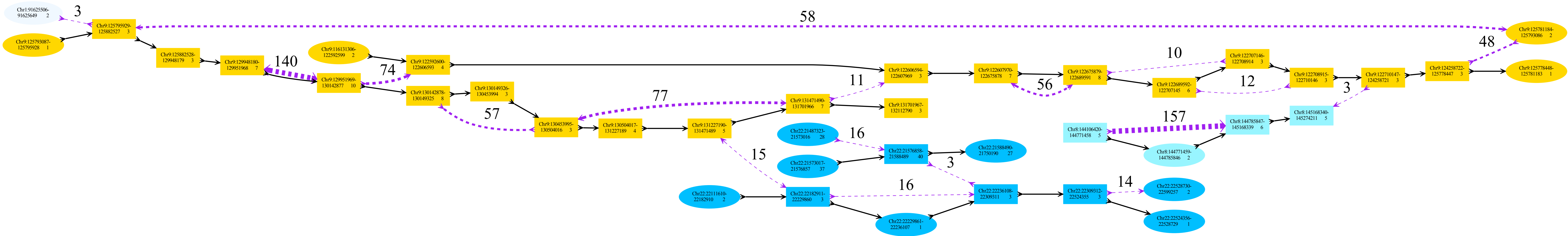

Supplement: Appendix S3 — Reconstruction of K562 genome structure by fusion point guided concatenation method. (ZIP) [file pone.0046152.s019.zip › subgraph5.pdf]

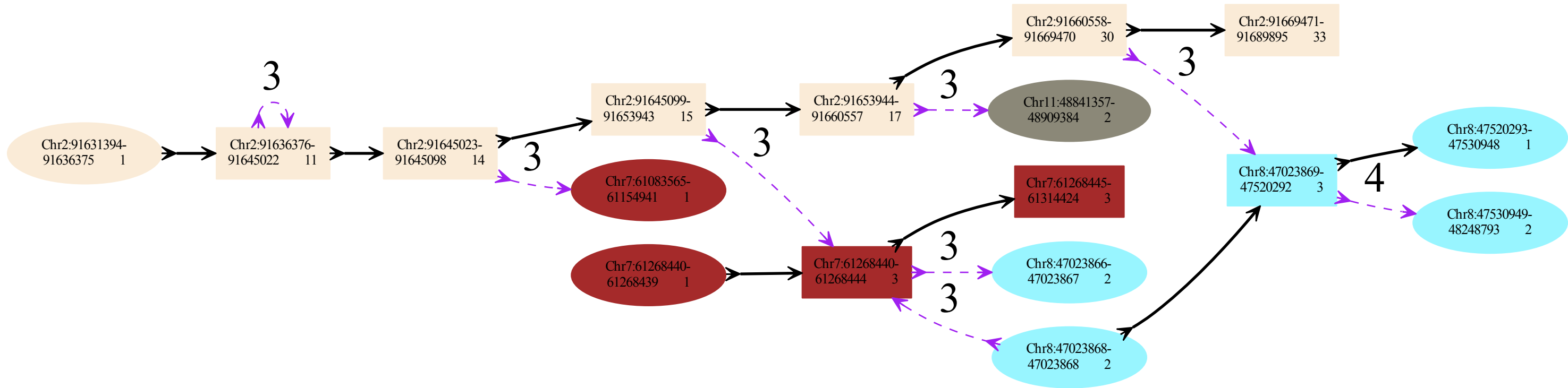

Supplement: Appendix S3 — Reconstruction of K562 genome structure by fusion point guided concatenation method. (ZIP) [file pone.0046152.s019.zip › subgraph50.pdf]

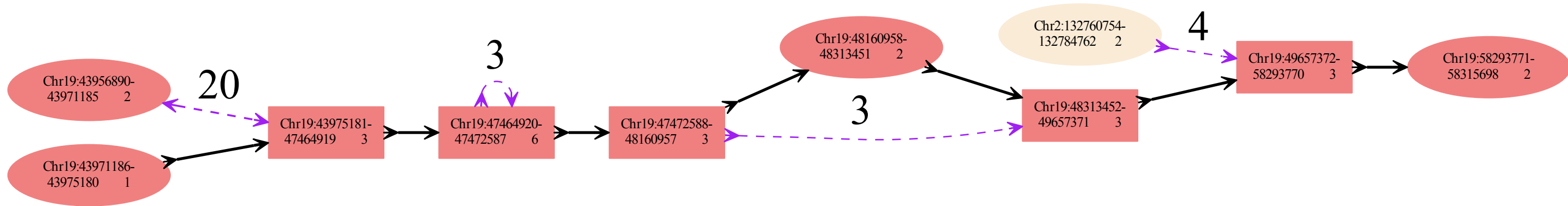

Supplement: Appendix S3 — Reconstruction of K562 genome structure by fusion point guided concatenation method. (ZIP) [file pone.0046152.s019.zip › subgraph51.pdf]

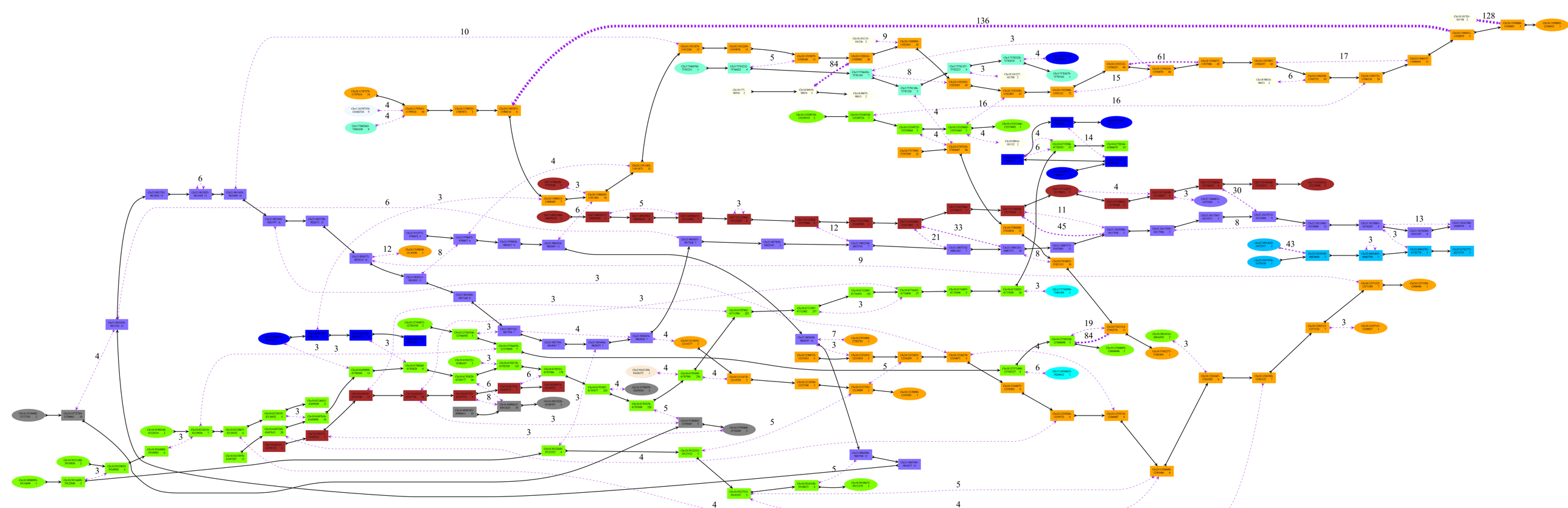

Supplement: Appendix S3 — Reconstruction of K562 genome structure by fusion point guided concatenation method. (ZIP) [file pone.0046152.s019.zip › subgraph6.pdf]

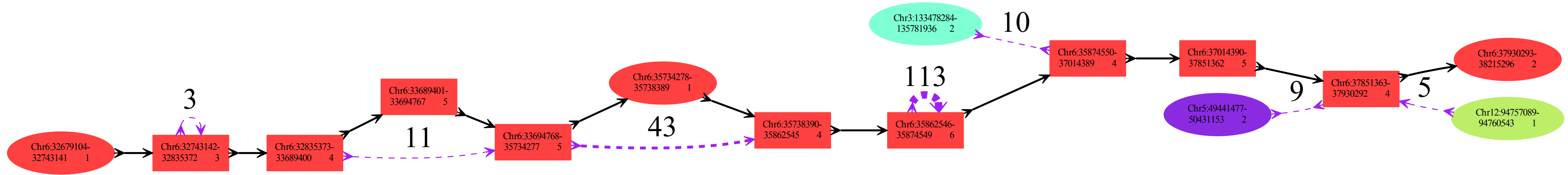

Supplement: Appendix S3 — Reconstruction of K562 genome structure by fusion point guided concatenation method. (ZIP) [file pone.0046152.s019.zip › subgraph7.pdf]

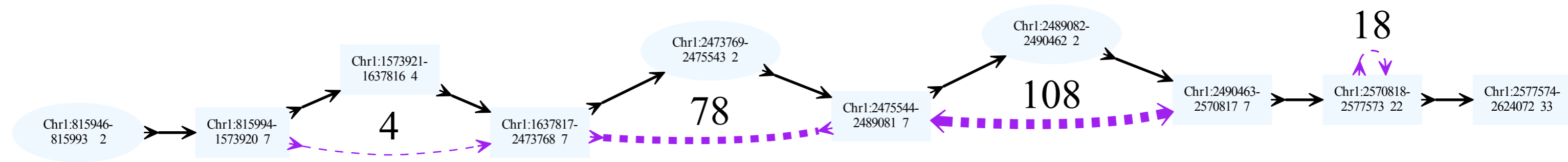

Supplement: Appendix S3 — Reconstruction of K562 genome structure by fusion point guided concatenation method. (ZIP) [file pone.0046152.s019.zip › subgraph9.pdf]
