# Supplementary material for: Long Span DNA Paired-End-Tag (DNA-PET) Sequencing Strategy for the Interrogation of Genomic Structural Mutations and Fusion-Point-Guided Reconstruction of Amplicons
Source: PLoS One. 2012 Sep 28;7(9):e46152. doi: 10.1371/journal.pone.0046152 (PMC3461012; doi:10.1371/journal.pone.0046152)

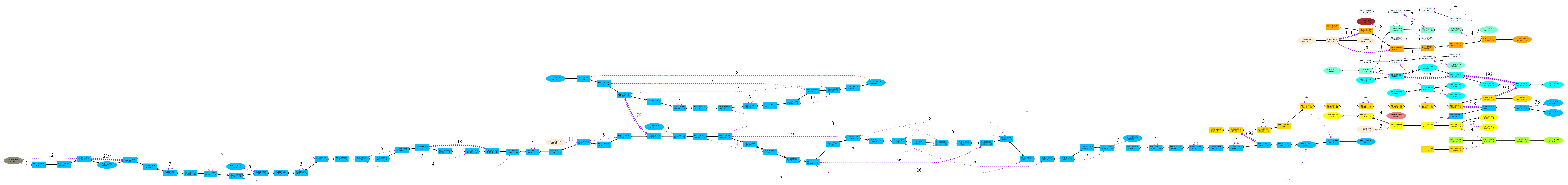

Supplement: Appendix S3 — Reconstruction of K562 genome structure by fusion point guided concatenation method. (ZIP) [file subgraph1.pdf › subgraph1.pdf]
